# Dipeptides of *S*-Substituted Dehydrocysteine as Artzyme Building Blocks: Synthesis, Complexing Abilities and Antiproliferative Properties †

**DOI:** 10.3390/ijms22042168

**Published:** 2021-02-22

**Authors:** Paweł Lenartowicz, Mateusz Psurski, Aleksandra Kotynia, Aleksandra Pieniężna, Monika Cuprych, Klaudia Poniatowska, Justyna Brasuń, Paweł Kafarski

**Affiliations:** 1 Faculty of Chemistry, University of Opole, ul. Oleska 48, 45-052 Opole, Poland; 2 Laboratory of Experimental Anticancer Therapy, Hirszfeld Institute of Immunology and Experimental Therapy, Polish Academy of Sciences, ul. R. Weigla 12, 53-114 Wrocław, Poland; mateusz.psurski@hirszfeld.pl (M.P.); monika.cuprych@hirszfeld.pl (M.C.); k.poniatowska96@gmail.com (K.P.); 3 Department of Inorganic Chemistry, Wrocław Medical University, ul. Borowska 211 A, 50-556 Wrocław, Poland; aleksandra.kotynia@umed.wroc.pl (A.K.); aleksandra.pieniezna@umed.wroc.pl (A.P.); justyna.brasun@umed.wroc.pl (J.B.); 4 Department of Bioorganic Chemistry, Wrocław University of Science and Technology, Wybrzeże Wyspiańskiego 27, 50-370 Wrocław, Poland

**Keywords:** dehydrocysteine, dehydropeptides, addition-elimination reaction, complexing agent, antiproliferative activity

## Abstract

Background: Dehydropeptides are analogs of peptides containing at least one conjugate double bond between α,β-carbon atoms. Its presence provides unique structural properties and reaction centre for chemical modification. In this study, the series of new class of dipeptides containing *S*-substituted dehydrocysteine with variety of heterocyclic moieties was prepared. The compounds were designed as the building blocks for the construction of artificial metalloenzymes (artzymes). Therefore, the complexing properties of representative compounds were also evaluated. Furthermore, the acknowledged biological activity of natural dehydropeptides was the reason to extend the study for antiproliferative action of against several cancer cell lines. Methods: The synthetic strategy involves glycyl and phenylalanyl-(*Z*)-β-bromodehydroalanine as a substrate in one pot addition/elimination reaction of thiols. After deprotection of N-terminal amino group the compounds with triazole ring were tested as complexones for copper(II) ions using potentiometric titration and spectroscopic techniques (UV-Vis, CD, EPR). Finally, the antiproliferative activity was evaluated by sulforhodamine B assay. Results and Conclusions: A simple and efficient procedure for preparation of dipeptides containing *S*-substituded dehydrocysteine was provided. The peptides containing triazole appeared to be strong complexones of copper(II) ions. Some of the peptides exhibited promising antiproliferative activities against number of cancer cell lines, including cell lines resistant to widely used anticancer agent.

## 1. Introduction

Nature utilizes twenty amino acids set to generate an array of proteins with diverse structures and functions. Amongst them are extremely efficient catalysts in living organisms—enzymes. Despite of that, there is a large number of enzymes there are also some reactions with not known enzymatic equivalent. Thus, the premise of constructing enzyme-like catalysts, which are capable of efficient catalysis of virtually any chemical reaction is a tremendous challenge. It is not surprising that intensive efforts have been undertaken to obtain enzyme mimetics and that various strategies of their design have been described [1,2,3,4]. Such bioinspired catalysts are broadly termed artificial enzymes or “artzymes” [3,5,6]. One class of these molecules is constructed in such a manner that the peptide folds (in most cases foldamer helical segments) guarantee specific architecture of the catalyst, while amino acids side chain structures are responsible for the enzyme-like activity [7,8,9,10,11,12]. This allows to construct catalytic active sites not seen in nature. Such artzymes are commonly constructed by the stepwise joining of small peptide units (most likely foldamers) of variable sequences [13,14,15,16].

The main goal of the project is the synthesis of dipeptides designed as building blocks of foldamers to be further exploited in artzyme preparation. For that, functionalization of peptides containing *C*-terminal dehydroalanine with heterocyclic thiols, leading to *S*-substituted dehydrocysteine derivatives was chosen as a first approach. The obtained peptides are designed for the construction of mimetics of metallo-enzymes. Thus, we have checked whether the chosen representatives possess abilities to complex copper(II) ions. Since some dehydropeptides are known as natural biologically active compounds and exhibit antibiotic, antifungal, antitcancer, phytotoxic and antiproliferative activity [17,18] we had decided to extend our study towards the evaluation of the antiproliferative activity of the obtained compounds against several cancer cell lines.

## 2. Results and Discussion

### 2.1. Syntheses

**Scheme 1:**
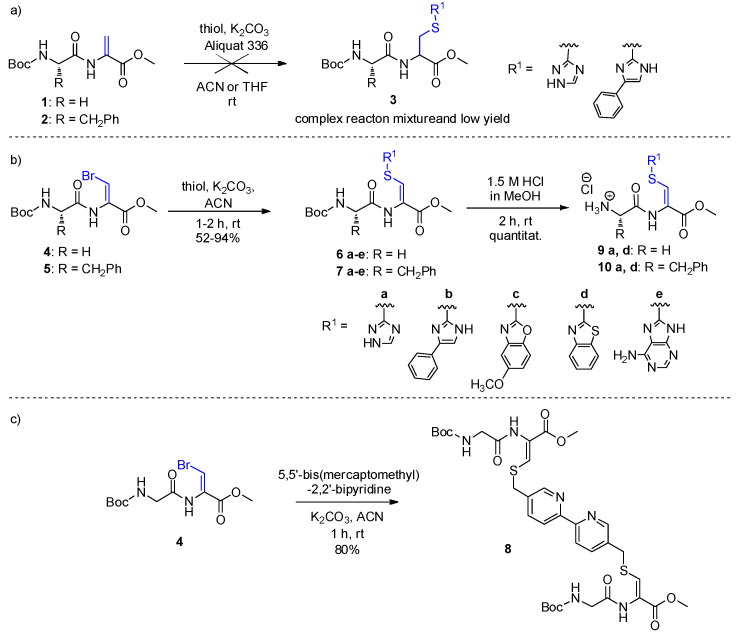
Syntheses of dipeptide building blocks containing (**a**) *C*-terminal cysteines; (**b**) *C*-terminal dehydrocysteines; (**c**) cross-linked dehydrocysteines.

Thiols readily underwent Michael addition to *N*-protected glycyl-dehydroalanine (compound **1**) and phenyl-dehydroalanine (compound **2**) providing micromolar inhibitors of cathepsin C [19]. Thus, the first approach in this work was to use this reaction for the synthesis of the desired peptides containing *C*-terminal cysteine *S*-functionalized with various heterocycles (compounds **3**; Scheme 1a). Surprisingly this approach appeared to be completely unsuccessful.

Recently we had found that the addition of bromine to compounds **1** and **2**, followed by elimination of the bromine atom by the action of triethylamine provided dehydropeptides **4** and **5** containing (*Z*)-β-bromodehydroalanine [20]. These peptides by being crystalline and stable upon storage had been considered as ideal substrates for addition-elimination reaction and provided readily a series of dehydropeptides (compounds **6** and **7**; Scheme 1b). The whole procedure is simple and results in the desired dehydropeptides in good yields (Table 1).

Additionally, we have used 5,5-bis(mercaptomethyl)-2,2′-bipyridine as a substrate and obtained product crosslinking of the two peptide chains (Boc-Gly-ΔCys(*S*,*S*-bismethylbipyridine)-OMe, compound **8**, Scheme 1c). Removal of Boc-protection from four representative examples of the obtained dehydropeptides by using the solution of hydrochloride in methanol resulted in the preparation of compounds **9** and **10** (Scheme 1b). The stereochemistry of the obtained peptides was assigned as *Z*, basing on NMR studies (for details please see Supplementary Materials).

It is worth to mention, that this simple synthetic protocol enables to incorporate aliphatic and aromatic thiols into the structure of a peptide. Therefore, this reaction seems also to be useful in late-stage functionalisation of peptides. According to our knowledge, the designed dehydrocysteine derivatives have not equivalent in naturally occurring dehydropeptides. Cysteine could rather be converted to dehydroalanine by elimination reaction. The incorporation of heterocyclic moiety into the side chain of dehydrocysteines is rarely described [21,22] and this modification should affect the biological and coordination properties o dehydropeptides. 

### 2.2. Complexation of Cu^2+^ Ions by Compounds ***9a*** and ***10a***

Fully blocked peptides **6** and **7** appeared to be very weak complexones for Cu^2+^ ions. This is probably because there is a lack of moiety anchoring copper(II) ions. On the contrary, their unblocked counterparts, compounds **9** and **10**, appeared to be quite effective complexones. The characteristic feature of the dehydropeptides containing *S*-substituted (*Z*)-dehydrocysteines is their very rigid structure and low flexibility. 

**Figure 1:**
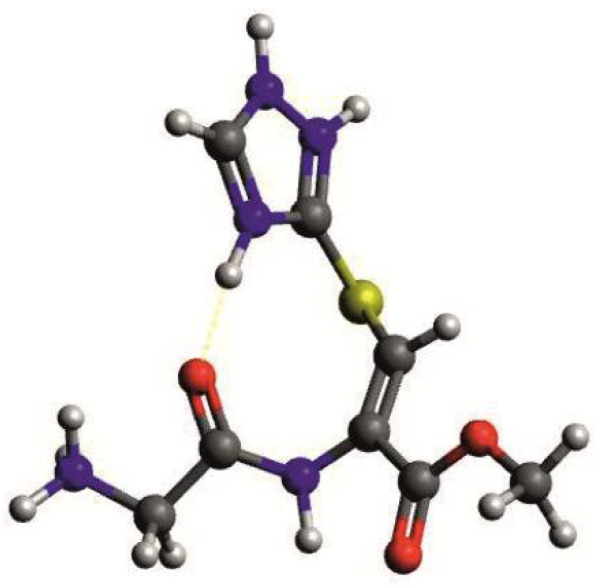
The proposed structure of the fully protonated **9a** ligand.

Being the structurally simplest representatives, compounds **9a** and **10a** have been chosen for a more detailed study of complexation mode. Both studied ligands are characterized by three protonation constants (Table 2). The log*K* values of 7.96 (for **9a**) and 8.11 (for **10a**) are typical for the constants related to the free *N*-terminal amino group in short peptides [23,24,25,26,27]. The next two constants (log*K* ≈ 10.3 and ≈7.0) result from the presence of the 1,2,4-triazole ring, however, they are significantly higher in comparison to the constants found for the free triazole ring (log*K* ≈ 9.5 and ≈2.5) [28,29,30]. This may be explained by the possible formation of the intramolecular hydrogen bond between one of the triazole protons and the oxygen atom from the neighboring peptide bond (Figure 1).

**Figure 2:**
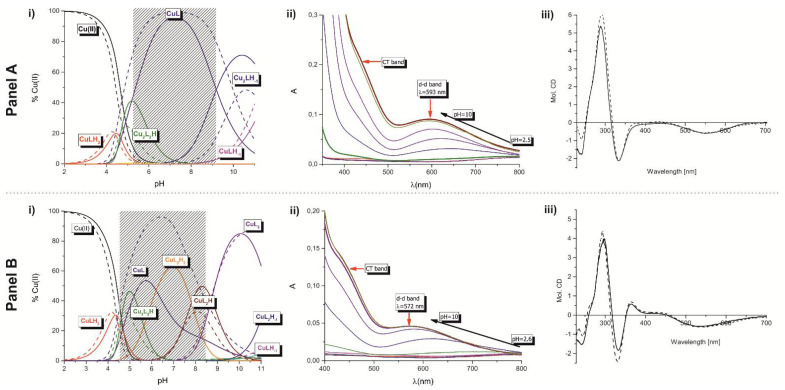
**Panel A:** (**i**) the species distribution curves for the **9a**-Cu(II) system (solid line) and **10a**-Cu(II) (dashed line) in dependence on pH, measured for the nL:nCu(II) = 1:1 molar ratio, (**ii**) the UV-Vis spectra in dependence on pH for the **9a**, (**iii**) the CD (dashed line) and MCD (solid line) spectra of **10a** with the nL:nCu(II) = 1:1 molar ratio at pH = 10.5. **Panel B:** (**i**) the species distribution curves for the **9a**-Cu(II) (solid line) and **10a**-Cu(II) (dashed line) system in dependence on pH, measured for the nL:nCu(II) = 2:1 molar ratio, (**ii**) the UV-Vis spectra in dependence on pH for the **9a**, (**iii**) the CD (dashed line) and MCD (solid line) spectra of **10a** with the nL:nCu(II) = 2:1 molar ratio at pH = 10.5.

The coordination studies of **9a** and 1**0a** were performed for two systems of different ligand-to-copper(II) ion molar ratio, namely 1:1 and 2:1. For the **10a** the precipitation was observed in the pH range of 4.5 and 8.0 (Figure 2, panel A) and thus the study for this ligand was focused on two ranges of pH: 2–4.5 and 8.5–10.0. It is worth to mention that the comparison of the potentiometric and spectroscopic results received for both **9a** and **10a** showed the same coordination pattern.

**Figure 3:**
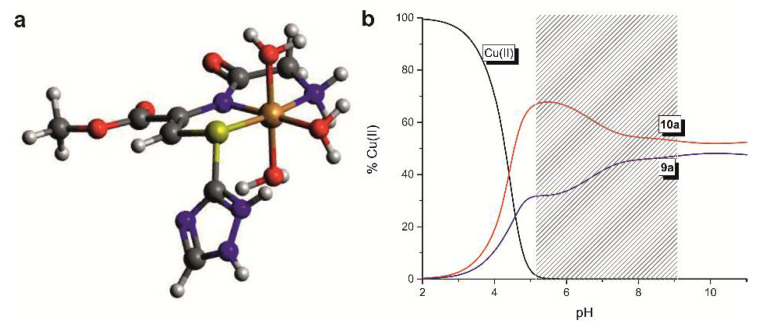
(**a**) The proposed structure of the CuL species formed by the **9a**, (**b**) the competition diagram for the **9a**:Cu(II):**10a** system with the nL1:nCu(II):nL2 = 1:1:1 molar ratio.

In the equimolar conditions at pH range between 3.0 and 10.0, five complexes exist with the CuLH_x_ and Cu_2_L_2_H_x_ stoichiometries (Figure 2, panel A, Table 2). The coordination begins around pH 3.0 with the formation of the CuH_2_L complex. Its appearance is related to the dissociation of one proton from the ligand. Because of their low concentration in the solution, it was not possible to receive the spectroscopic parameters. Nevertheless, the corrected stability constant (log*β* = log*β*_CuLH2_ − log*β*_H2L_) equal 5.47 supports involvement of the *N-*terminal amino group and formation of the species with the {N_-NH2_, O_-CO_} binding mode, which is characteristic for the linear peptides with free *N*-terminal groups [31,32,33]. With the increase of the pH the next monomeric CuL species appears, what significantly influences the spectral characteristics of the system with the **9a** ligand (Figure 2, panel A). The location of the d-d band at 593 nm with molar extinction coefficient ε = 73 M^−1^cm^−1^ supports binding of two nitrogen donors to the metal ion. Moreover, the location of the CT band in the UV-Vis at 430 nm supports coordination of the sulfur donor. Its creation results from the release of two protons from the CuLH_2_. There are two possibilities of the course of this process: (i) both protons dissociate from the triazole ring or (ii) one proton derives from triazole ring and the second one from the peptide bond. Thus, the CuL system may be described either by the {N_-NH2_, N_triazole_, S} or {N_-NH2_, N_amide_, S} binding modes, respectively. The simultaneous coordination of the S-donor and deprotonated N_triazole_ would create the four-member chelating ring what is less thermodynamically favorable than the formation of the five-member ring, which is expected in the case of N_amide_ binding. Due to this, and in relation to the already known coordination abilities of the dehydrodipeptides [34], the {N_-NH2_, N_amide_, S} coordination manner is more probable. Based on this assumption, one of the triazole nitrogen atoms is still protonated. The value of log*β*_CuL_, independent of the log*K*_triazole_: logβ* = log*β*_CuL_ − log*K*_HL_ is equal to 4.09 and is significantly higher than log*β* = 2.19 calculated for the analogous Gly(Z)ΔPhe species with the {N_-NH2_, N_amide_} binding mode [34]. It strongly supports the involvement of the sulfur atom and formation complex where N_-NH2_, N_amide_, and S donors are placed within the plane (Figure 3a). The formation of the last, CuLH_-1_ complexes in both systems (with the **9a** and **10a** ligands) does not influence significantly the spectral abilities. The value of the log*K* for the formation of the CuLH_-1_ is comparable to the constants related to the proton dissociation from the second triazole nitrogen from the free ligands (Table 2). It strongly supports that this process does not influence the coordination mode of the copper(II) ion.

Compounds **9a** as well as **10a** also form complexes with the Cu_2_L_2_H_x_ stoichiometry: Cu_2_L_2_H and Cu_2_L_2_H_-1_, with their highest concentrations being observed at pH 5.5 and 10.5, respectively. Both of them are created by the interaction between two mononuclear species (CuHL + CuL → Cu_2_L_2_H, CuL + CuLH_-1_ → Cu_2_L_2_H_-1_). Their appearance is related to the triazole protonation states and seems to support the assumption that it is the result of the formation of intermolecular hydrogen bonds involving H_triazoles_. Additionally, the observation that there is no significant change in the spectral abilities of the system with dominating Cu_2_L_2_H_-1_, suggest the same coordination mode as in the case of CuL complexes (Figure 2, panel A). In the case of **10a** ligand, which *N*-terminal amino acid is chiral, there was possible to extend the studies by using CD technique. Studies were carried out at pH of 10.5. Owing to the fact, that this molecule possesses the aromatic system, also the magnetic circular dichroism was applied to record the MCD_pH10.5_ spectrum, however it did not bring additional information. Anyway, the positive CT band at 290 nm and the negative at 344 nm (Figure 2, panel A) confirm the presence of two nitrogen donors: N_-NH2_, N_amide_, in copper(II) coordination sphere.

Summing up, **9a** and **10a** show similar binding abilities, but the presence of the phenylalanine in the structure of the latter enhances the efficiency of Cu(II) binding. Below pH 5 the **10a** is significantly more effective binder of Cu(II) but with the increase of the solution basicity, the efficiency of copper(II) coordination of both ligands slowly equals (Figure 3b).

**Figure 4:**
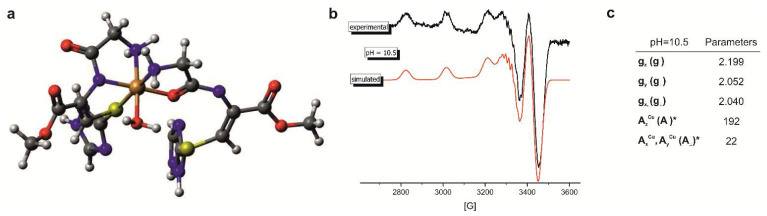
(**a**) The proposed binding mode of the **9a**-Cu(II) in the complexes with the CuL_2_H_x_ stoichiometry (**b**) the experimental and simulated EPR spectra and (**c**) parameters obtained for the **9a** ligand in the system with nL:nCu(II) = 2:1 molar ratio and pH = 10.5.

As the next, the studies of the system with the double excess of ligands have been performed (Figure 2 panel B). The analysis of the potentiometric and spectroscopic results show that both compounds, **9a** and **10a**, begin copper(II) binding by the creation of the same complexes as it was discussed above. The complexes with CuL_2_H_x_ stoichiometry appear above pH 6.5 (for the **9a**) and pH 8.5 (for the **10a**). In the system with **9a** ligand any significant changes in the spectral abilities of the system are not observed, what strongly supports that: (i) the binding mode of Cu(II) is the same in these complexes and (ii) theirs formation is related to triazole’s protons dissociation. The comparison of the location of the d-d band in the previous system shows a blue shift (λ_max 1:1_ = 593 nm → λ_max 2:1_ = 572 nm, Figure 2). It confirms coordination of the third nitrogen donor to the metal ion. Moreover, the positive LMCT transition at 364 nm strongly supports the presence of sulphur in the coordination sphere of Cu(II). The most likely is the formation of the complex with the {2xN_-NH2_, N_amide_, S} binding mode (Figure 4a). 

For a better understanding of the discussed system, the EPR spectrum was recorded at pH 10.5 for the **9a** with 2:1 ligand-to-metal molar ratio (Figure 4b). The observed A_II_ = 192 [G] g_II_ = 2.199 are relatively high but the seven lines in the high-field region of spectrum strongly confirm binding of three nitrogen donors, while the components of g tensor (g_z_ >> g_y_,g_x_ > 2.0023) supports the axial symmetry. Moreover, the characteristic A_II_^Cuv^ >> vA_⊥_^Cu^ splitting pattern suggests the d_x2-y2_ ground state [35].

As it was observed previously for the system of 1:1 stoichiometry the presence of phenylalanine residue evidently influenced the efficiency of the copper(II) binding in the acidic conditions. In ligand to Cu(II) ions of 2:1 molar ratio, the presence of the aromatic ring in the side chain effectively hinders the formation of the complexes with the CuL_2_H_x_ stoichiometry and promotes the formation of the CuL complex. This ability of the **10a** may be related to the protection of one of the axial positions in the coordination sphere of the Cu(II) by the side chain of phenyl residue.

**Figure 5:**
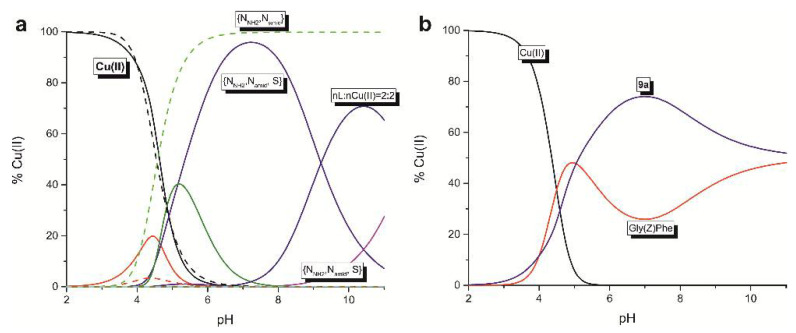
The comparison of: (**a**) formation of subsequent complexes for **9a** (solid line) and Gly(*Z*)Phe (dashed line) in dependence on pH, (**b**) efficiency in copper(II) binding for the n_L_:n_Cu(II)_ = 1:1 molar ratio.

Compounds **9a** and **10a** might be considered as dipeptides containing C-terminal mimetics of dehydrophenylalanine. Insertion of a dehydroamino acid residue into a sequence of peptide changes considerably the binding abilities of peptide ligands towards copper(II) ions [32,33,34]. The presence of the double bond influences acidity of vicinal amide function and thus the binding of amide nitrogen is favored, which makes the dehydropeptides bind metal ions significantly more effectively. Additionally, type of geometric isomer has a critical impact on the coordination equilibria, with (Z) isomers being more effective ligands. The comparison of the binding abilities between **9a** and Gly(Z)Phe (Figure 5a) shows that at pH 7.4, both peptides form complexes via utilization of two nitrogens donors: *N*-terminal amino group and an amide. However, due to the presence of the additional donor, sulfur atom, the ligand **9a** is significantly more effective in Cu(II) coordination (Figure 5b). Thus, newly synthesized dehydropeptides **9a** and **10a** represent a new type of complexones for metal ions and thus could be utilized in the design and production of artzymes

### 2.3. Antiproliferative Activity of Synthesized Compounds

Since the dehydropeptides reveal variable biological activity, we decided to screen the synthesized compounds for antiproliferative activity *in cellulo* utilizing ten diverse human cancer cells lines and murine non-tumorigenic cell line (Balb/3T3). The results shown in Table 3 and Table 4 indicated that most of the compounds exhibited promising activities in comparison to widely used anticancer agent cisplatin (CDDP). In Table 3, results obtained for the following cell lines are shown: breast adenocarcinoma MCF-7, breast carcinoma MDA-MB-231, lung carcinoma A549, colorectal adenocarcinoma LoVo, and its doxorubicin-resistant variant LoVo^DX^. Table 4 presents the results obtained for UM-UC-3 urinary bladder transitional cell carcinoma and its variants resistant to cisplatin, gemcitabine, vinblastine, and the cell line resistant both to cisplatin and gemcitabine.

Selected compounds exhibited high antiproliferative activity with **6c** and **6d** and **7c** and **7d** recognized as the most potent representatives for glycine and phenylalanine derivatives, respectively. In both groups, derivatives containing triazole (**6a** and **7a**) and adenine (**6e** and **7e**) showed markedly lower, if any, activity. These two observations clearly indicate that *N*-terminal amino acid (glycine or phenylalanine) has negligible influence on activity in contrast to *C*-terminal dehydrocysteine for which significant changes in antiproliferative activity versus structure of side chain were observed. *N*-Boc deprotection in compounds **9** and **10** was accompanied by a dramatic decrease in antiproliferative activity, which is an expected result of the introduction of free -NH_2_ group to the compound that most likely is not influxed by any specialized transporter. No cell line specificity was observed for any of compounds tested, including non-tumorigenic Balb/3T3 cell line and cell lines characterized by drug-resistant phenotype. Comparable activity of the studied compounds against LoVo, UM-UC-3, LoVo^DX^ and UM-UC-3^VBL^ (last cell lines characterized by high P-gp expression [36]) strongly suggests that studied dipeptides of S-substituted dehydrocysteine are not substrates for multidrug resistance pumps. Their comparable activity on cisplatin-resistant cell lines characterized inter alia by increased reduced glutathione level, which in turn result in increased inactivation of electrophilic moieties indicates that these compounds are not a subject of II phase detoxification enzyme systems.

The previous report on the structure–activity relationship of structurally simple dehydroamino acids derivatives (dehydroalanine, dehydroaminobutyric acid, and dehydrophenylalanine) containing a variety of N-protection revealed that toxicity on the selected human cancer cell lines is related to their lipophilicity and the electron-withdrawing effect of protecting group. Moreover, the results of the biochemical tests performed for selected compounds indicate that caspase-dependent apoptosis is a crucial component of their mode of action [18]. The comparison of toxicity of dehydroamino acid derivatives against lung carcinoma cell line (A549) shows that the most of the S-substituted dehydrocysteine dipeptides presented in this paper seem to be more effective (Table 3 vs. literature IC_50_ > 62.5 µM) [18]. It is also worth mentioning that compound **10d** is more effective against resistant cell lines (with exception of one resistant to gemcitabine) as compared to the parent cell line. Compounds **6c**, **6d**, **7c**, and **7d** are equipotential towards both resistant and parent cell lines. The exact mechanism of action of these structurally modified dehydropeptides is unknown, and extensive studies on its determination are underway. Further studies will also be conducted to assess the validity of these compounds as potential anticancer agents.

## 3. Materials and Methods

### 3.1. General Procedure for Preparation of Dipeptides Containing S-Substituted Dehydrocysteine

The peptide substrate containing *Z*-bromodehydroalanine (**4** or **5**, 1 eq) was dissolved in acetonitrile then appropriate thiol (1.05 eq) and K_2_CO_3_ (2 eq) were added. The reaction mixture was stirred at ambient temperature until the peptide substrate was consumed (controlled by TLC, usually 1–2 h). The volatile components of the reaction mixture were removed by rotary evaporator. The residue was purified by flash chromatography. The product was crystallized or precipitated from the appropriate solvent mixture. Detailed information, including amounts used as well characterization of synthesized compounds, is given in Supplementary Materials.

### 3.2. General Procedure for Boc Deprotection

The dipeptide was stirred with the solution of HCl in methanol (1.5 M) for 1–2 h at ambient temperature. The conversion of the substrate was monitored by TLC. If necessary, the reaction mixture was decolorized with activated carbon. The volatile component was co-evaporated with toluene and DCM under reduced pressure. Finally, the product was lyophilized from aqueous solution (for details please see SM). 

### 3.3. Potentiometric Titration

The pH-metric measurements were performed on Metrohm 905 Titrando system equipped with combined Biotrop^®^ electrode. The titrant was 0.1 M KOH (Merck, Germany) freed of CO_2_ by bubbling Ar gas for 3 h. The electrode calibration was performed before each titration. The volume of all the samples was 1.5 mL and the measurements were carried out at a range of pH of 2.5–11.0 in thermostated vessels at 298 K in an inert atmosphere of Ar gas. The ligands **9a** and **10a** were diluted in hydrochloric acid (pH = 2.5), prepared from 37% HCl (Sigma-Aldrich, Poland). Their concentration was set up at a range of 1.0–2.0 × 10^−3^ M and the ionic strength of 0.1 M was achieved by the addition of KCl (Sigma-Aldrich, Poland). The ligand-metal ion systems were prepared by addition of CuCl_2_ (Sigma-Aldrich, Poland). stock solutions to reach final ligand to metal ratio (nL: nCu^2+^) being 2:1 or 1:1.

The protonation (β_i_ = [H_i_L]/[H^+^]^i^[L]), stability constants (β_pqr_ = [M_p_H_q_L_r_]/[M]^p^[H]^q^[L]^r^), and stoichiometry of complexes were calculated using the HYPERQUAD program [37]. The distribution species plots were made by HySS program [38]. The standard deviations were computed for all the constants and indicated the presence of only random errors, which was a good evidence of the presence of each species in the equilibrium.

### 3.4. Spectroscopic Measurements (UV-Vis, CD, EPR)

The UV-Vis absorption measurements were recorded on Varian Carry 50 Bio spectrophotometer. All spectra were collected in a quartz cuvette with 1 cm path length at 298 K. The spectral range was 300–800 nm with resolution 0.1 nm. The molar extinction coefficients (ε [M^−1^cm^−1^]) were calculated for each spectrum at a wavelength of maximum absorption.

The Circular Dichroism spectra (CD) and Magnetic Circular Dichroism (MCD) were measured on a JASCO J 600 spectropolarimeter in a range of 230–800 nm with 0.1 nm resolution. The optical length of the quartz cuvettes was 10 mm. The molar circular dichroism coefficient (Δε [M^−1^cm^−1^]) was calculated for each spectrum.

The Electron Paramagnetic Resonance (EPR) spectra were recorded on a Brucker ESP 300E spectrometer at X-band frequency (9.5 GHz) at 77 K obtained by the use of liquid nitrogen. The procedure of sample preparation for spectroscopic measurements was the same as in potentiometric titration. The EPR samples were protected by adding 30% of ethylene glycol (Sigma-Aldrich, Poland). The pH of the samples was determined by adding a small quantity of a concentrated solution of KOH (Merck, Germany) to achieve the desired value and controlled by Mettler Toledo pH-meter equipped with combined electrode InLab^®^Micro. The simulation of the EPR spectrum was performed by the Hyperfine Spectrum program written by W.R. Hagen [39].

### 3.5. Visualization of Complex Structures

The proposed structures of complexes were drawn and optimized by using Avogadro: an open-source molecular builder and visualization tool, version 1.2.0 (http://avogadro.cc/) using universal field force [40].

### 3.6. Antiproliferative Activity Assessment

#### 3.6.1. Cell Lines and Cultures Conditions

Ten different human cancer cell lines (including drug-resistant sublines) and one non-tumorigenic murine fibroblasts cell line were used to determine antiproliferative activity of the tested compounds. The MDA-MB-231 (breast adenocarcinoma), UM-UC-3 (urinary bladder transitional cell carcinoma), LoVo (colorectal adenocarcinoma), LoVo^Dx^ (colorectal adenocarcinoma, doxorubicin-resistant) and Balb/3T3 (non-tumorigenic murine fibroblasts) were purchased from the American Type Culture Collection (ATCC; Rockville, USA). The MCF-7 (breast adenocarcinoma) and A549 (lung carcinoma) were purchased from European Collection of Authenticated Cell Cultures (ECACC; Porton Down, UK). The UM-UC-3^CDDP^, UM-UC-3^GEM^, UM-UC-3^VBL^, and UM-UC-3^CDDP/GEM^ cell lines were established at Hirszfeld Institute of Immunology and Experimental Therapy of the Polish Academy of Sciences (HIIET, PAS, Wroclaw, Poland) by the continuous exposure to the increasing concentrations of corresponding cytostatics. 

The Balb/3T3 and UM-UC-3 were cultured in Dulbecco’s Modified Eagle Medium (DMEM; Life Technologies, UK) supplemented with 10% (*v*/*v*) fetal bovine serum (FBS; GE Healthcare HyClone, Logan, USA) and 2 mM L-glutamine (Sigma-Aldrich, Poznań, Poland). The UM-UC-3^CDDP^, UM-UC-3^GEM^, UM-UC-^3VBL^, and UM-UC-3^CDDP/GEM^ culture media were additionally supplemented with appropriate cytostatics: 2.5 µg/mL cisplatin (Accord, Warsaw, Poland) for UM-UC-3^CDDP^, 500 nM gemcitabine (Sigma-Aldrich, Poznań, Poland) for UM-UC-3^GEM^, 5 nM vinblastine (Sigma-Aldrich, Poznań, Poland) for UM-UC-3VBL, 2.5 µg/mL cisplatin and 500 nM gemcitabine for UM-UC-3^CDDP/GEM^. The LoVo cell line was cultured in F-12K Nutrient Mixture (F-12K; Corning, Corning, USA), supplemented with 10% (*v*/*v*) FBS. LoVo^Dx^ culture medium was additionally supplemented with doxorubicin 100 ng/mL (Accord, Warsaw, Poland). The MCF-7 cell line was cultured in Eagle’s medium (HIIET, PAS, Wroclaw, Poland), supplemented with 10% (*v*/*v*) FBS (Sigma-Aldrich, Poznań, Poland), 2 mM L-glutamine, MEM non-essential amino acid solution 1% (*v*/*v*) (Sigma-Aldrich, Poznań, Poland), insulin 8 µg/mL (Sigma-Aldrich, Poznań, Poland). The MDA-MB-231 cell line was cultured in RPMI 1640 (HIIET, PAS, Wroclaw, Poland), supplemented with 10% (*v*/*v*) FBS (Sigma-Aldrich, Poznań, Poland) and 2 mM L-glutamine. The A 549 cell line was cultured in OptiMEM (HIIET, PAS, Wroclaw, Poland), supplemented with 5% (*v*/*v*) FBS (GE Healthcare HyClone, Logan, USA) and 2 mM L-glutamine. All culture media were supplemented with antibiotics—100 μg/mL streptomycin (Polfa Tarchomin, Warsaw, Poland) and 100 U/mL penicillin (Sigma-Aldrich, Poznań, Poland). 

All cell lines were cultured in a humidified atmosphere at 37 °C with 5% (*v*/*v*) CO_2_ and passaged twice a week using EDTA-Trypsin solution (pH 8; HIIET, Wroclaw, Poland) as a detachment agent.

All compounds, for antiproliferative activity assessment, were stored at 80 °C as 50 mM solutions in dimethyl sulfoxide (DMSO; Avantor Performance Materials, Gliwice, Poland).

#### 3.6.2. Antiproliferative Activity Assessment by Sulforhodamine B Assay

The cells were seeded on 384-well plates (Greiner Bio One, Kremsmünster, Austria) at 1 × 10^3^ cells/well density for A 549, UM-UC-3, UM-UC-3^CDDP^, UM-UC-3^GEM^, UM-UC-3^CVBL^ and UM-UC-3^CDDP/GEM^ cell lines. Other cell lines were seeded at 2 × 10^3^ cells/well. After overnight incubation, compounds were applied at eight concentrations (ranging from 100 µM to 0.03 µM). After 72 h incubation, the sulforhodamine B (SRB) assay based on Skehan et al. [41] was carried out with slight modifications. In brief, 50 µL of the medium was replaced with 30 µL/well of 25% (*w*/*v*) trichloroacetic acid (Avantor Performance Materials, Gliwice, Poland). After 1 h incubation at room temperature, plates were washed three times with tap water, and 20 µL of 0.1% (*w*/*v*) solution of sulforhodamine B (Sigma-Aldrich, Poznan, Poland) in 1% (*v*/*v*) acetic acid (Avantor Performance Materials, Gliwice, Poland) was added to each well. After 30 min incubation at room temperature, the unbound dye was washed out with 1% (*v*/*v*) acetic acid. Bound dye was solubilized with 70 µL of 10 mM unbuffered TRIS (Avantor Performance Materials, Gliwice, Poland) solution. The procedure was performed using Biotek EL-406 washing station (BioTek Instruments, USA). Absorbance was read using a Biotek Hybrid H4 reader (BioTek Instruments, USA) at 540 nm wavelength. Crude absorbance data was used to calculate proliferation inhibition using the formula (1). Then it was used for IC_50_ calculations performed in (GraphPad Software, Inc.) utilizing ‘[Inhibitor] vs. response—Variable slope (four parameters)’ model.

(1) $$\%Inh\;=\;\left[\left(\frac{A_{p}-A_{m}}{A_{k}-A_{m}}\right)\times 100\right]-100$$

*A_m_*—absorbance for cell-free wells, *A_k_*—absorbance for vehicle-treated, control wells, *A_p_*—absorbance for compounds-treated wells

## 4. Conclusions

An efficient procedure for the synthesis of dehydropeptides containing structurally variable (*Z*)-dehydrocysteines was elaborated by applying readily available peptides containing (*Z*)-β-bromo-dehydroalanine as substrates. These peptides might be considered as building blocks for the synthesis of artificial enzymes. Peptides containing triazole in the side chain appeared to be novel complexones of Cu^2+^ ions. Additionally, the dipeptides **6** and **7** exhibited potent antiproliferative properties against cancer cell lines, including lines resistant to the action of known anticancer agents.

## Figures and Tables

**Table 1:** The yield of synthesised compounds.

Compound No.	Name Abbreviation	Isolated Yield [%]
**6a**	Boc-Gly-ΔCys(*S*-triazole)-OMe	93
**7a**	Boc-Phe-ΔCys(*S*-triazole)-OMe	94
**6b**	Boc-Gly-ΔCys(*S*-4-phenylimidazole)-OMe	93
**7b**	Boc-Phe-ΔCys(*S*-4-phenylimidazole)-OMe	86
**6c**	Boc-Gly-ΔCys(*S*-5-methoxybenzoxazole)-OMe	90
**7c**	Boc-Phe-ΔCys(*S*-5-methoxybenzoxazole)-OMe	76
**6d**	Boc-Gly-ΔCys(*S*-benzothiazole)-OMe	82
**7d**	Boc-Phe-ΔCys(*S*-benzothiazole)-OMe	92
**6e**	Boc-Gly-ΔCys(*S*-adenine)-OMe	61
**7e**	Boc-Phe-ΔCys(*S*-adenine)-OMe	52
**8**	Boc-Gly-ΔCys(*S,S*-bismethylbipyridine)-OMe	80
**9a**	HCl Gly-ΔCys(*S*-triazole)-OMe	[a]
**10a**	HCl Phe-ΔCys(*S*-triazole)-OMe	[a]
**9d**	HCl Gly-ΔCys(*S*-benzothiazole)-OMe	[a]
**10d**	HCl Phe-ΔCys(*S*-benzothiazole)-OMe	[a]

[a] The product obtained quantitatively.

**Table 2:** The protonation constants of free ligands and stability constants of formed Cu(II)-complexes. The errors are represented by ± SD.

	9a		10a	
Form	log*β*	log*K*	log*β*	log*K*
	*The protonation constants of free ligands*			
H_3_L	25.31 ± 0.01	7.26	25.38 ± 0.02	6.85
H_2_L	18.05 ± 0.01	7.96 (-NH_2_)	18.53 ± 0.02	8.11 (-NH_2_)
HL	10.09 ± 0.01	10.09	10.42 ± 0.01	10.42
	*The stability constants of Cu(II)-complexes*			
CuLH_2_	23.52 ± 0.03	9.34	23.83 ± 0.03	8.83
CuL	14.18 ± 0.01	10.42	15.00 ± 0.01	10.61
CuLH_-1_	3.76 ± 0.04	-	4.39 ± 0.03	-
Cu_2_L_2_H	36.61 ± 0.03	14.45	37.54 ± 0.06	14.59
Cu_2_L_2_H_-1_	22.16 ± 0.06	-	22.95 ± 0.06	-
CuL_2_H_2_	36.46 ± 0.04	7.67	-	-
CuL_2_H	28.79 ± 0.04	8.73	28.82 ± 0.11	8.45
CuL_2_	20.06 ± 0.04	11.35	20.37 ± 0.05	-
CuL_2_H_-1_	8.71 ± 0.07	-	-	-

**Table 3:** Antiproliferative activity of compounds **6**, **7**, **9** and **10** on five human cancer cell lines and non-tumorigenic line Balb/3T3 given as IC_50_ ± SD (µM), with cisplatin (CDDP) used as reference.

Cpd.	MCF-7	MDA-MB-231	A-549	LoVo	LoVo^DX^	Balb/3T3
**6b**	31.8 ± 7.8	35.0 ± 10.0	27.2 ± 7.7	32.4 ± 2.6	[a]	38.4 ± 3.5
**6c**	5.3 ± 0.9	5.0 ± 0.8	6.2 ± 1.4	8.7 ± 1.1	6.4 ± 0.4	5.3 ± 1.2
**6d**	4.5 ± 0.5	6.1 ± 0.6	5.6 ± 0.9	6.7 ± 0.7	6.2 ± 0.6	5.8 ± 0.9
**7a**	74.5 ± 5.8	53.3 ± 5.5	60.7 ± 12.1	75.8 ± 6.5	[a]	19.7 ± 1.9
**7b**	15.5 ± 2.5	15.2 ± 4.4	12.0 ± 4.0	16.4 ± 1.7	23.0 ± 2.2	7.6 ± 0.9
**7c**	4.5 ± 0.4	7.1 ± 0.8	4.0 ± 1.1	6.3 ± 0.5	4.5 ± 0.4	5.3 ± 0.4
**7d**	6.8 ± 0.5	10.8 ± 0.4	6.6 ± 1.4	9.6 ± 0.9	8.6 ± 0.9	3.1 ± 0.6
**7e**	100.8 ± 11.3	74.5 ± 7.7	[a]	81.7 ± 12.4	[a]	[a]
**9d**	[a]	56.0 ± 7.1	33.4 ± 6.4	[b]	[b]	[b]
**10d**	22.0 ± 4.5	24.4 ± 3.0	14.0 ± 5.6	70.3 ± 21.5	33.1 ± 9.0	42.2 ± 9.3
CDDP	6.0 ± 2.2	24.4 ± 4.8	2.7 ± 1.0	4.4 ± 0.3	3.4 ± 0.2	10.2 ± 4.8

[a] IC_50_ exceeds highest concentration used (100µM), [b] not tested.

**Table 4:** Antiproliferative activity of studied compounds on urinary bladder transitional cell carcinoma cell line (UM-UC-3) and its variants resistant to cisplatin (CDDP), gemcitabine (GEM), both gemcitabine and cisplatin(CDDP/GEM) and vinblastine (VBL). Results are given as IC_50_ ± SD (µM), with cisplatin (CDDP) used as reference.

Cpd.	UM-UC-3	UM-UC-3^CDDP^	UM-UC-3^GEM^	UM-UC-3^CDDP/GEM^	UM-UC-3^VBL^
**6b**	23.1 ± 2.6	34.6 ± 3.6	35.1 ± 2.8	32.5 ± 4.0	33.8 ± 2.6
**6c**	7.2 ± 0.6	7.8 ± 0.7	5.2 ± 0.6	8.6 ± 1.4	6.0 ± 1.5
**6d**	4.6 ± 0.4	6.4 ± 0.4	4.6 ± 0.5	6.2 ± 0.8	4.9 ± 0.8
**7a**	56.0 ± 5.7	67.7 ± 3.9	65.2 ± 4.9	62.2 ± 5.3	54.9 ± 3.5
**7b**	13.5 ± 4.5	19.0 ± 3.2	19.9 ± 3.3	17.2 ± 2.1	17.3 ± 1.7
**7c**	6.2 ± 0.8	6.8 ± 0.8	7.0 ± 0.5	5.9 ± 0.4	5.8 ± 0.6
**7d**	6.7 ± 1.4	11.2 ± 1.5	8.0 ± 2.3	9.1 ± 1.2	6.3 ± 1.0
**10d**	30.5 ± 3.7	16.6 ± 2.5	67.8 ± 9.6	25.3 ± 8.0	9.4 ± 2.2
CDDP	2.4 ± 0.6	26.1 ± 1.8	2.6 ± 0.2	25.1 ± 1.9	2.3 ± 0.3

## Data Availability

All data are reported in the manuscript and in the Supplementary Material and are available from corresponding author upon request.

## Supplementary Materials

The following are available online at https://www.mdpi.com/1422-0067/22/4/2168/s1, General Information; Discussion of NMR analysis; Figure S1: ^13^C NMR spectra for dipeptide **6e** containing adenine residue performed with different experimental time; Figure S2: NOE difference NMR spectra for dipeptides **6a** and **7a**; Characterization of compounds: **6a–e**, **7a–e**, **8**, **9a**, **9d**, **10a** and **10d**; ^1^H NMR, ^13^C NMR and IR spectra of the obtained compounds.
