# Supplementary material for: Dipeptides of S-Substituted Dehydrocysteine as Artzyme Building Blocks: Synthesis, Complexing Abilities and Antiproliferative Properties
Source: Int J Mol Sci. 2021 Feb 22;22(4):2168. doi: 10.3390/ijms22042168 (PMC7927096; doi:10.3390/ijms22042168)
Supplement: Supplementary file 1 [file ijms-22-02168-s001.pdf]

Supplementary Materials

Dipeptides of *S*-substituted dehydrocysteine as artzyme building blocks: synthesis, complexing abilities and antiproliferative properties

Paweł Lenartowicz,*[a] Mateusz Psurski,[b] Aleksandra Kotynia,[c] Aleksandra Pieniężna,[c] Monika Cuprych,[b] Klaudia Poniatowska,[b] Justyna Brasuń,[c] and Paweł Kafarski*[d]

E-mail: pawel.lenartowicz@uni.opole.pl (P.L); pawel.kafarski@pwr.edu.pl (P.K.)

Table of contents

1. General information 2
2. Discussion of NMR analysis 2
3. Characterization of compounds 4
4. ^{1}H NMR, ^{13}C NMR and IR spectra 9

1. General information

All thiols (1H-1,2,4-triazole-3-thiol 97%, 4-phenylimidazole-2-thiol 97%, 5-methoxybenzoxazole-2-thiol 97%, 2-mercaptobenzothiazole 99%, 8-mercaptoadenine 95%, 5,5'-bis(mercaptomethyl)-2,2'-bipyridine 96%) were purchased from Merck (Sigma Poland). The solvents: acetonitrile (ACN), dichloromethane (DCM), chloroform (CHL), ethyl acetate (EtOAc) were refluxed over P_2O_5 and distilled. The reaction progress was monitored by thin layer chromatography on Merck 60 silica plates with fluorescent indicator. The spots were additionally visualized with potassium permanganate solution or by chlorine/*o*-tolidine reaction. The compounds were purified by flash chromatography using silica gel 60 (0.040-0.063 mm) from Merck. The NMR analyses were performed on a Bruker Ultrashield 400 MHz spectrometer operating at 400 MHz (^{1}H) and 101 MHz (^{13}C). The samples were prepared in d_6-DMSO (99.8 at. %D) containing 0.03% TMS and measured at 297 K. High-resolution mass spectra (HRMS) were recorded on a Waters LCT Premier XE mass spectrometer equipped with an ESI source in the positive ion mode (Waters, Milford, MA, USA). IR spectra were recorded on Nicolet 6700 FT-IR spectrophotometer (Thermo Scientific) operating at resolution 2 cm^{-1} and scanning range 4000 – 400 cm^{-1}. Samples were measured as KBr disks.

2. Discussion of NMR analysis

The structures of obtained compounds were confirmed by ^{1}H and ^{13}C NMR analysis. However, the ^{13}C NMR spectra of compound **6a**, **6e**, **7a** and **7e** recorded upon standard time (up to 1 hour) revealed that some carbon signals are missing. The extension of experiment time to 15 hours for compound **7a** and **7e** let to recording spectra, in which missed signals derived from *S*-substituted dehydrocysteine fragment appeared as broad peaks with low intensity (Fig. SI1). This observation could be explained by presence in the structure a large number of nitrogen atoms in aromatic fragment which affect the relaxation time.

The addition-elimination reaction of thiols to β-bromodehydroalaniates could produce two geometrical isomers. The determination of *Z, E* configuration of two representative compounds **6a** and **7a** was performed by NOE difference NMR experiment (Fig SI2). The irradiation of amide proton of **6a** (9.5 ppm) gave three positive signals derived from protons connected to methylene group of glycine, urethane and *tert*-butyl groups. In the case of **7a**, irradiation of amide proton gave only one positive peak derived from protons of *tert*-butyl groups. In the second experiment, in which vinyl proton of dehydrocysteine (8.00 ppm) was irradiated, two positive peaks derived from protons of *tert*-butyl and methyl groups were observed for both products. Moreover, all NMR experiments showed that there is no interaction between amide and vinyl proton. Thus, the configuration of prepared compounds was determined as *Z*. This is in a good accordance with our expectation because *Z* dehyropeptides are more thermodynamically favorable than *E* isomers.

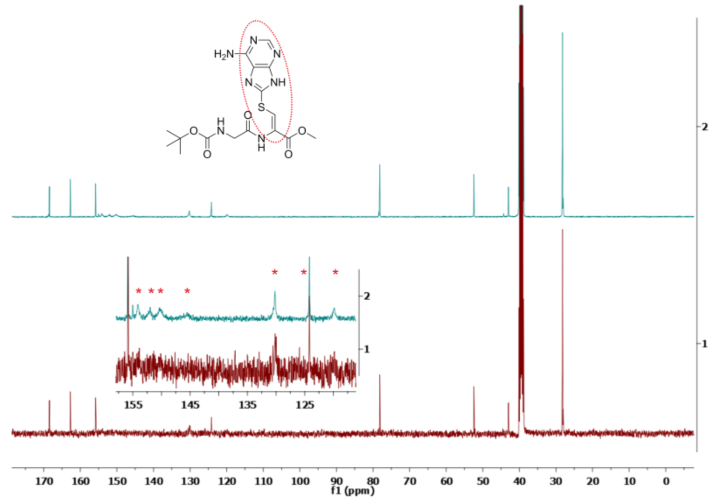

Figure S1 ^{13}C NMR spectra for dipeptide **6e** containing adenine residue performed with different experimental time: 1 – one hour; 2 – fifteen hours. The signals derived from carbon atoms of *S*-substituted dehydrocysteine fragment are marked by asterisks.

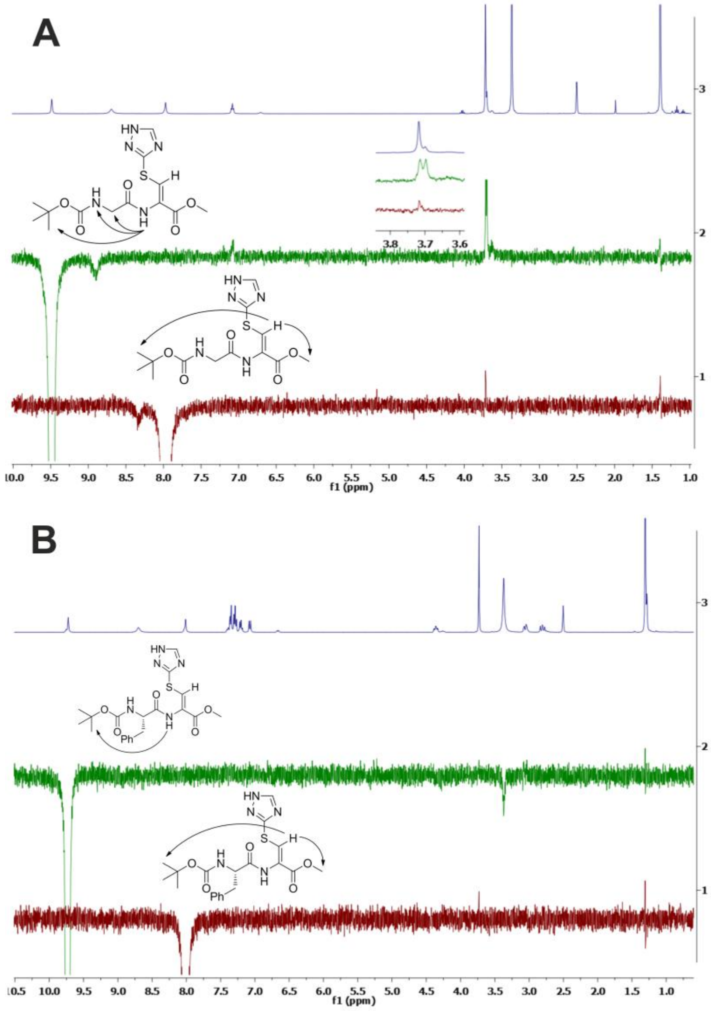

Figure S2 NOE difference NMR spectra for dipeptides **6a** (panel A) and **7a** (panel B). The range of ppm axis is set up for better readability at 0.5-10.5 ppm. The signal derived from $NH_{triazole}$ (14.4 ppm) is omitted.

3. Characterization of compounds

Boc-Gly-ΔCys(*S*-triazole)-OMe (6a)

Synthesis was performed using dehyropeptide (0.337 g, 1 mmol), 1H-1,2,4-triazole-3-thiol (0.110 g, 1.05 mmol), K_2CO_3 (0.276 g, 2 mmol), ACN (10 ml). The product (0.331 g, 0.93 mol) was obtained as white solid with yield 93%.

Flash chromatography: increasing gradient of A (10% MeOH in EtOAc) in B (CHL) (0→100%). TLC: 5% MeOH/45% EtOAc/50% CHL, R_f = 0.20.

^{1}H NMR (400 MHz, DMSO) δ 14.41 (s, 1H), 9.48 (s, 1H), 8.70 (s, 1H), 7.98 (s, 1H), 7.08 (t, *J* = 5.7 Hz, 1H), 3.71 (d and s overlapped, 5H), 1.39 ppm (s, 9H).
^{13}C NMR (101 MHz, DMSO) δ 168.32, 162.69, 155.84, 145.52, 131.40, 123.64, 78.14, 52.40, 43.00, 28.23 ppm.
IR ν (KBr disk, cm^{-1}): 3378, 3275, 2979, 2933, 1716, 1695, 1511, 1438, 1368, 1328, 1279, 1249, 1165
HRMS (ESI): *m/z* calcd for $C_{13}H_{19}N_5O_5S+H^+$: 358.1180 [*M*+H]$^+$; found: 358.1190.

Boc-Gly-ΔCys(*S*-4-phenylimidazole)-OMe (6b)

Synthesis was performed using dehyropeptide (0.337 g, 1 mmol), 4-phenylimidazole-2-thiol (0.191 g, 1.05 mmol), K_2CO_3 (0.276 g, 2 mmol), ACN (10 ml). The product (0.402 g, 0.93 mol) was obtained as white solid with yield 93%.

Flash chromatography: increasing gradient of EtOAc in CHL (0→100%). TLC: 60% EtOAc in CHL, R_f = 0.32.

^{1}H NMR (400 MHz, DMSO) δ 12.85 (s, 1H), 9.50 (s, 1H), 7.91 – 7.31 (m and s overlapped, 6H), 7.31 – 7.16 (m, 1H), 7.09 (t, *J* = 6.1 Hz, 1H), 3.72 (d, *J* = 6.1 Hz, 2H), 3.71 (s, 3H), 1.40 ppm (s, 9H).
^{13}C NMR (101 MHz, DMSO) δ 168.33, 162.73, 155.85, 141.97, 136.28, 133.91, 132.93, 128.60, 126.59, 124.30, 123.22, 116.06, 78.15, 52.38, 42.98, 28.25 ppm.
IR ν (KBr disk, cm^{-1}): 3381, 3188, 2978, 1721, 1687, 1497, 1450, 1438, 1368, 1323, 1247, 1167, 763.
HRMS (ESI): *m/z* calcd for $C_{20}H_{24}N_4O_5S+H^+$: 433.1540 [*M*+H]$^+$; found: 433.1541.

Boc-Gly-ΔCys(*S*-5-methoxybenzoxazole)-OMe (6c)

Synthesis was performed using dehyropeptide (0.337 g, 1 mmol), 5-methoxybenzoxazole-2-thiol (0.196 g, 1.05 mmol), K_2CO_3 (0.276 g, 2 mmol), ACN (10 ml). The product (0.394 g, 0.90 mol) was obtained as white solid with yield 90%.

Flash chromatography: increasing gradient of EtOAc in CHL (0→50%). TLC: 50% EtOAc in CHL, R_f = 0.38.

^{1}H NMR (400 MHz, DMSO) δ 9.80 (s, 1H), 7.91 (s, 1H), 7.62 (d, *J* = 8.9 Hz, 1H), 7.33 (d, *J* = 2.5 Hz, 1H), 7.15 (t, *J* = 6.1 Hz, 1H), 6.95 (dd, *J* = 8.9, 2.5 Hz, 1H), 3.80 (s, 3H), 3.79 (s, 3H), 3.75 (d, *J* = 6.1 Hz, 2H), 1.40 ppm (s, 9H).
^{13}C NMR (101 MHz, DMSO) δ 168.71, 162.43, 160.88, 157.24, 155.89, 146.01, 141.79, 126.58, 124.23, 112.85, 110.86, 102.53, 78.22, 55.87, 52.85, 43.14, 28.21 ppm.
IR ν (KBr disk, cm^{-1}): 3321, 3253, 2979, 1720, 1679, 1532, 1505, 1484, 1437, 1321, 1289, 1249, 1155, 750.
HRMS (ESI): *m/z* calcd for $C_{19}H_{23}N_3O_7S+H^+$: 438.1329 [*M*+H]$^+$; found: 438.1339.

Boc-Gly-ΔCys(*S*-benzothiazole)-OMe (6d)

Synthesis was performed using dehyropeptide (0.169 g, 0.5 mmol), 2-mercaptobenzothiazole (0.090 g, 525 mmol), K_2CO_3 (0.138 g, 1 mmol), ACN (6 ml). The product (0.175 g, 0.41 mol) was obtained as white solid with yield 82%.

Flash chromatography: increasing gradient of EtOAc in DCM (0→40%). TLC: 30% EtOAc in DCM, R_f = 0.42.

^{1}H NMR (400 MHz, DMSO) δ 9.76 (s, 1H), 8.10 (d, *J* = 7.4 Hz, 1H), 8.02 (s, 1H), 7.99 (d, *J* = 7.9 Hz, 1H), 7.57 – 7.50 (m, 1H), 7.48 – 7.40 (m, 1H), 7.13 (t, *J* = 6.1 Hz, 1H), 3.78 (s, 3H), 3.74 (d, *J* = 6.1 Hz, 2H), 1.40 ppm (s, 9H).
^{13}C NMR (101 MHz, DMSO) δ 168.55, 162.62, 162.46, 155.87, 152.36, 134.97, 126.86, 126.14, 125.91, 125.30, 122.30, 121.90, 78.21, 52.79, 43.12, 28.23 ppm.
IR ν (KBr disk, cm^{-1}): 3333, 3270, 2979, 1722, 1681, 1529, 1463, 1427, 1368, 1318, 1274, 1172, 994, 757.
HRMS (ESI): *m/z* calcd for $C_{18}H_{21}N_3O_5S_2+H^+$: 424.0995 [*M*+H]$^+$; found: 424.1010.

Boc-Gly-ΔCys(*S*-adenine)-OMe (6e)

Synthesis was performed using dehyropeptide (0.337 g, 1 mmol), 8-mercaptoadenine (0.185 g, 1.05 mmol), K_2CO_3 (0.276 g, 2 mmol), ACN (10 ml) and DMF extra dry (1 ml). In order to remove DMF the residue remained after solvent evaporation was dissolved in EtOAc and washed with brine and dried with anhydrous $MgSO_4$. Then chromatographic purification was performed. The product (0.260 g, 0.61 mol) was obtained as white solid with yield 61%.

Flash chromatography: increasing gradient of MeOH in DCM (0→20%). TLC: 10% MeOH in DCM, R_f = 0.23.

^{1}H NMR (400 MHz, DMSO) δ 13.49 (s, 1H), 9.56 (s, 1H), 8.11 (2 x s overlapped, 2H), 7.36 (s, 2H), 7.10 (t, *J* = 6.1 Hz, 1H), 3.77 – 3.63 (s and d overlapped, 5H), 1.39 ppm (s, 9H).
^{13}C NMR (101 MHz, DMSO, measurement time 15 h) δ 168.48, 162.76, 155.87, 154.21 broad, 152.02 broad, 150.38 broad, 145.62 broad, 130.22 broad, 124.22, 119.89 broad, 78.19, 52.47, 43.02, 28.26 ppm.
IR ν (KBr disk, cm^{-1}): 3496, 3399, 3362, 3204, 2977, 1723, 1659, 1603, 1564, 1495, 1436, 1367, 1322, 1251, 1168
HRMS (ESI): *m/z* calcd for $C_{16}H_{21}N_7O_5S+H^+$: 424.1398 [*M*+H]$^+$; found: 424.1397.

Boc-Phe-ΔCys(*S*-triazole)-OMe (7a)

Synthesis was performed using dehyropeptide (0.427 g, 1 mmol), 1H-1,2,4-triazole-3-thiol (0.110 g, 1.05 mmol), K_2CO_3 (0.276 g, 2 mmol), ACN (10 ml). The product (0.419 g, 0.94 mol) was obtained as white solid with yield 94%.

Flash chromatography: increasing gradient of MeOH in DCM (0→5%). TLC: 5% MeOH in DCM, R_f = 0.30.

^{1}H NMR (400 MHz, DMSO) δ 14.42 (s, 1H), 9.72 (s, 1H), 8.70 (s, 1H), 8.01 (s, 1H), 7.42 – 7.16 (m, 5H), 7.07 (d, *J* = 8.7 Hz, 1H), 4.40 – 4.31 (m, 1H), 3.73 (s, 3H), 3.05 (dd, *J* = 13.7, 3.7 Hz, 1H), 2.80 (dd, *J* = 13.7, 11.0 Hz, 1H), 1.30 ppm (s, 3H).
^{13}C NMR (101 MHz, DMSO) δ 170.90, 162.74, 155.39, 145.52 broad, 138.11, 131.45 broad, 129.33, 128.08, 126.30, 123.68 broad, 78.09, 55.83, 52.42, 37.46, 28.18 ppm.
IR ν (KBr disk, cm^{-1}): 3319, 2979, 2931, 1690, 1669, 1517, 1498, 1438, 1367, 1329, 1278, 1248, 1165
HRMS (ESI): *m/z* calcd for $C_{20}H_{25}N_5O_5S+H^+$: 448.1649 [*M*+H]$^+$; found: 448.1656.

Boc-Phe-ΔCys(*S*-4-phenylimidazole)-OMe (7b)

Synthesis was performed using dehyropeptide (0.214 g, 0.5 mmol), 4-phenylimidazole-2-thiol (0.095 g, 0.525 mmol), K_2CO_3 (0.138 g, 1 mmol), ACN (6 ml). The product (0.227 g, 0.43 mol) was obtained as white solid with yield 86%.

Flash chromatography: increasing gradient of EtOAc in DCM (0→50%). TLC: 20% EtOAc in DCM, R_f = 0.24.

^{1}H NMR (400 MHz, DMSO) δ 12.86 (s, 1H), 9.74 (s, 1H), 7.83 – 7.18 (m set overlapped, 12H), 7.08 (d, *J* = 8.8 Hz, 1H), 4.44 – 4.33 (m, 1H), 3.72 (s, 3H), 3.08 (dd, *J* = 13.7, 3.3 Hz, 1H), 2.81 (dd, *J* = 13.7, 11.2 Hz, 1H), 1.31 ppm (s, 9H).
^{13}C NMR (101 MHz, DMSO) δ 170.98, 162.76, 155.38, 141.96, 138.12, 136.29, 133.93, 133.09, 129.33, 128.60, 128.09, 126.59, 126.31, 124.33, 123.27, 116.06, 78.09, 55.83, 52.40, 37.56, 28.19 ppm.
IR ν (KBr disk, cm^{-1}): 3373, 3269, 3063, 3030, 2978, 1718, 1692, 1606, 1496, 1454, 1437, 1393, 1367, 1323, 1248, 1166, 759, 697.
HRMS (ESI): *m/z* calcd for $C_{27}H_{30}N_4O_5S+H^+$: 523.2010 $[M+H]^+$; found: 523.2012.

Boc-Phe-ΔCys(*S*-5-methoxybenzoxazole)-OMe (7c)

Synthesis was performed using dehyropeptide (0.214 g, 0.5 mmol), 5-methoxybenzoxazole-2-thiol (0.098 g, 0.525 mmol), K_2CO_3 (0.138 g, 1 mmol), ACN (8 ml). The product (0.199 g, 0.38 mol) was obtained as white solid with yield 76%.

Flash chromatography: increasing gradient of EtOAc in heksane (0→80%). TLC: 40% EtOAc in hexane, R_f = 0.28.

^{1}H NMR (400 MHz, DMSO) δ 10.03 (s, 1H), 7.94 (s, 1H), 7.63 (d, *J* = 9.0 Hz, 1H), 7.42 – 7.13 (m, 7H), 6.95 (dd, *J* = 9.0, 2.6 Hz, 1H), 4.45 – 4.35 (m, 1H), 3.80 (s, 6H), 3.05 (dd, *J* = 13.6, 3.8 Hz, 1H), 2.82 (dd, *J* = 13.6, 11.0 Hz, 1H), 1.32 ppm (s, 9H).
^{13}C NMR (101 MHz, DMSO) δ 171.25, 162.46, 160.89, 157.24, 155.48, 146.03, 141.80, 137.94, 129.34, 128.10, 126.55, 126.36, 124.58, 112.87, 110.86, 102.53, 78.20, 55.95, 55.87, 52.88, 37.20, 28.17 ppm.
IR ν (KBr disk, cm^{-1}): 3384, 3362, 2971, 1718, 1700, 1612, 1508, 1482, 1466, 1438, 1324, 1244, 1157, 1023, 756, 701
HRMS (ESI): *m/z* calcd for $C_{26}H_{29}N_3O_7S+H^+$: 528.1799 $[M+H]^+$; found: 528.1803.

Boc-Phe-ΔCys(*S*-benzothiazole)-OMe (7d)

Synthesis was performed using dehyropeptide (0.214 g, 0.5 mmol), 2-mercaptobenzothiazole (0.090 g, 0.525 mmol), K_2CO_3 (0.138 g, 1 mmol), ACN (8 ml). The product (0.234 g, 0.46 mol) was obtained as white solid with yield 92%.

Flash chromatography: increasing gradient of EtOAc in heksane (0→50%). TLC: 40% EtOAc in hexane, R_f = 0.36.

^{1}H NMR (400 MHz, DMSO) δ 9.97 (s, 1H), 8.10 (d, *J* = 7.9 Hz, 1H), 8.07 (s, 1H), 8.00 (d, *J* = 8.0 Hz, 1H), 7.57 – 7.50 (m, 1H), 7.48 – 7.42 (m, 1H), 7.41 – 7.18 (m, 5H), 7.15 (d, *J* = 8.6 Hz, 1H), 4.44 – 4.34 (m, 1H), 3.79 (s, 3H), 3.06 (dd, *J* = 13.6, 3.8 Hz, 1H), 2.83 (dd, *J* = 13.6, 11.0 Hz, 1H), 1.32 ppm (s, 9H).
^{13}C NMR (101 MHz, DMSO) δ 171.09, 162.64, 162.44, 155.44, 152.36, 137.97, 134.95, 129.36, 128.09, 126.87, 126.54, 126.34, 125.85, 125.30, 122.30, 121.90, 78.18, 55.90, 52.79, 37.28, 28.18 ppm;
IR ν (KBr disk, cm^{-1}): 3373, 3064, 2977, 2929, 1720, 1690, 1606, 1498, 1456, 1428, 1367, 1323, 1247, 1167, 994, 756, 701
HRMS (ESI): *m/z* calcd for $C_{25}H_{27}N_3O_5S_2+H^+$: 514.1465 $[M+H]^+$; found: 514.1475.

Boc-Phe-ΔCys(*S*-adenine)-OMe (7e)

Synthesis was performed using dehyropeptide (0.214 g, 0.5 mmol), 8-mercaptoadenine (0.092 g, 0.525 mmol), K_2CO_3 (0.138 g, 1 mmol), ACN (10 ml) and DMF extra dry (1 ml). In order to remove DMF the residue remained after solvent evaporation was dissolved in EtOAc and washed with brine and dried with anhydrous $MgSO_4$. Then chromatographic purification was performed. The product (0.132 g, 0.26 mol) was obtained as white solid with yield 52%.

Flash chromatography: increasing gradient of MeOH in DCM (0→15%). TLC: 10% MeOH in DCM, R_f = 0.37.

^{1}H NMR (400 MHz, DMSO) δ 13.53 (s, 1H), 9.79 (s, 1H), 8.15 (s, 1H), 8.12 (s, 1H), 7.47 – 7.15 (m, 7H), 7.09 (d, *J* = 8.7 Hz, 1H), 4.43 – 4.33 (m, 1H), 3.74 (s, 3H), 3.07 (dd, *J* = 13.6, 3.4 Hz, 1H), 2.81 (dd, *J* = 13.6, 11.2 Hz, 1H), 1.30 ppm (s, 9H).
^{13}C NMR (101 MHz, DMSO) δ 171.10, 162.79, 155.40, 154.29 broad, 151.87 broad, 150.42 broad, 145.59 broad, 138.09, 130.42 broad, 129.34, 128.11, 126.34, 124.26, 120.03 broad, 78.13, 55.85, 52.47, 37.50, 28.20 ppm.
IR ν (KBr disk, cm^{-1}): 3333, 3191, 3067, 2978, 1716, 1662, 1604, 1497, 1455, 1438, 1367, 1320, 1249, 1168, 700.
HRMS (ESI): *m/z* calcd for $C_{23}H_{27}N_7O_5S+H^+$: 514.1867 [*M*+H]$^+$; found: 514.1869.

Boc-Gly-ΔCys(*S,S*-bismethylbipyridine)-OMe (8)

Synthesis was performed using dehyropeptide (0.337 g, 1 mmol), 5,5′-bis(mercaptomethyl)-2,2′-bipyridine (0.129 g, 0.5 mmol), K_2CO_3 (0.276 g, 2 mmol), ACN (10 ml). The product (0.301 g, 0.40 mol) was obtained as white solid with yield 80%.

Flash chromatography: increasing gradient of MeOH in CHL (0→10%). TLC: 10% MeOH in CHL, R_f = 0.37.

^{1}H NMR (400 MHz, DMSO) δ 9.15 (s, 1H), 8.69 (d, *J* = 1.8 Hz, 1H), 8.35 (d, *J* = 8.2 Hz, 1H), 7.93 (dd, *J* = 8.2, 1.8 Hz, 1H), 7.59 (s, 1H), 6.97 (t, *J* = 6.1 Hz, 1H), 4.29 (s, 2H), 3.63 (s and d overlapped, 5H), 1.36 ppm (s, 9H).
^{13}C NMR (101 MHz, DMSO) δ 168.12, 162.80, 155.75, 153.99, 149.43, 137.95, 137.61, 134.84, 122.26, 120.43, 78.03, 52.00, 42.79, 33.55, 28.21 ppm.
IR ν (KBr disk, cm^{-1}): 3390, 3300, 3004, 2978, 2953, 1713, 1665, 1596, 1520, 1468, 1437, 1367, 1332, 1246, 1168, 847, 750
HRMS (ESI): *m/z* calcd for $C_{34}H_{44}N_6O_{10}S_2+H^+$: 761.2633 [*M*+H]$^+$; found: 761.2635.

HCl·Gly-ΔCys(*S*-triazole)-OMe (9a)

The peptide (0.098 g, 0.27 mmol) was treated with HCl in methanol (1.5 M, 3 ml). The product (0.082 g) was obtained quantitatively.

^{1}H NMR (400 MHz, DMSO) δ 10.23 (s, 1H), 8.69 (s, 1H), 8.30 (s, 3H), 8.08 (s, 1H), 3.81 – 3.75 (m, 2H), 3.73 ppm (s, 3H).
^{13}C NMR (101 MHz, DMSO) δ 165.10, 162.37, 154.83, 145.95, 132.68, 122.87, 52.60, 40.27.
IR ν (KBr disk, cm^{-1}): 3409, 3182, 3121, 3017, 2952, 1714, 1690, 1611, 1560, 1510, 1477, 1432, 1329, 1270, 1251, 1095, 969, 849, 751.
HRMS (ESI): *m/z* calcd for $C_8H_{11}N_5O_3S+H^+$: 258.0655 [*M*+H]$^+$; found: 258.0668.

HCl·Gly-ΔCys(*S*-benzothiazole)-OMe (9d)

The peptide (0.085 g, 0.20 mmol) was treated with HCl in methanol (1.5 M, 3 ml). The product (0.070 g) was obtained quantitatively.

^{1}H NMR (400 MHz, DMSO) δ 10.50 (s, 1H), 8.30 (s, 3H), 8.12 (s and d overlapped, 2H), 8.00 (d, *J* = 7.9 Hz, 1H), 7.61 – 7.51 (m, 1H), 7.49 – 7.42 (m, 1H), 3.84 – 3.78 ppm (m and s overlapped, 5H).
^{13}C NMR (101 MHz, DMSO) δ 165.31, 162.26, 161.82, 152.32, 135.02, 127.78, 126.95, 125.44, 125.11, 122.37, 121.99, 52.92, 40.37.
IR ν (KBr disk, cm^{-1}): 3430, 3065, 2994, 2952, 1704, 1608, 1529, 1462, 1426, 1327, 1311, 1240, 996, 757
HRMS (ESI): *m/z* calcd for $C_{13}H_{13}N_3O_3S_2+H^+$: 324.0471 [*M*+H]$^+$; found: 324.0473.

HCl·Phe-ΔCys(*S*-triazole)-OMe (10a)

The peptide (0.135 g, 0.30 mmol) was treated with HCl in methanol (1.5 M, 3 ml). The product (0.120 g) was obtained quantitatively.

^{1}H NMR (400 MHz, DMSO) δ 10.50 (s, 1H), 8.70 (s, 1H), 8.41 (d, *J* = 3.9 Hz, 3H), 8.08 (s, 1H), 7.44 – 7.25 (m, 5H), 4.36 – 4.27 (m, 1H), 3.73 (s, 3H), 3.28 (dd, *J* = 14.1, 5.3 Hz, 1H), 3.08 ppm (dd, *J* = 14.1, 8.0 Hz, 1H).
^{13}C NMR (101 MHz, DMSO) δ 167.25, 162.37, 154.90, 145.92, 134.80, 132.67, 129.86, 128.61, 127.30, 122.83, 53.48, 52.57, 37.03.
IR ν (KBr disk, cm^{-1}): 3427, 2953, 2894, 1721, 1695, 1614, 1520, 1498, 1456, 1437, 1329, 1254, 974, 837, 752, 702
HRMS (ESI): *m/z* calcd for $C_{15}H_{17}N_5O_3S+H^+$: 348.1125 [*M*+H]$^+$; found: 348.1128.

HCl·Phe-ΔCys(*S*-benzothiazole)-OMe (10d)

The peptide (0.120 g, 0.23 mmol) was treated with HCl in methanol (1.5 M, 3 ml). The product (0.100 g) was obtained quantitatively. The signals derived from two conformers are visible in NMR spectra (please see attached spectra).

^{1}H NMR (400 MHz, DMSO) δ 10.70 (s, 1H), 8.43 (s, 3H), 8.14 (s, 1H), 8.12 (d, *J* = 7.5 Hz, 1H), 8.01 (d, *J* = 8.0 Hz, 1H), 7.58 – 7.52 (m, 1H), 7.46 (m, 1H), 7.42 – 7.10 (m, 5H), 4.36 (s, 1H), 3.79 (s, 3H), 3.26 (dd, *J* = 14.1, 5.5 Hz, 1H), 3.10 (dd, *J* = 14.1, 8.0 Hz, 1H).
^{13}C NMR (101 MHz, DMSO) δ 167.38, 162.23, 161.83, 152.30, 135.01, 134.67, 129.83, 128.61, 128.07, 127.32, 126.95, 125.44, 124.96, 122.37, 121.97, 53.46, 52.89, 37.02.
IR ν (KBr disk, cm^{-1}): 3430, 3063, 2951, 1720, 1701, 1605, 1498, 1456, 1427, 1327, 1239, 995, 755, 727, 701
HRMS (ESI): *m/z* calcd for $C_{20}H_{19}N_3O_3S_2+H^+$: 414.0940 [*M*+H]$^+$; found: 414.0932.

4. ^{1}H NMR, ^{13}C NMR and IR spectra

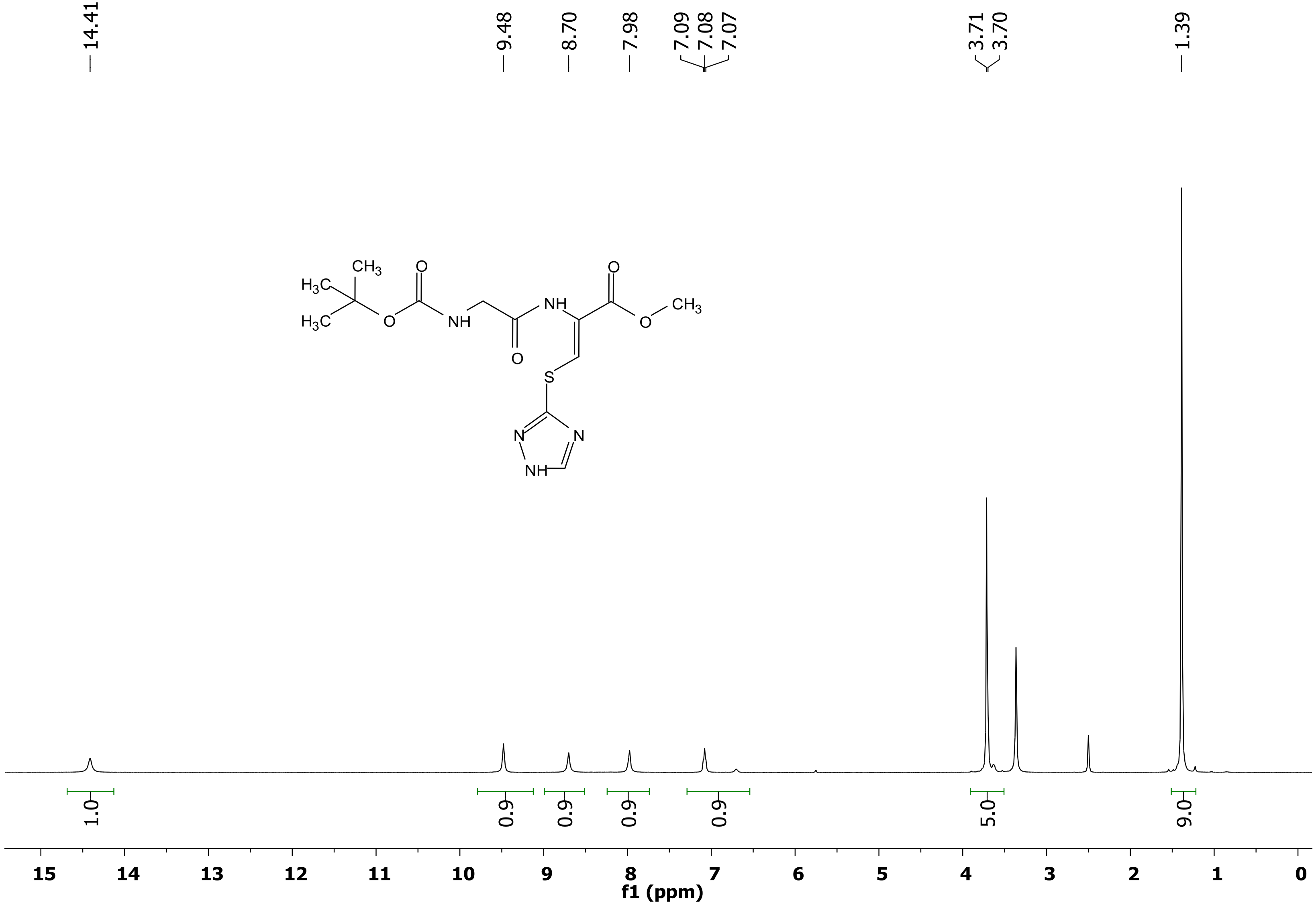

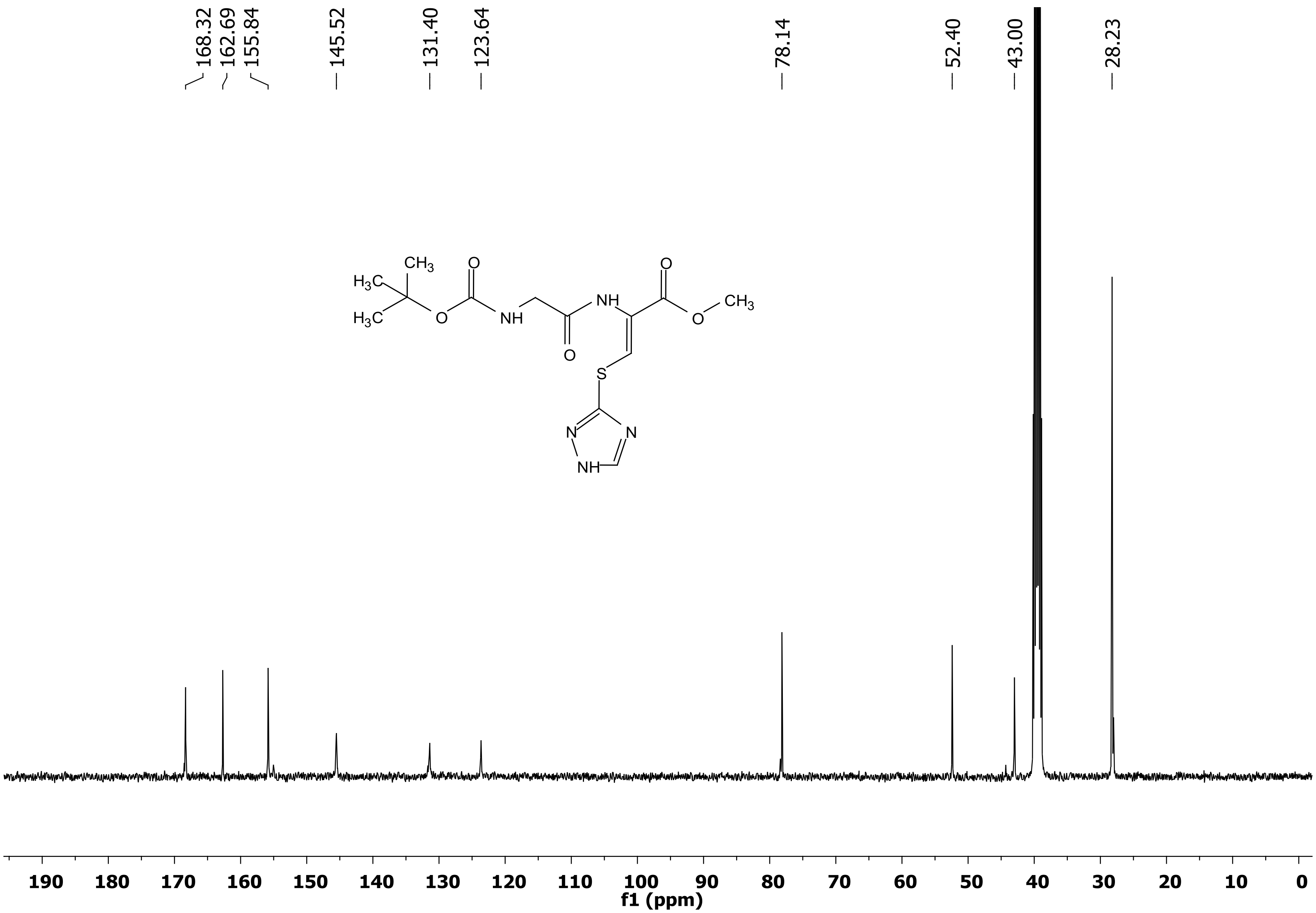
168.32
162.69
155.84
145.52
131.40
123.64
78.14
52.40
43.00
28.23
190 180 170 160 150 140 130 120 110 100 90 80 70 60 50 40 30 20 10 0
f1 (ppm)

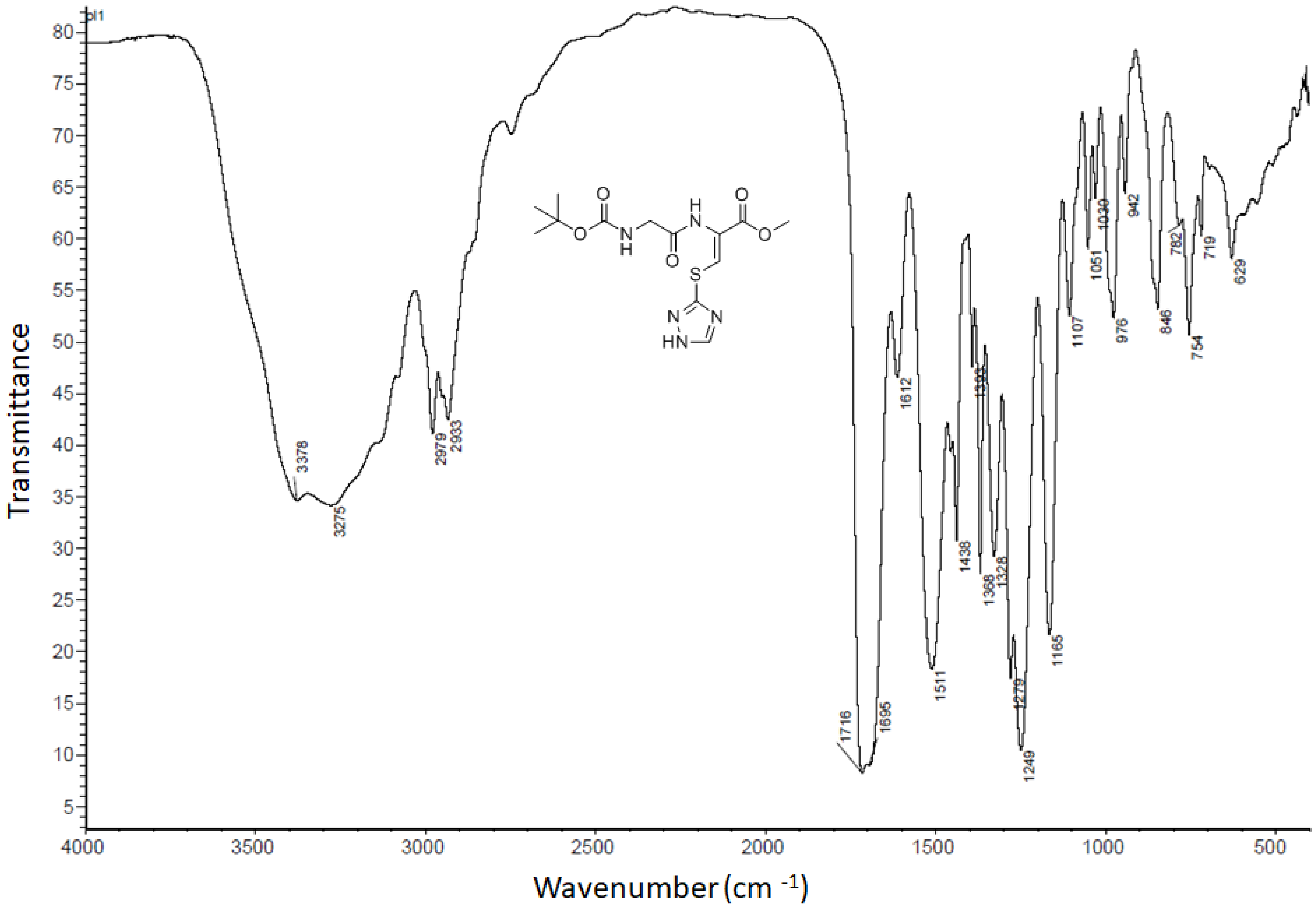
Transmittance
Wavenumber (cm -1)
3378
3275
2979
2933
1716
1695
1612
1511
1438
1368
1328
1279
1249
1165
1107
1051
1030
976
942
846
782
754
719
629

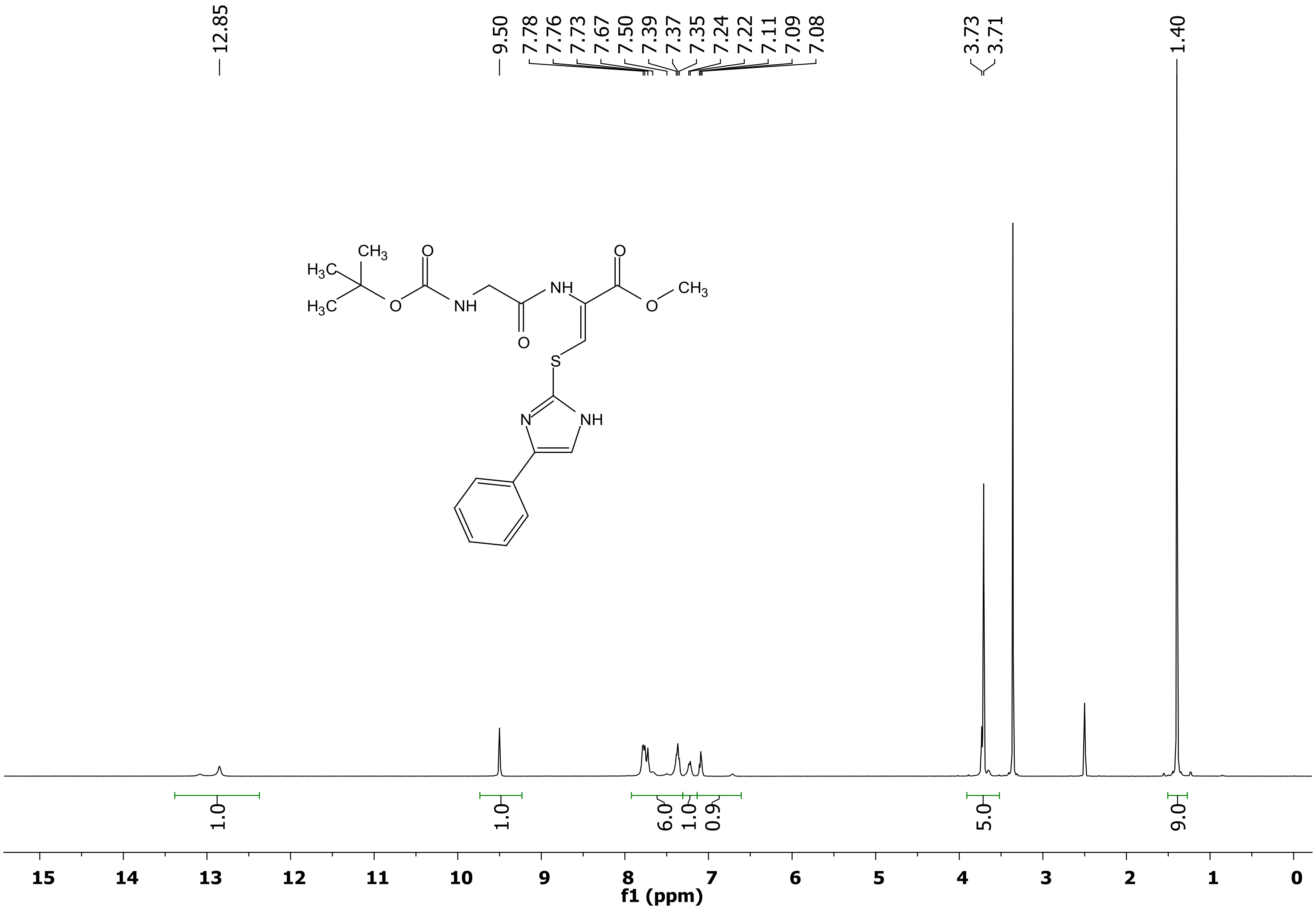
12.85
9.50
7.78
7.76
7.73
7.67
7.50
7.39
7.37
7.35
7.24
7.22
7.11
7.09
7.08
3.73
3.71
1.40
1.0
1.0
6.0
1.0
0.9
5.0
9.0
15
14
13
12
11
10
9
8
7
6
5
4
3
2
1
0
f1 (ppm)

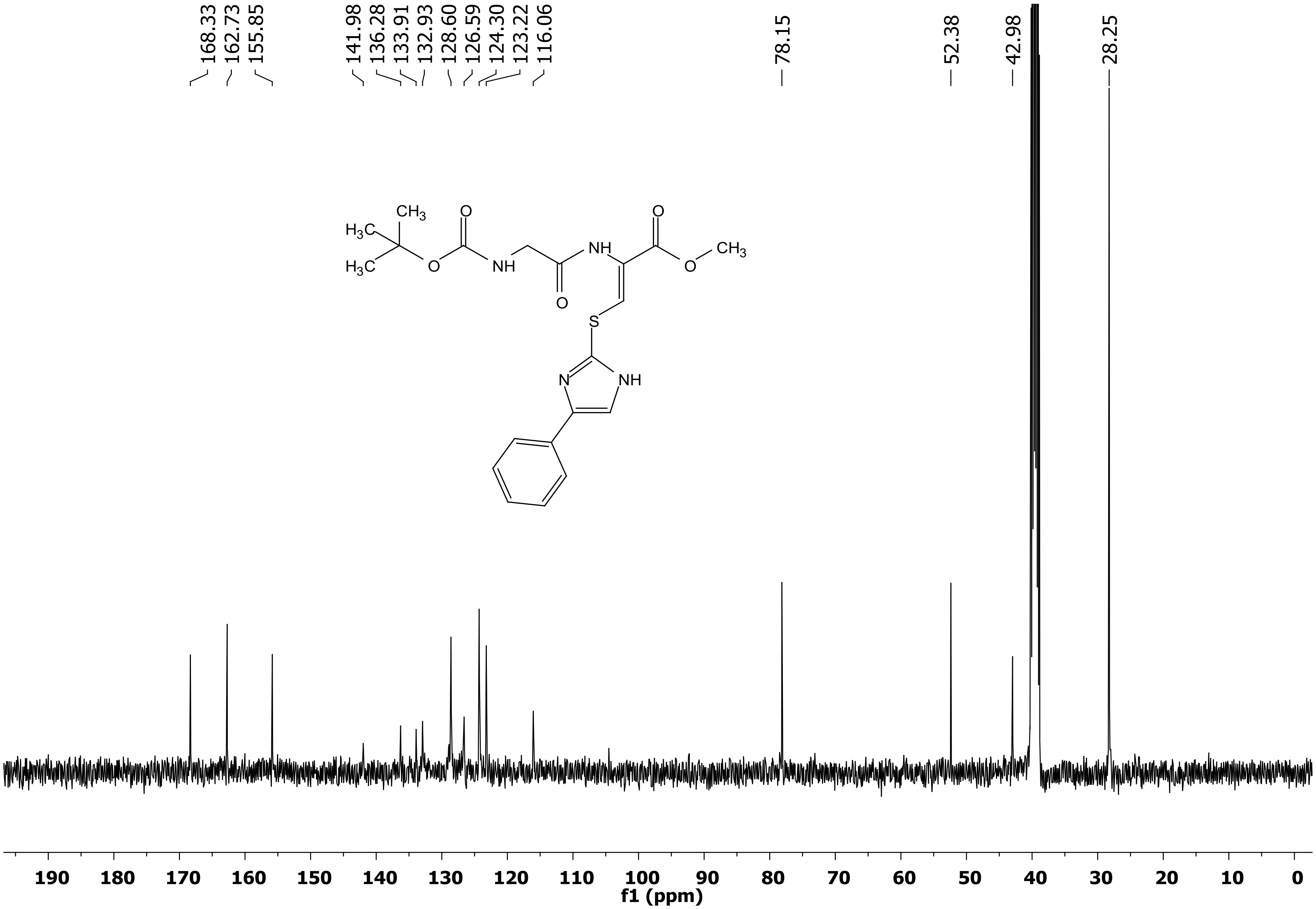
168.33
162.73
155.85
141.98
136.28
133.91
132.93
128.60
126.59
124.30
123.22
116.06
78.15
52.38
42.98
28.25
CH3
H3C
H3C
O
O
NH
NH
O
O
O
CH3
S
N
NH
190
180
170
160
150
140
130
120
110
100
90
80
70
60
50
40
30
20
10
0
f1 (ppm)

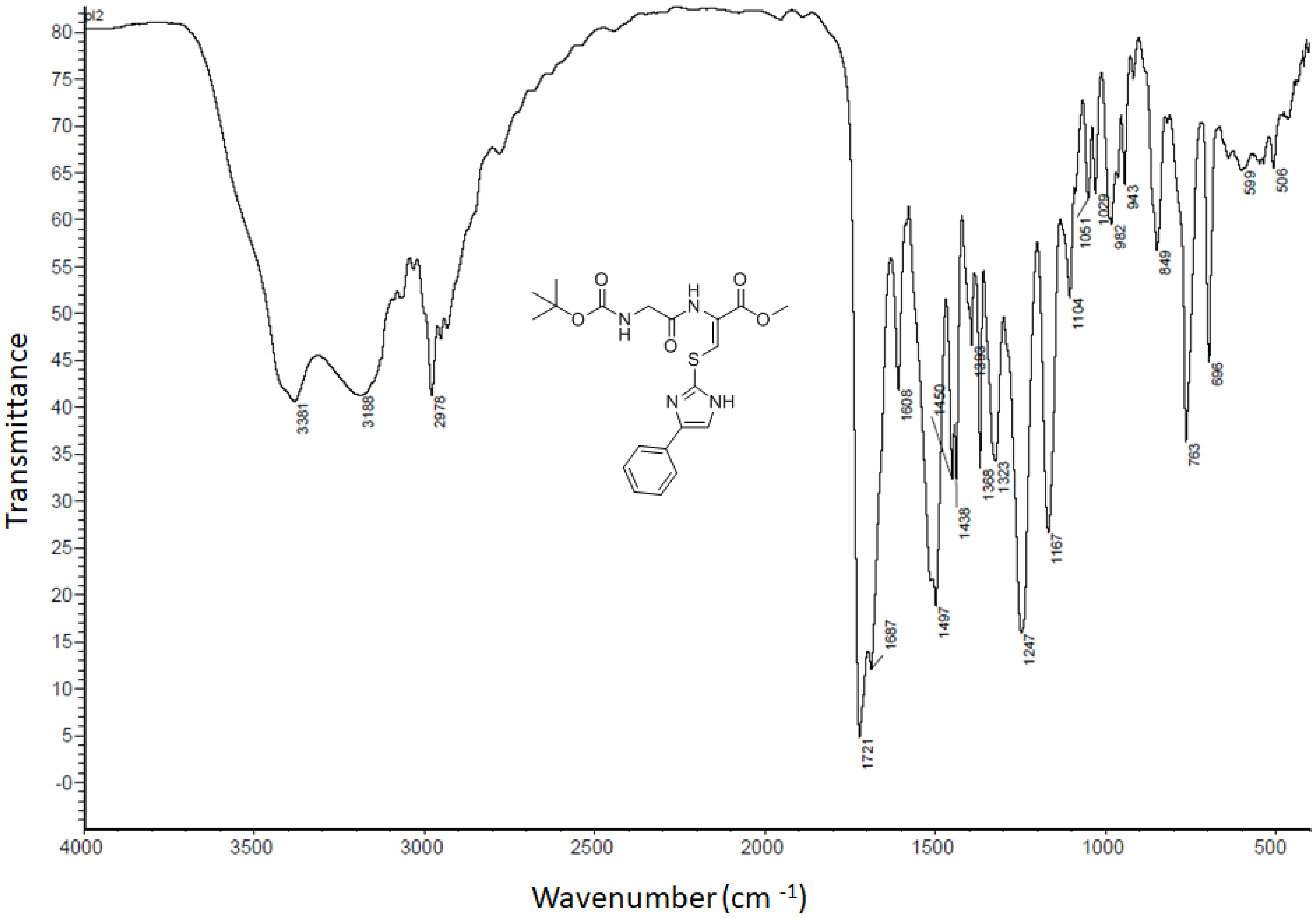
bl2
Transmittance
Wavenumber (cm -1)
80
75
70
65
60
55
50
45
40
35
30
25
20
15
10
5
-0
4000
3500
3000
2500
2000
1500
1000
500
3381
3188
2978
1721
1687
1608
1497
1450
1438
1393
1368
1323
1247
1167
1104
1051
1029
982
943
849
763
696
599
506
O
O
N
H
O
H
N
O
O
S
N
NH

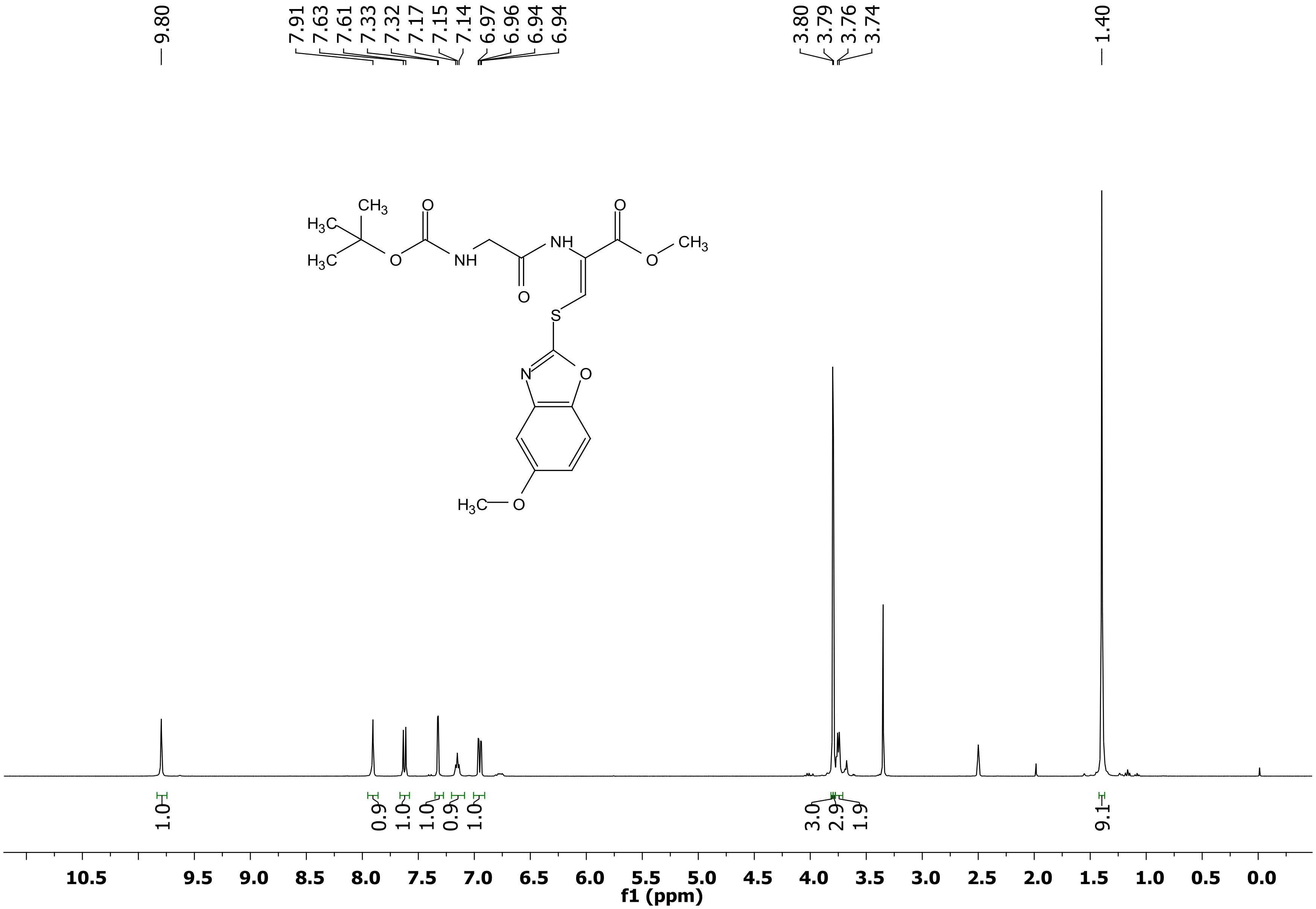

9.80
7.91
7.63
7.61
7.33
7.32
7.17
7.15
7.14
6.97
6.96
6.94
6.94
3.80
3.79
3.76
3.74
1.40
CH3
O
H3C
H3C
O
NH
O
NH
O
O
CH3
S
N
O
H3C
O
1.0
0.9
1.0
1.0
0.9
1.0
3.0
2.9
1.9
9.1
10.5
9.5
9.0
8.5
8.0
7.5
7.0
6.5
6.0
5.5
5.0
4.5
4.0
3.5
3.0
2.5
2.0
1.5
1.0
0.5
0.0
f1 (ppm)

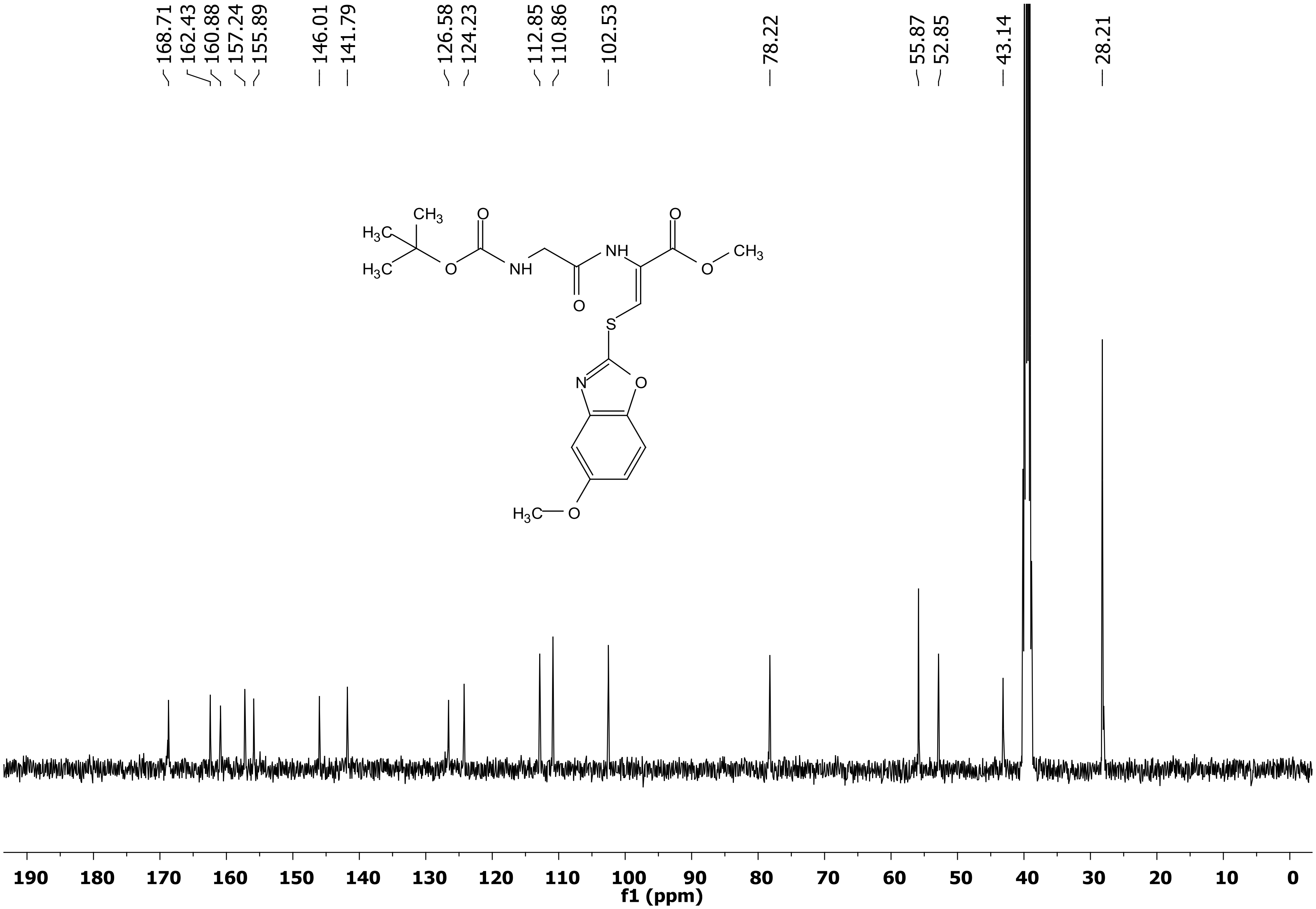

168.71
162.43
160.88
157.24
155.89
146.01
141.79
126.58
124.23
112.85
110.86
102.53
78.22
55.87
52.85
43.14
28.21
190 180 170 160 150 140 130 120 110 100 90 80 70 60 50 40 30 20 10 0
f1 (ppm)

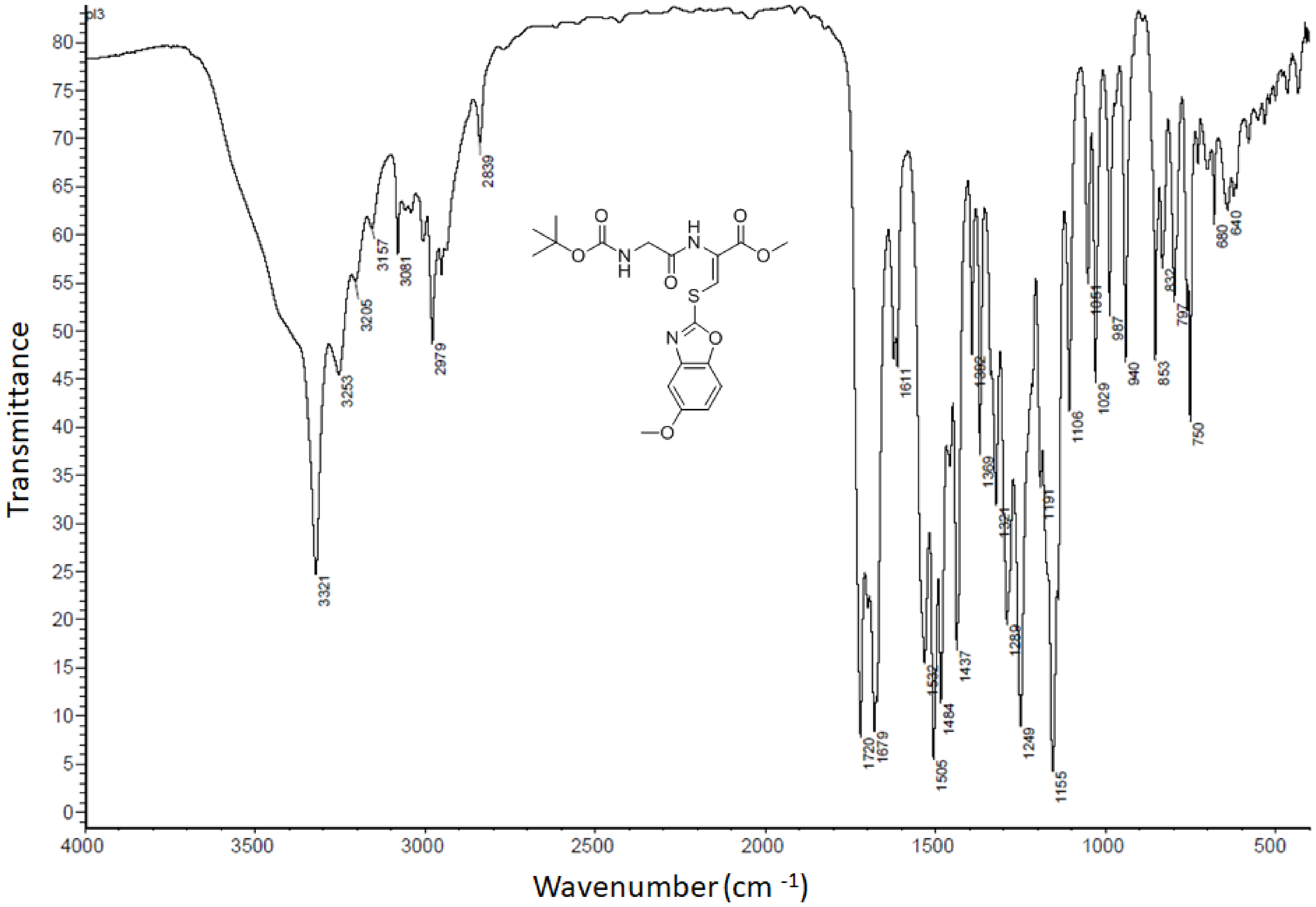
Transmittance
Wavenumber (cm -1)
4000
3500
3000
2500
2000
1500
1000
500
3321
3253
3205
3157
3081
2979
2839
1720
1679
1611
1532
1505
1484
1437
1382
1369
1321
1289
1249
1191
1155
1106
1051
1029
987
940
853
832
797
750
680
640

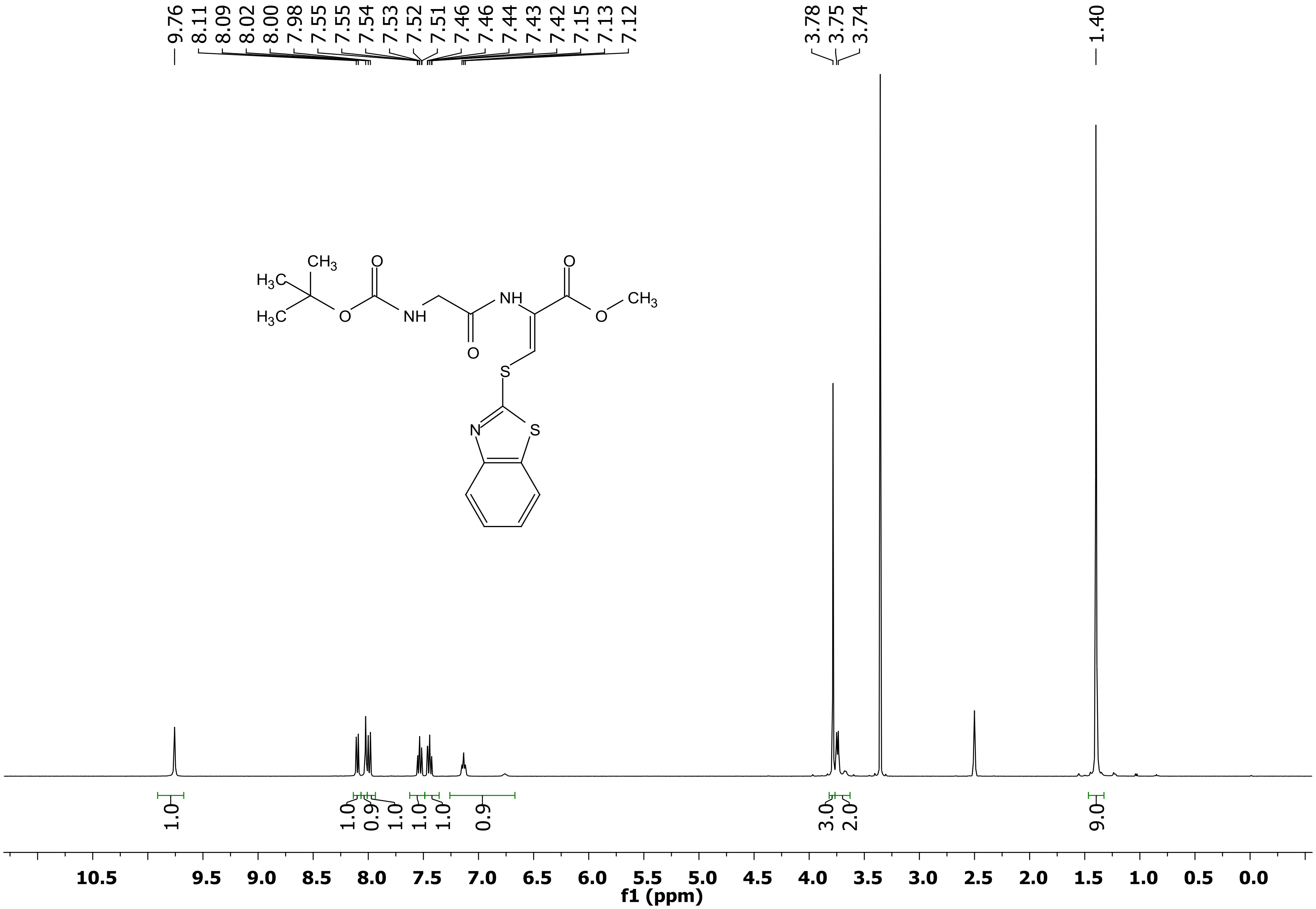
9.76
8.11
8.09
8.02
8.00
7.98
7.55
7.55
7.54
7.53
7.52
7.51
7.46
7.46
7.44
7.43
7.42
7.15
7.13
7.12
3.78
3.75
3.74
1.40
CH3
H3C
H3C
O
O
NH
NH
O
O
O
CH3
S
N
S
1.0
1.0
0.9
1.0
1.0
1.0
0.9
3.0
2.0
9.0
10.5
9.5
9.0
8.5
8.0
7.5
7.0
6.5
6.0
5.5
5.0
4.5
4.0
3.5
3.0
2.5
2.0
1.5
1.0
0.5
0.0
f1 (ppm)

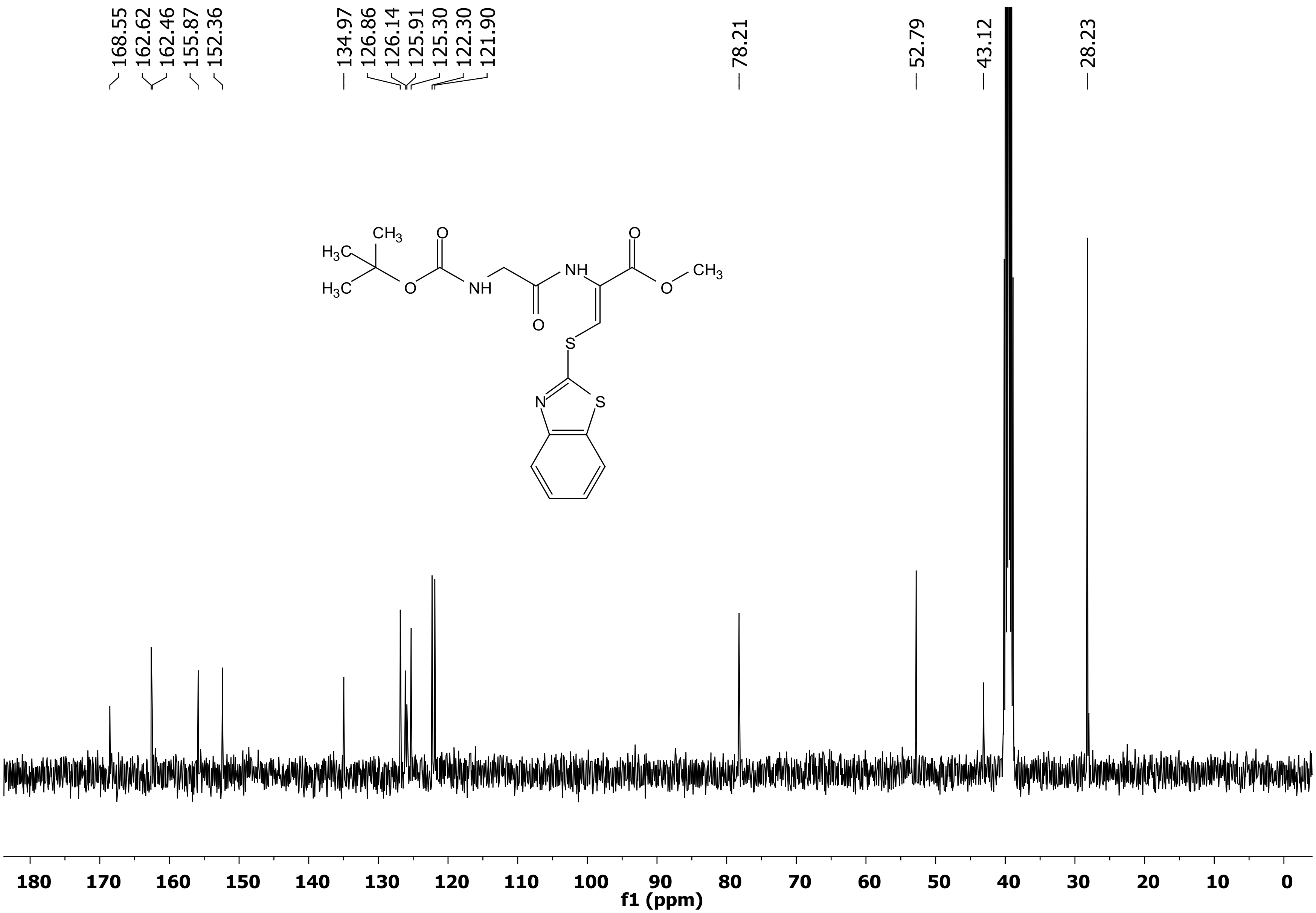
168.55
162.62
162.46
155.87
152.36
134.97
126.86
126.14
125.91
125.30
122.30
121.90
78.21
52.79
43.12
28.23
CH3
H3C
H3C
O
NH
NH
O
O
O
CH3
S
N
S
180
170
160
150
140
130
120
110
100
90
80
70
60
50
40
30
20
10
0
f1 (ppm)

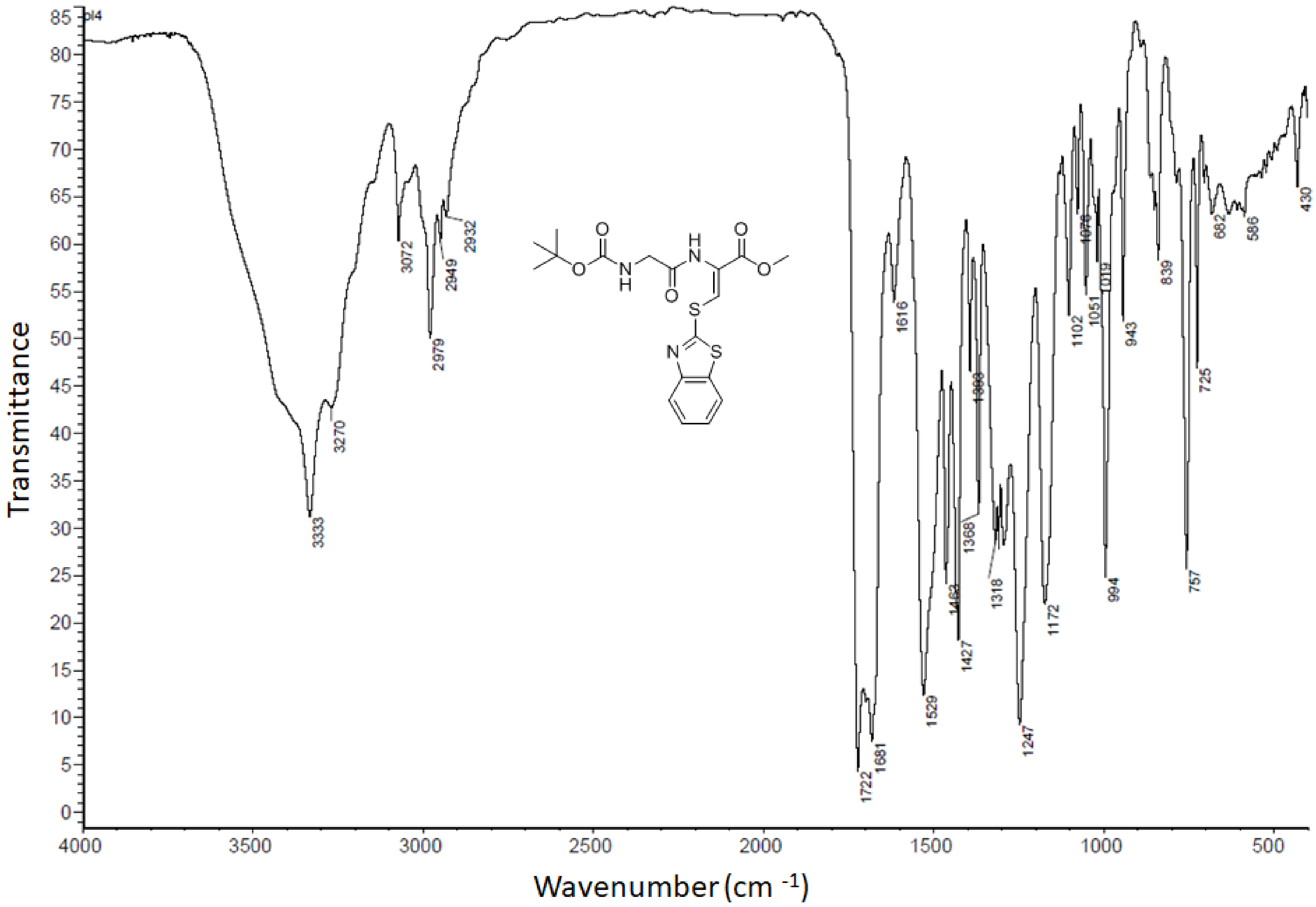
pl4
Transmittance
Wavenumber (cm -1)
3333
3270
3072
2979
2949
2932
1722
1681
1616
1529
1463
1427
1368
1360
1318
1247
1172
1102
1076
1051
1019
994
943
839
757
725
682
586
430
N
H
O
S

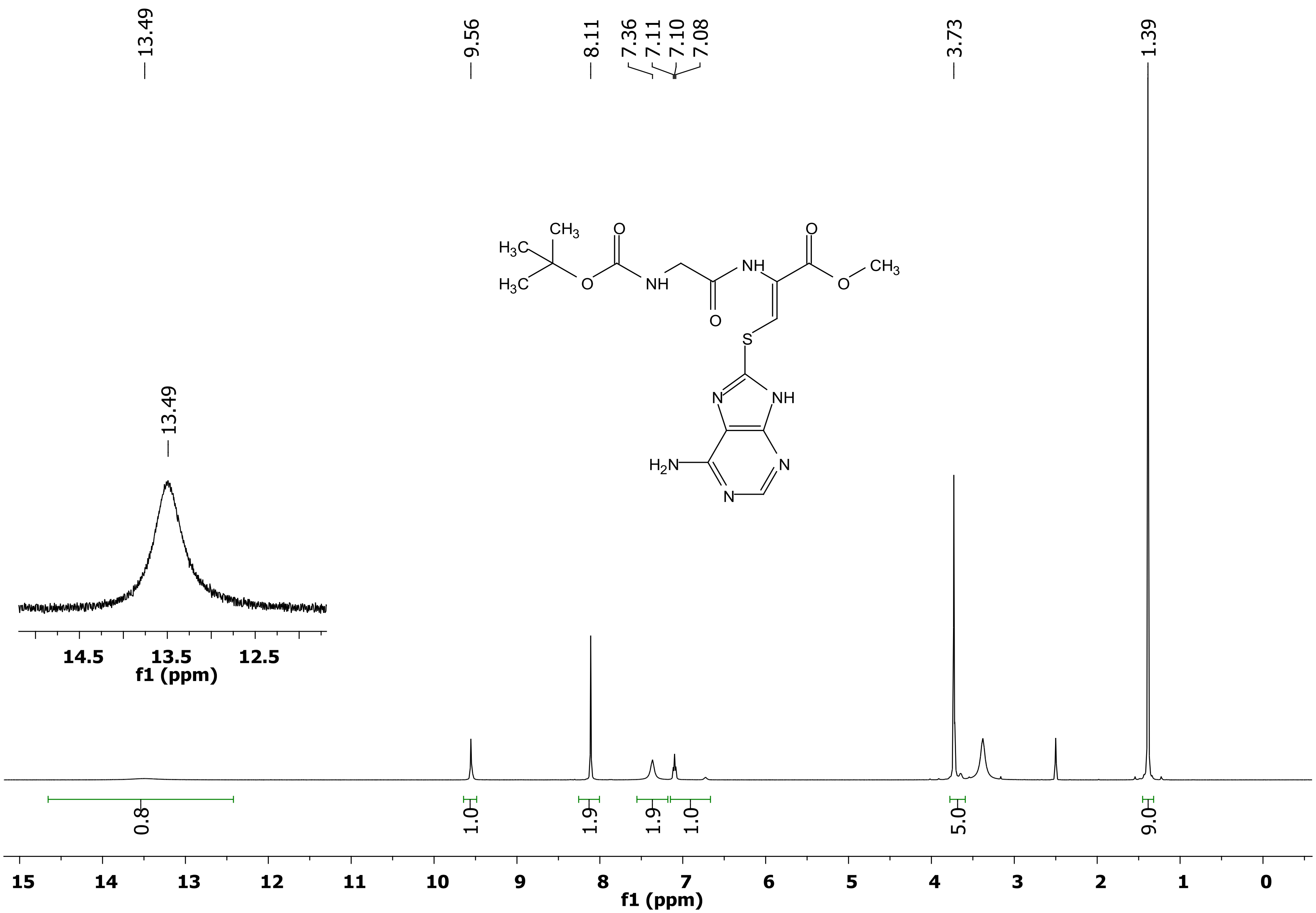

13.49
9.56
8.11
7.36
7.11
7.10
7.08
3.73
1.39
0.8
1.0
1.9
1.9
1.0
5.0
9.0
14.5
13.5
12.5
f1 (ppm)
15
14
13
12
11
10
9
8
7
6
5
4
3
2
1
0
f1 (ppm)

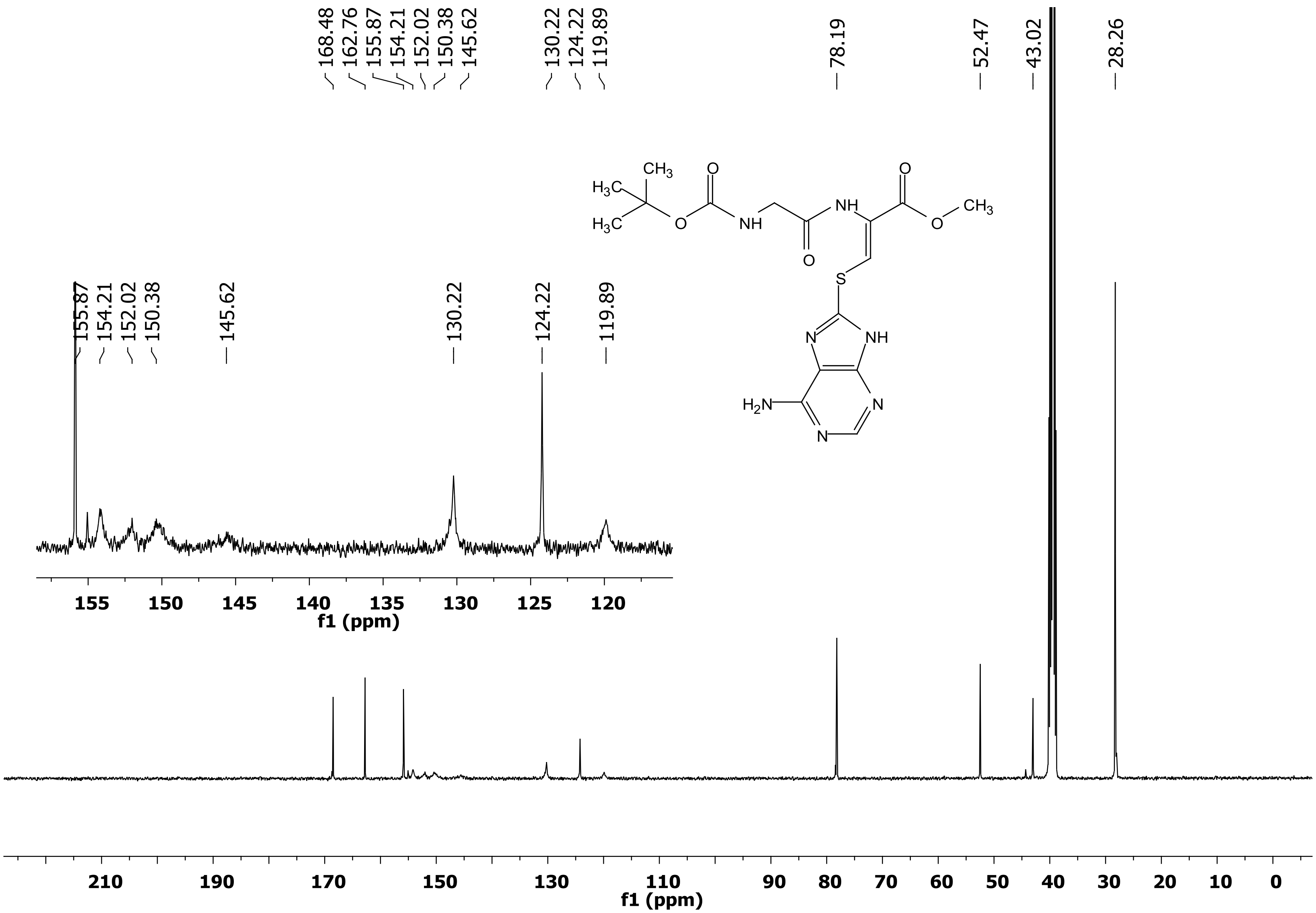
168.48
162.76
155.87
154.21
152.02
150.38
145.62
130.22
124.22
119.89
78.19
52.47
43.02
28.26
H_3C
CH_3
H_3C
O
NH
NH
O
O
CH_3
S
N
NH
H_2N
N
N
155
150
145
140
135
130
125
120
f1 (ppm)
210
190
170
150
130
110
90
80
70
60
50
40
30
20
10
0
f1 (ppm)

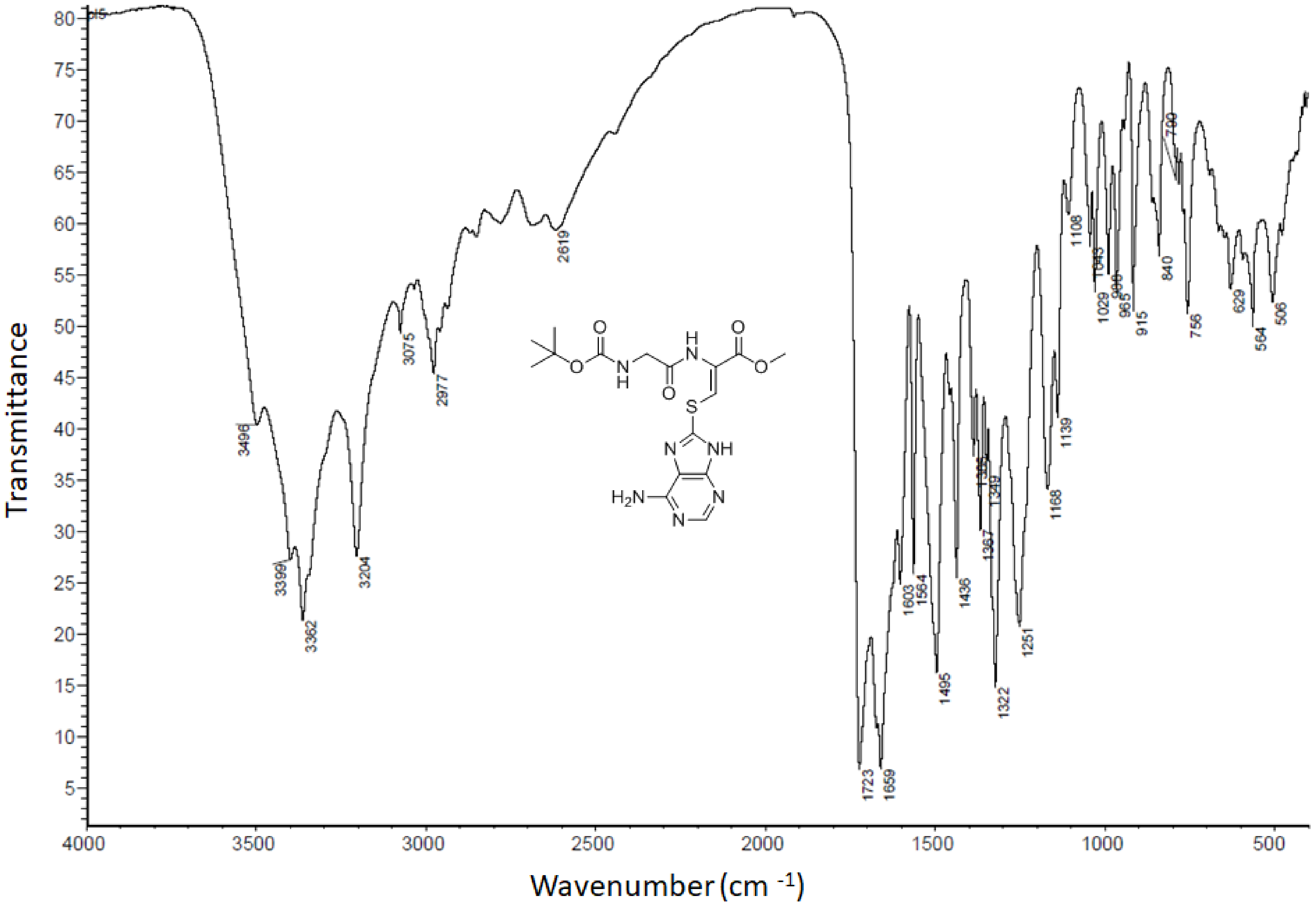

Transmittance
Wavenumber (cm -1)
4000
3500
3000
2500
2000
1500
1000
500
80
75
70
65
60
55
50
45
40
35
30
25
20
15
10
5
3496
3399
3362
3204
3075
2977
2619
1723
1659
1603
1564
1495
1436
1385
1367
1349
1322
1251
1168
1139
1108
1043
1029
990
965
915
840
790
756
629
564
506
O
H
N
S
NH
H2N

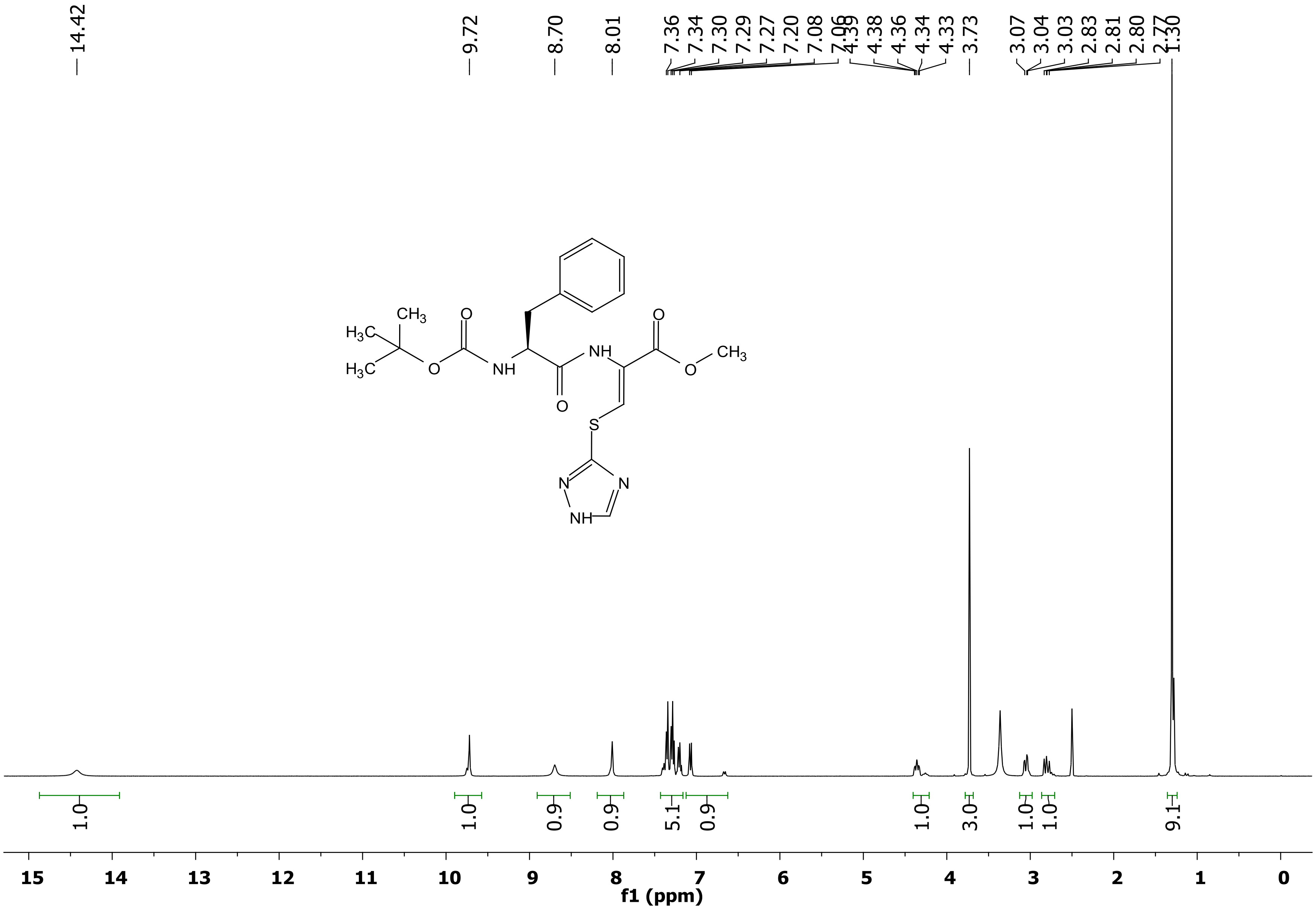

14.42
9.72
8.70
8.01
7.36
7.34
7.30
7.29
7.27
7.20
7.08
7.06
4.39
4.38
4.36
4.34
4.33
3.73
3.07
3.04
3.03
2.83
2.81
2.80
2.77
1.30
1.0
1.0
0.9
0.9
5.1
0.9
1.0
3.0
1.0
1.0
9.1
f1 (ppm)

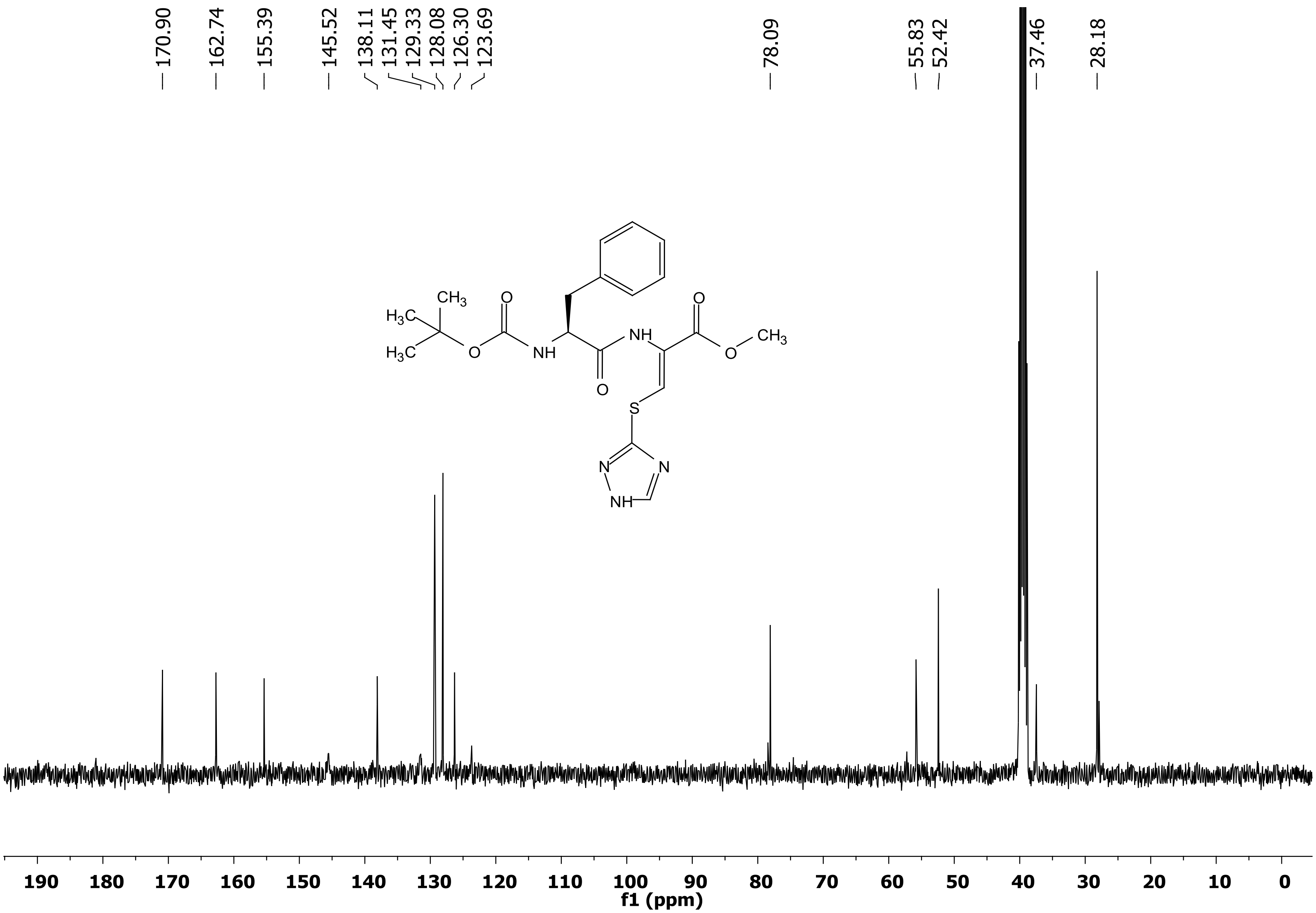
170.90
162.74
155.39
145.52
138.11
131.45
129.33
128.08
126.30
123.69
78.09
55.83
52.42
37.46
28.18
CH3
H3C
H3C
O
O
NH
O
NH
O
O
CH3
S
N
N
NH
190 180 170 160 150 140 130 120 110 100 90 80 70 60 50 40 30 20 10 0
f1 (ppm)

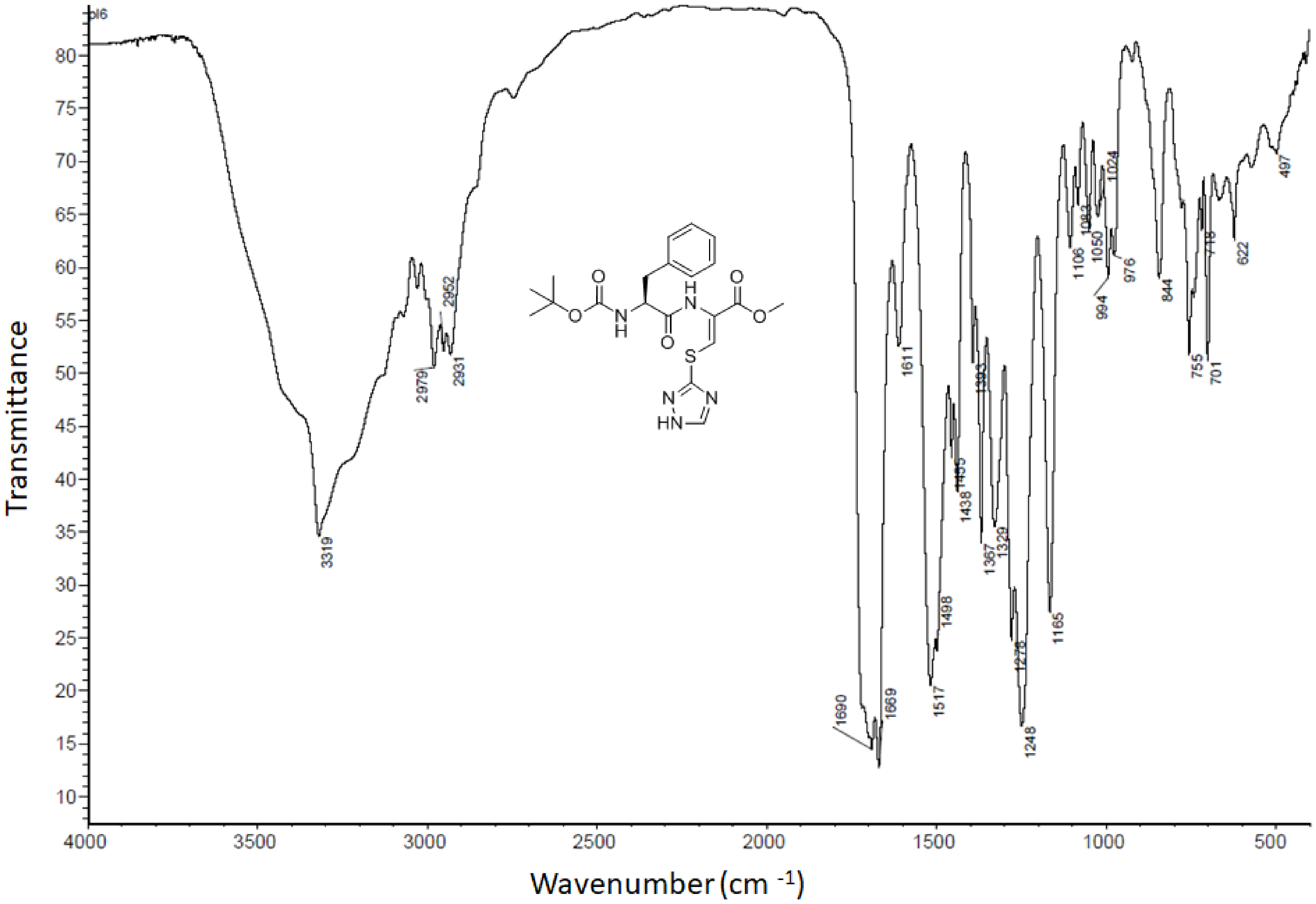

Transmittance
Wavenumber (cm -1)
4000
3500
3000
2500
2000
1500
1000
500
80
75
70
65
60
55
50
45
40
35
30
25
20
15
10
3319
2979
2952
2931
1690
1669
1611
1517
1498
1438
1367
1329
1248
1165
1106
1050
1024
994
976
844
755
701
622
497

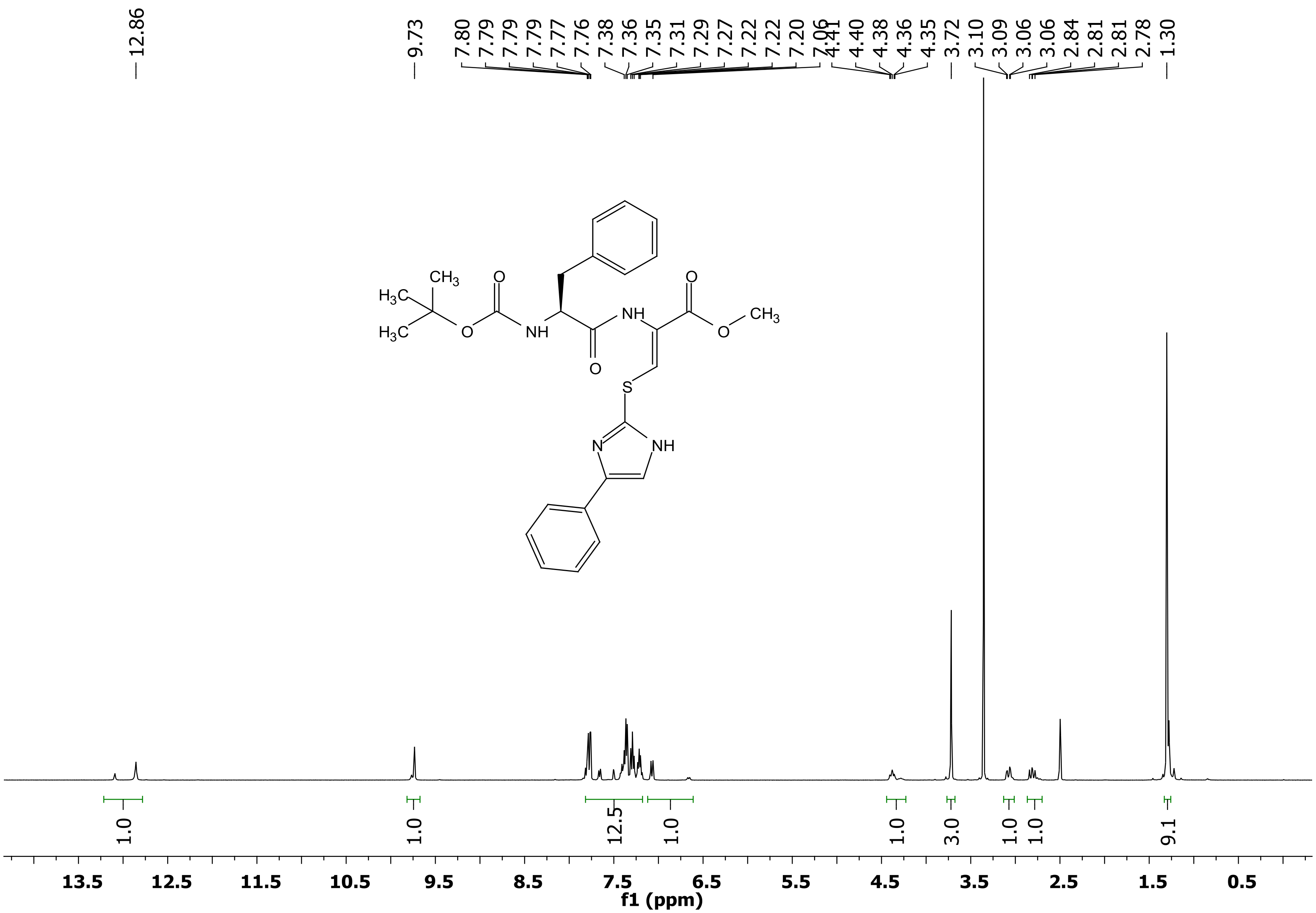
12.86
9.73
7.80
7.79
7.79
7.79
7.77
7.76
7.38
7.36
7.35
7.31
7.29
7.27
7.22
7.22
7.20
7.06
4.41
4.40
4.38
4.36
4.35
3.72
3.10
3.09
3.06
3.06
2.84
2.81
2.81
2.78
1.30
CH_3
O
H_3C
H_3C
O
NH
O
NH
O
CH_3
O
S
N
NH
1.0
1.0
12.5
1.0
1.0
3.0
1.0
1.0
9.1
13.5
12.5
11.5
10.5
9.5
8.5
7.5
6.5
5.5
4.5
3.5
2.5
1.5
0.5
f1 (ppm)

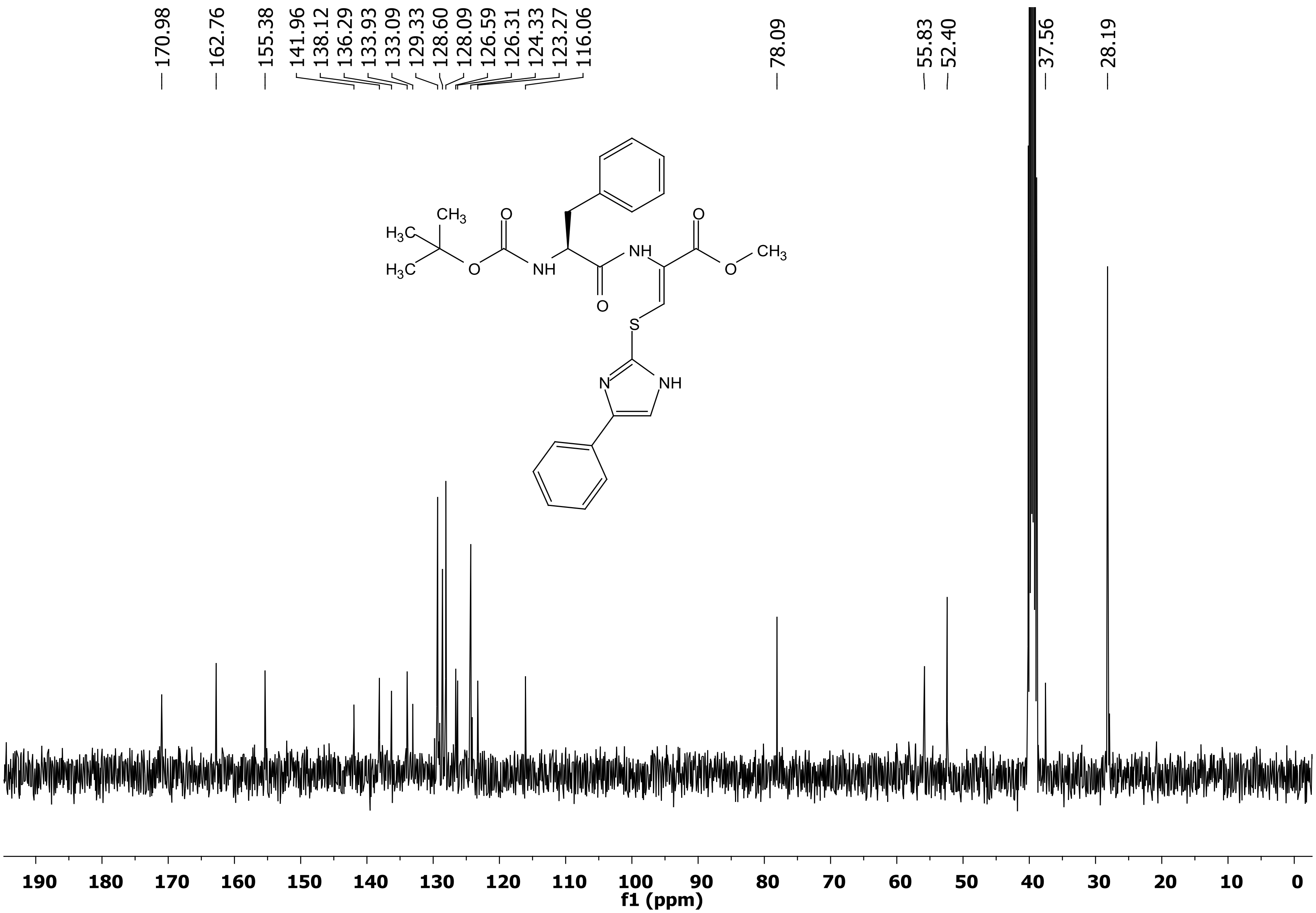
170.98
162.76
155.38
141.96
138.12
136.29
133.93
133.09
129.33
128.60
128.09
126.59
126.31
124.33
123.27
116.06
78.09
55.83
52.40
37.56
28.19
CH3
H3C
H3C
O
O
NH
NH
O
O
CH3
S
N
NH
190 180 170 160 150 140 130 120 110 100 90 80 70 60 50 40 30 20 10 0
f1 (ppm)

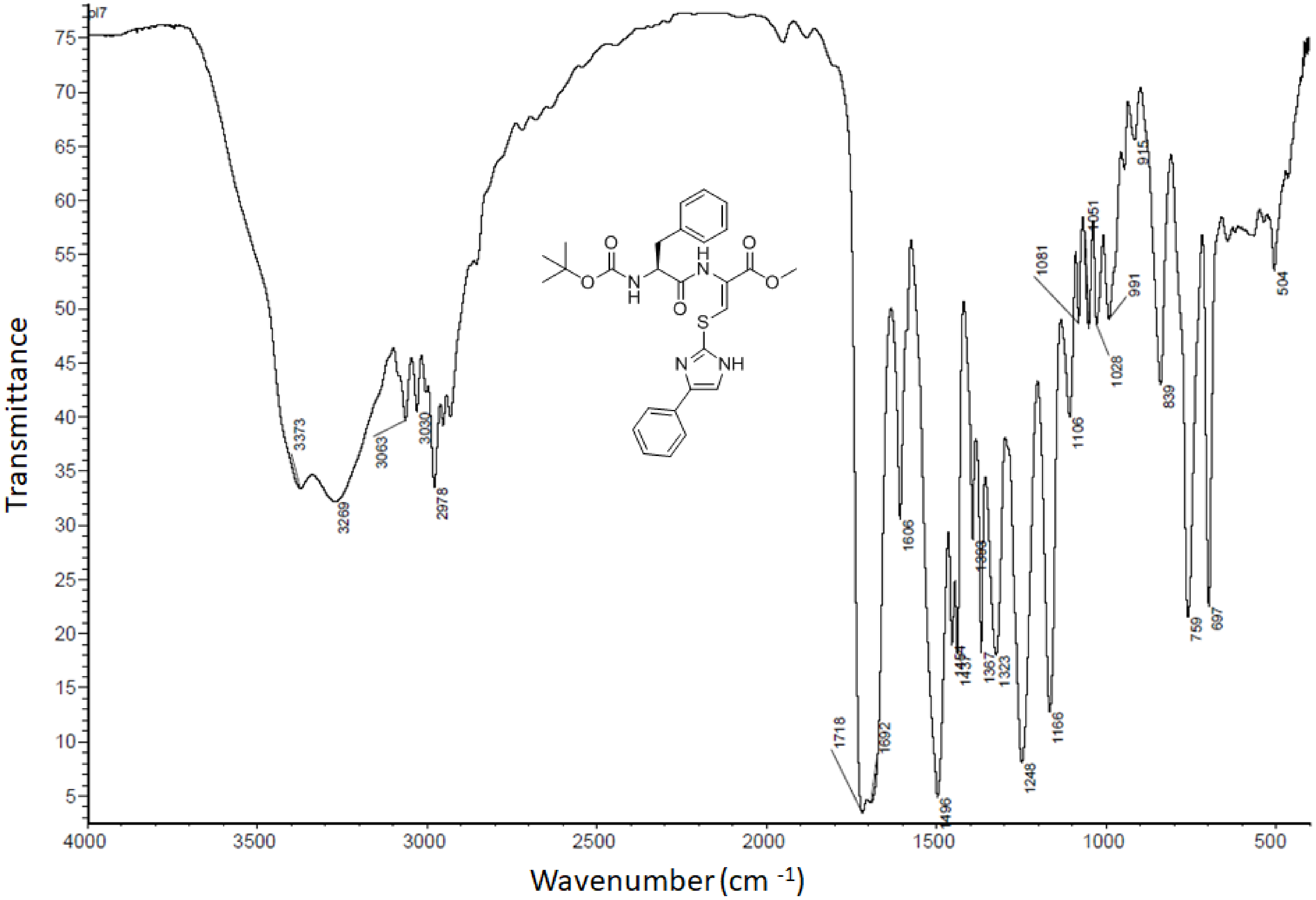

pl7
Transmittance
Wavenumber (cm -1)
75
70
65
60
55
50
45
40
35
30
25
20
15
10
5
4000
3500
3000
2500
2000
1500
1000
500
3373
3269
3063
3030
2978
1718
1692
1606
1437
1367
1323
1248
1166
1106
1081
1051
1028
991
915
839
759
697
504
O
H
N
NH
S

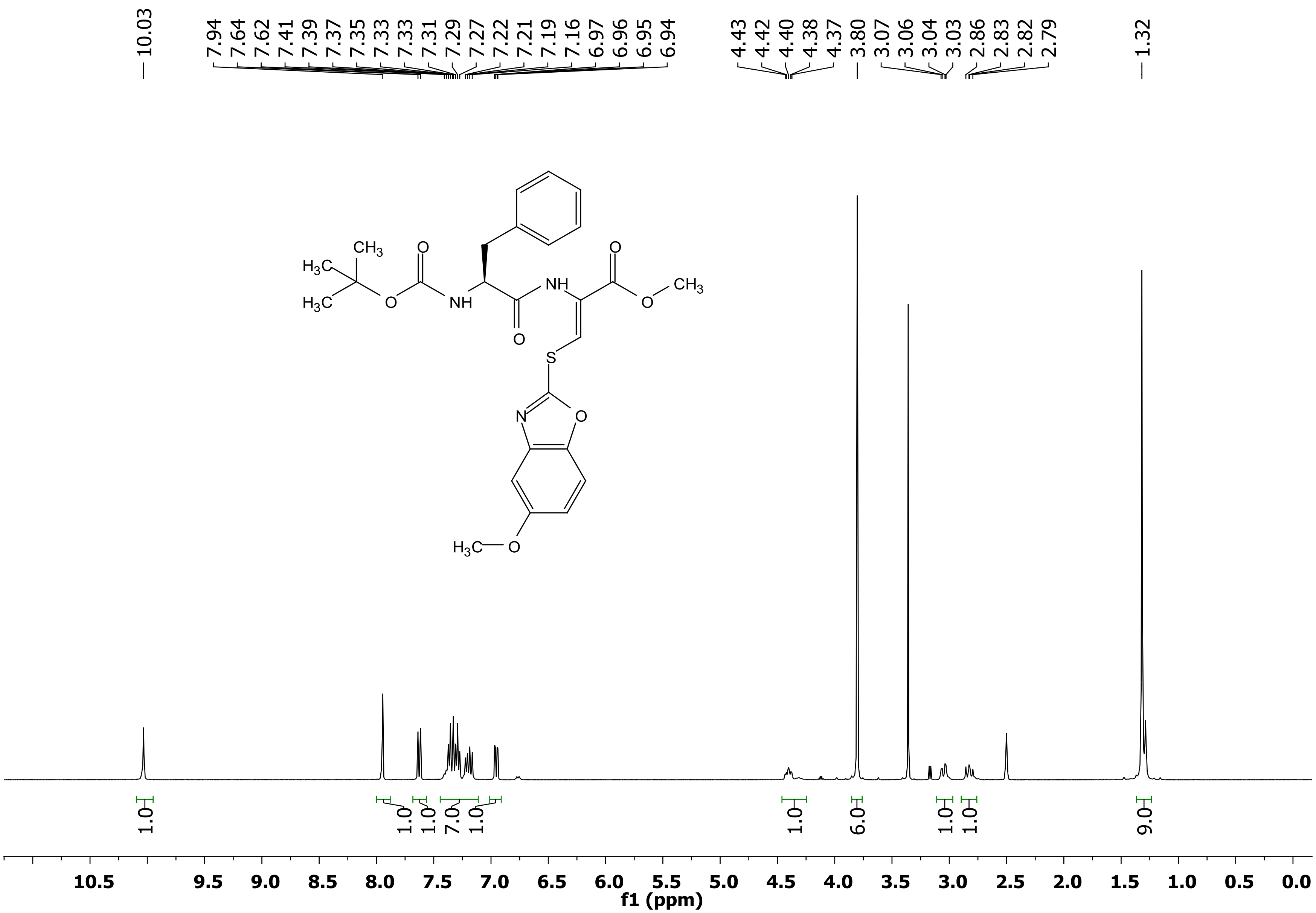
10.03
7.94 7.64 7.62 7.41 7.39 7.37 7.35 7.33 7.33 7.31 7.29 7.27 7.22 7.21 7.19 7.16 6.97 6.96 6.95 6.94
4.43 4.42 4.40 4.38 4.37 3.80 3.07 3.06 3.04 3.03 2.86 2.83 2.82 2.79
1.32
1.0 1.0 1.0 7.0 1.0 1.0 6.0 1.0 1.0 9.0
10.5 9.5 9.0 8.5 8.0 7.5 7.0 6.5 6.0 5.5 5.0 4.5 4.0 3.5 3.0 2.5 2.0 1.5 1.0 0.5 0.0
f1 (ppm)

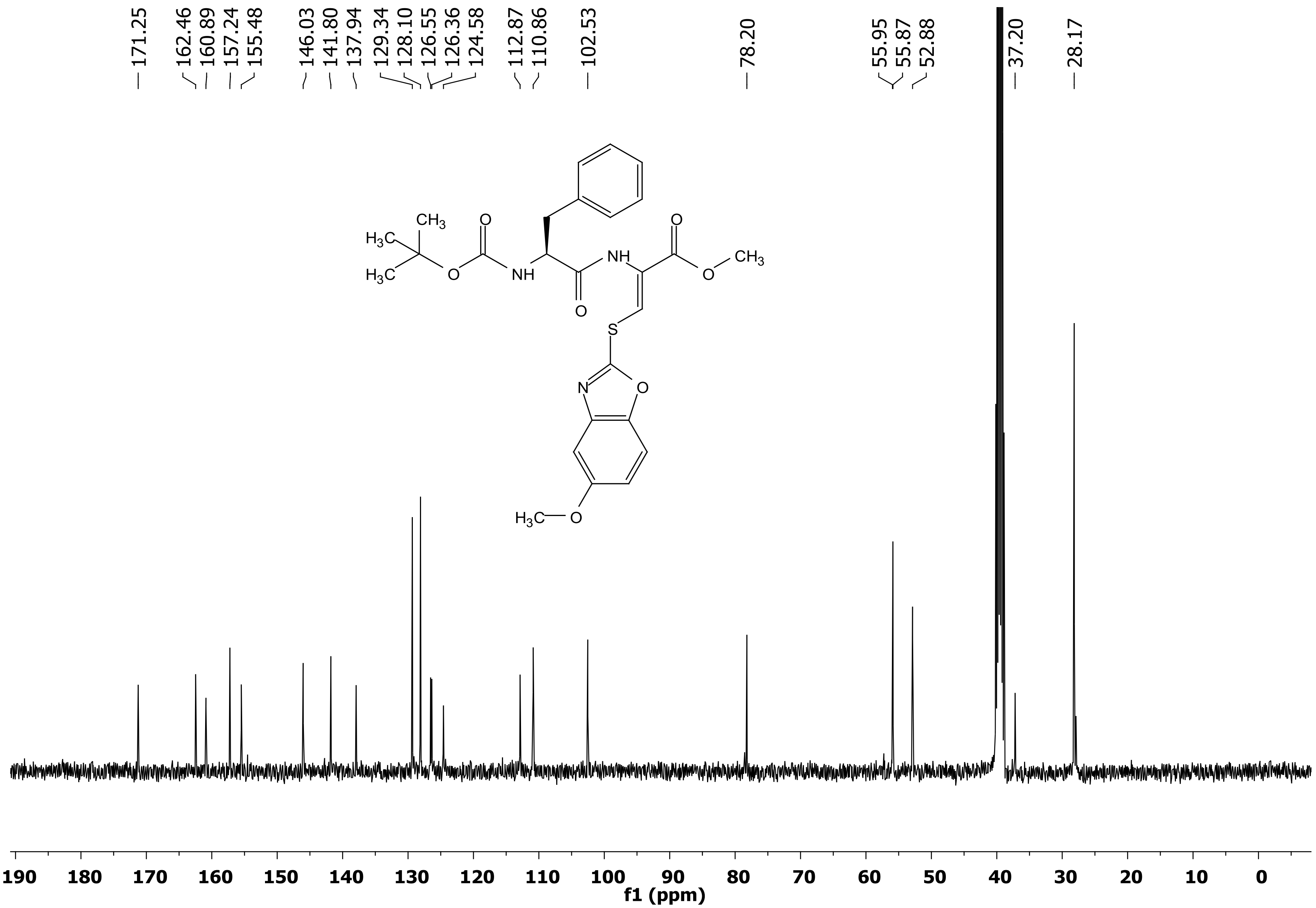
171.25
162.46
160.89
157.24
155.48
146.03
141.80
137.94
129.34
128.10
126.55
126.36
124.58
112.87
110.86
102.53
78.20
55.95
55.87
52.88
37.20
28.17
CH3
H3C
H3C
O
O
NH
O
NH
O
CH3
O
S
N
O
H3C
O
190
180
170
160
150
140
130
120
110
100
90
80
70
60
50
40
30
20
10
0
f1 (ppm)

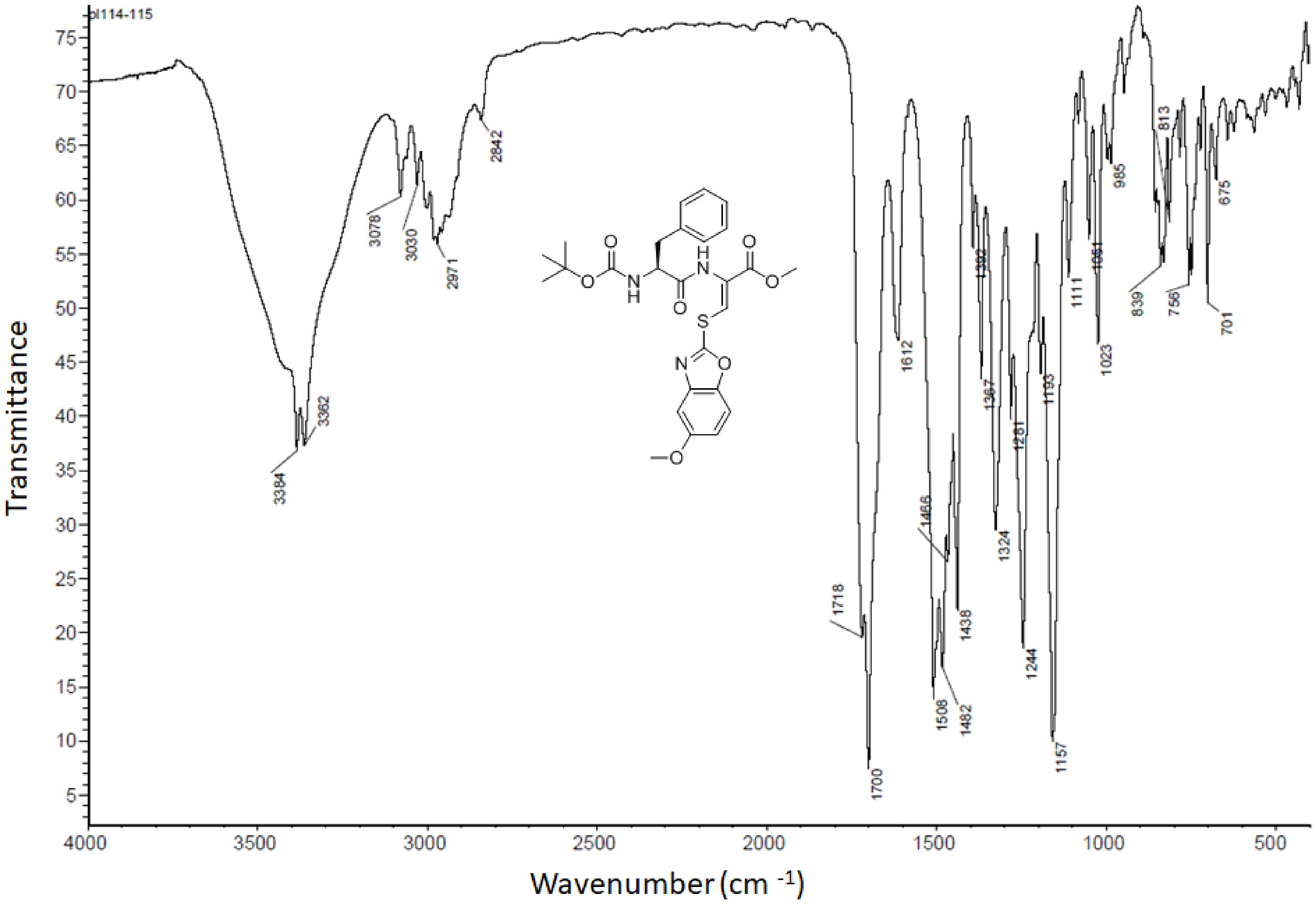

pl114-115
Transmittance
Wavenumber (cm -1)
3384
3362
3078
3030
2971
2842
1718
1700
1612
1508
1482
1466
1438
1392
1367
1324
1281
1244
1193
1157
1111
1061
1023
985
839
813
756
701
675

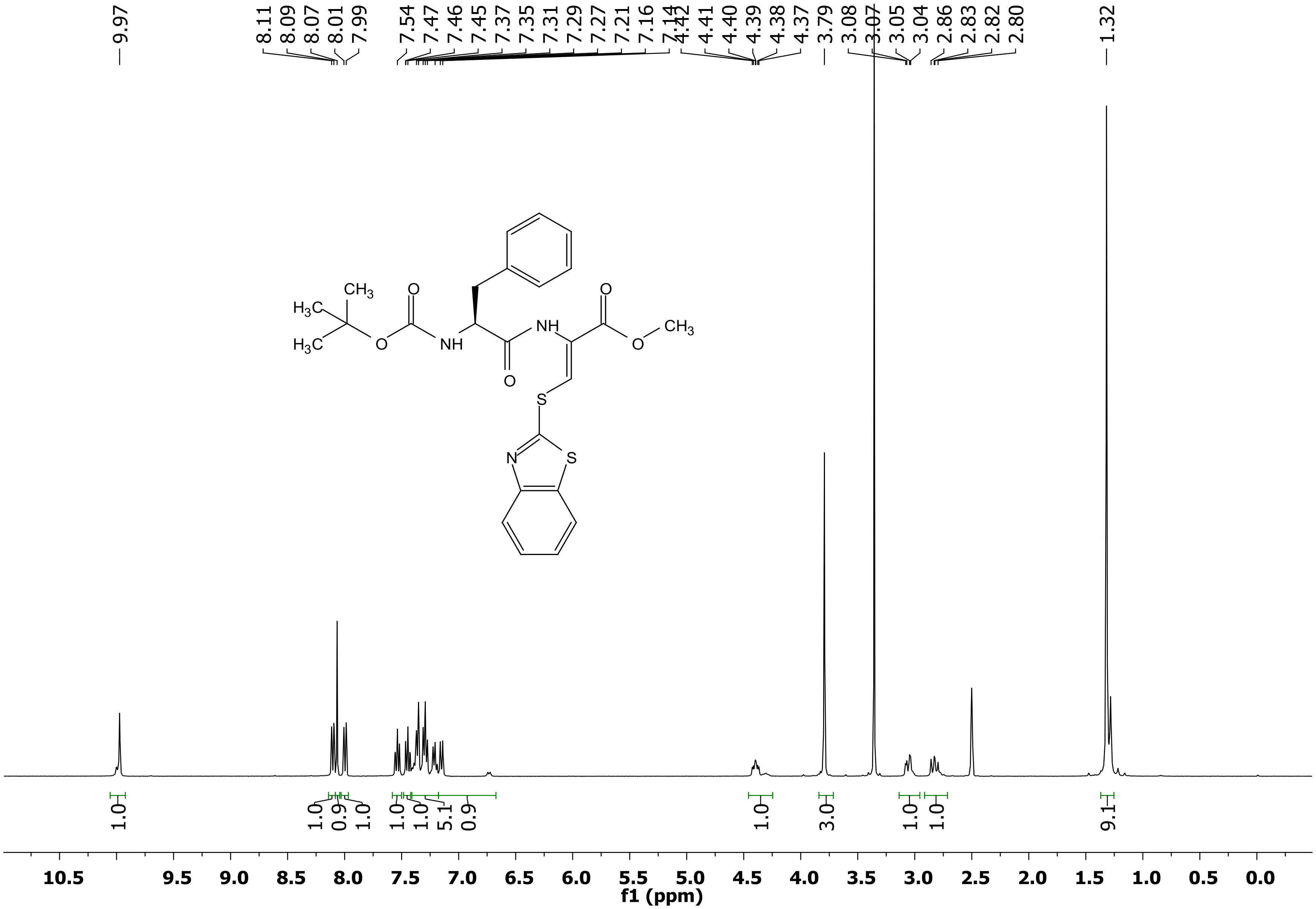
9.97
8.11
8.09
8.07
8.01
7.99
7.54
7.47
7.46
7.45
7.37
7.35
7.31
7.29
7.27
7.21
7.16
7.14
4.42
4.41
4.40
4.39
4.38
4.37
3.79
3.08
3.07
3.05
3.04
2.86
2.83
2.82
2.80
1.32
CH3
H3C
H3C
O
O
NH
O
NH
O
O
CH3
S
N
S
1.0
1.0
0.9
1.0
1.0
1.0
5.1
0.9
1.0
3.0
1.0
1.0
9.1
10.5
9.5
9.0
8.5
8.0
7.5
7.0
6.5
6.0
5.5
5.0
4.5
4.0
3.5
3.0
2.5
2.0
1.5
1.0
0.5
0.0
f1 (ppm)

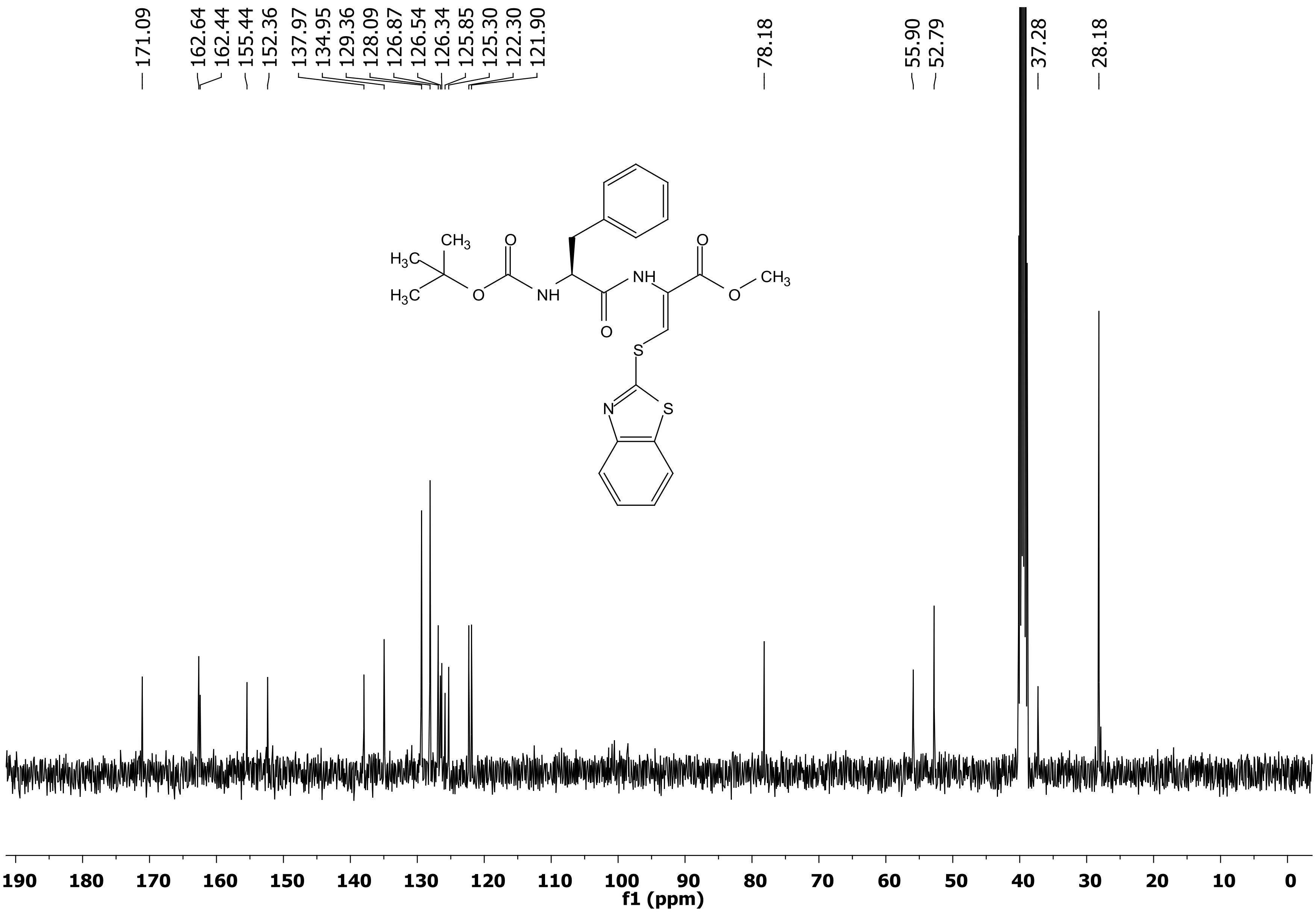
171.09
162.64
162.44
155.44
152.36
137.97
134.95
129.36
128.09
126.87
126.54
126.34
125.85
125.30
122.30
121.90
78.18
55.90
52.79
37.28
28.18
190 180 170 160 150 140 130 120 110 100 90 80 70 60 50 40 30 20 10 0
f1 (ppm)

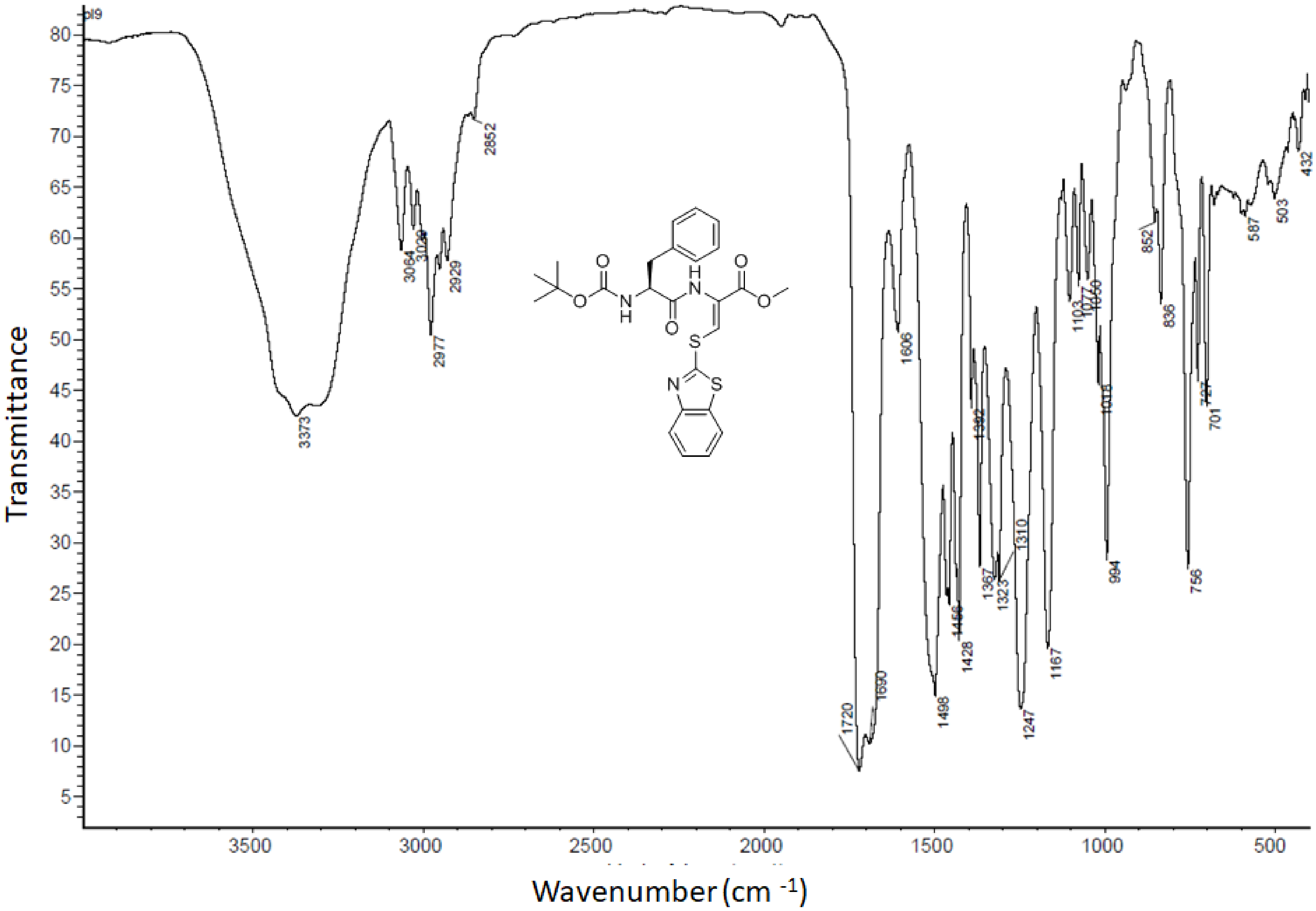

Transmittance
Wavenumber (cm -1)
3373
3064
2977
2929
2852
1720
1690
1606
1498
1428
1382
1367
1323
1310
1247
1167
1103
1015
994
852
836
756
701
587
503
432
N
S
S
H
N
O
O

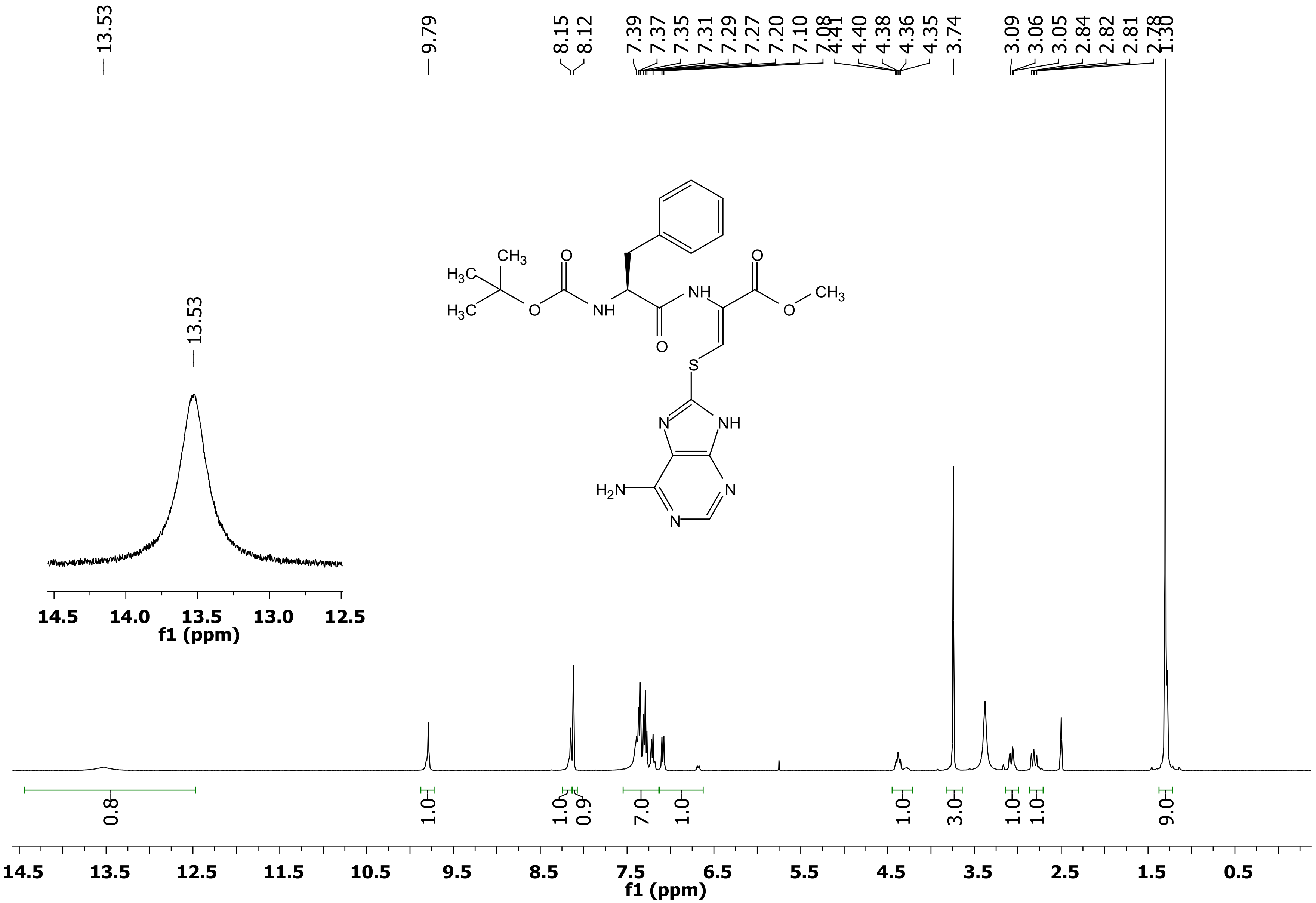
13.53
9.79
8.15
8.12
7.39
7.37
7.35
7.31
7.29
7.27
7.20
7.10
7.08
4.41
4.40
4.38
4.36
4.35
3.74
3.09
3.06
3.05
2.84
2.82
2.81
2.78
1.30
0.8
1.0
1.0
0.9
7.0
1.0
1.0
3.0
1.0
1.0
9.0
f1 (ppm)

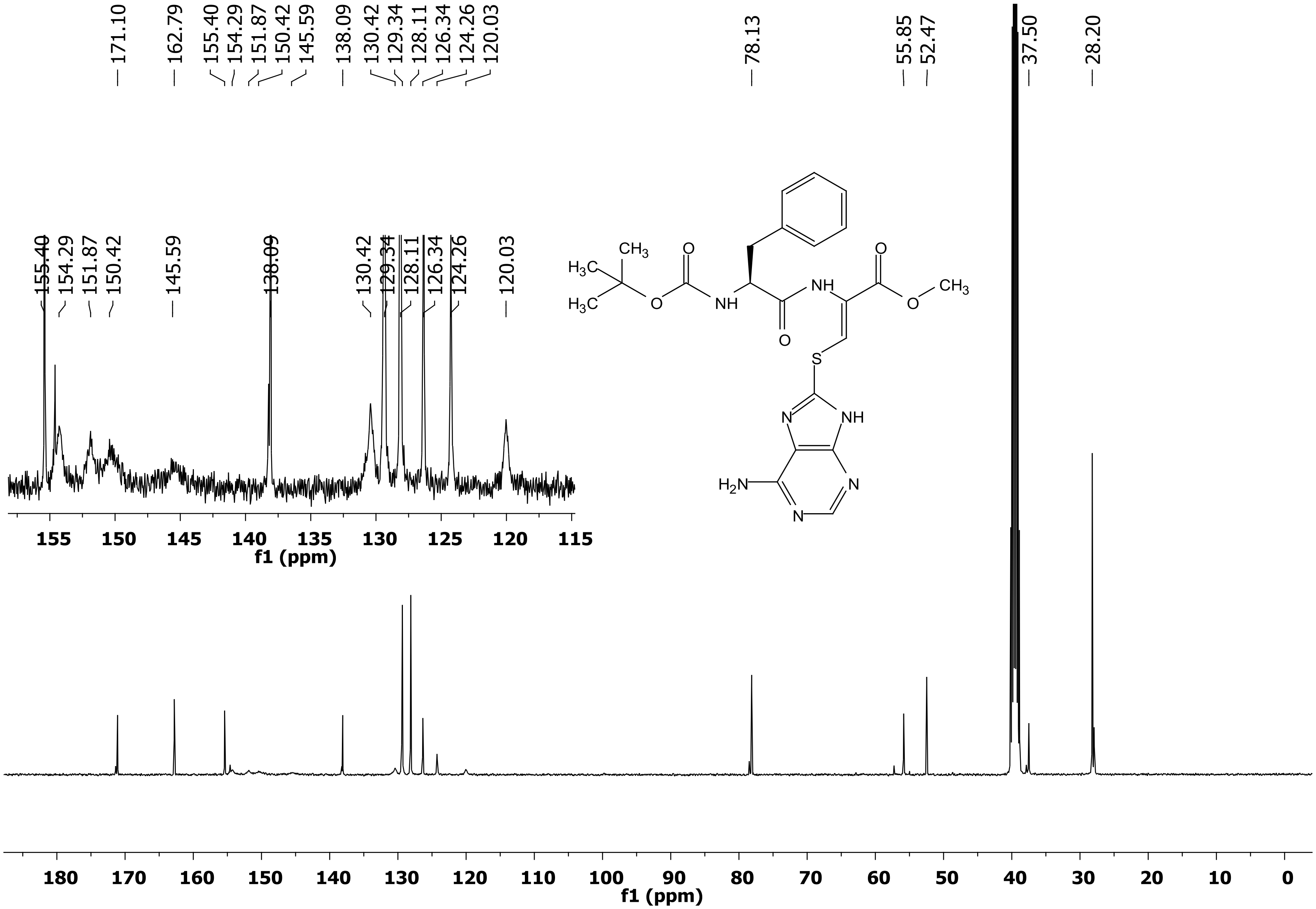
171.10
162.79
155.40
154.29
151.87
150.42
145.59
138.09
130.42
129.34
128.11
126.34
124.26
120.03
78.13
55.85
52.47
37.50
28.20
f1 (ppm)
f1 (ppm)
H_3C
CH_3
H_3C
O
NH
NH
O
O
O
CH_3
S
N
NH
H_2N
N
N

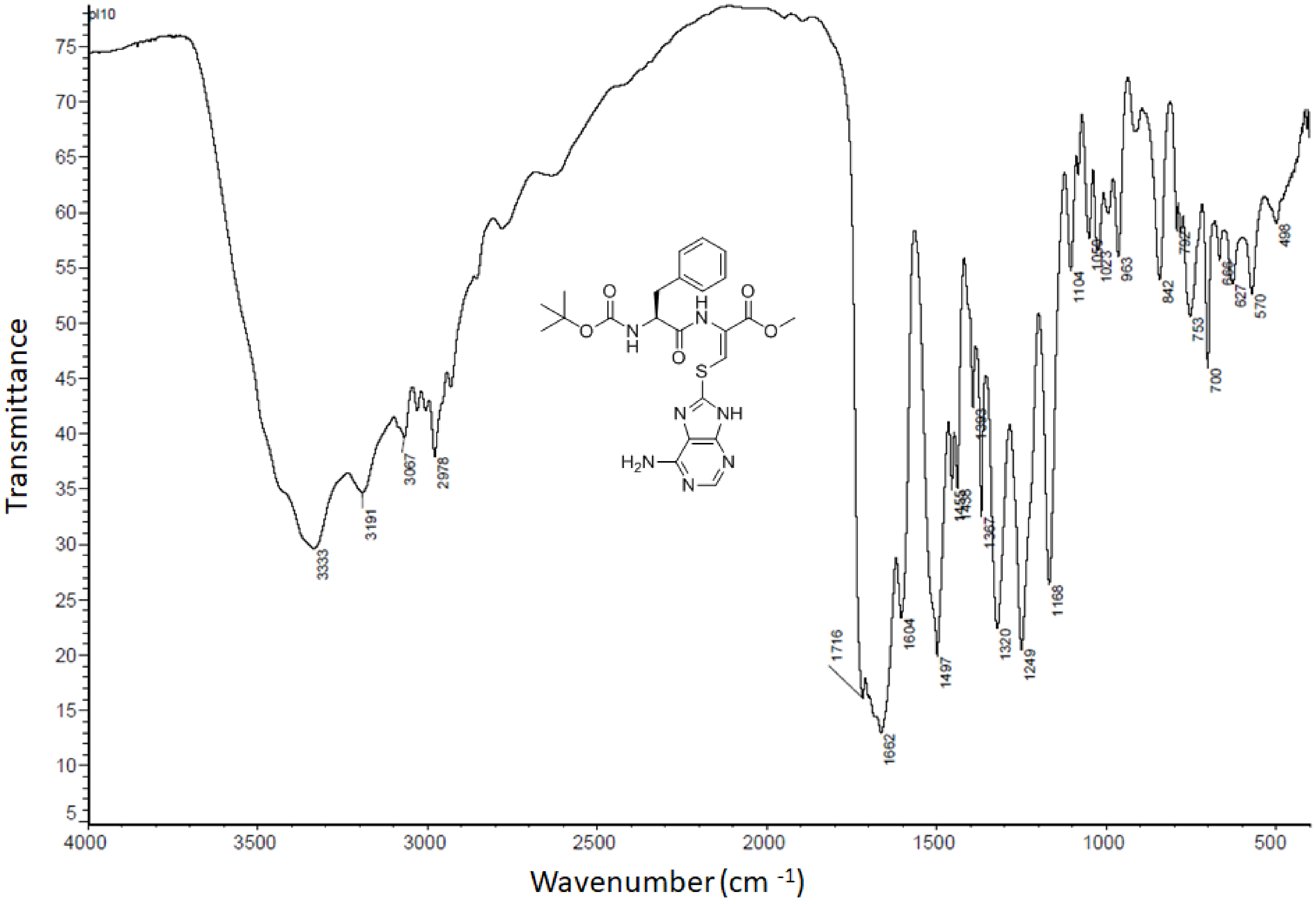

pl10
Transmittance
Wavenumber (cm -1)
75
70
65
60
55
50
45
40
35
30
25
20
15
10
5
4000
3500
3000
2500
2000
1500
1000
500
3333
3191
3067
2978
1716
1662
1604
1497
1367
1320
1249
1168
1104
1023
963
842
753
700
627
570
498
O
N
H
S
N
NH
H_2N

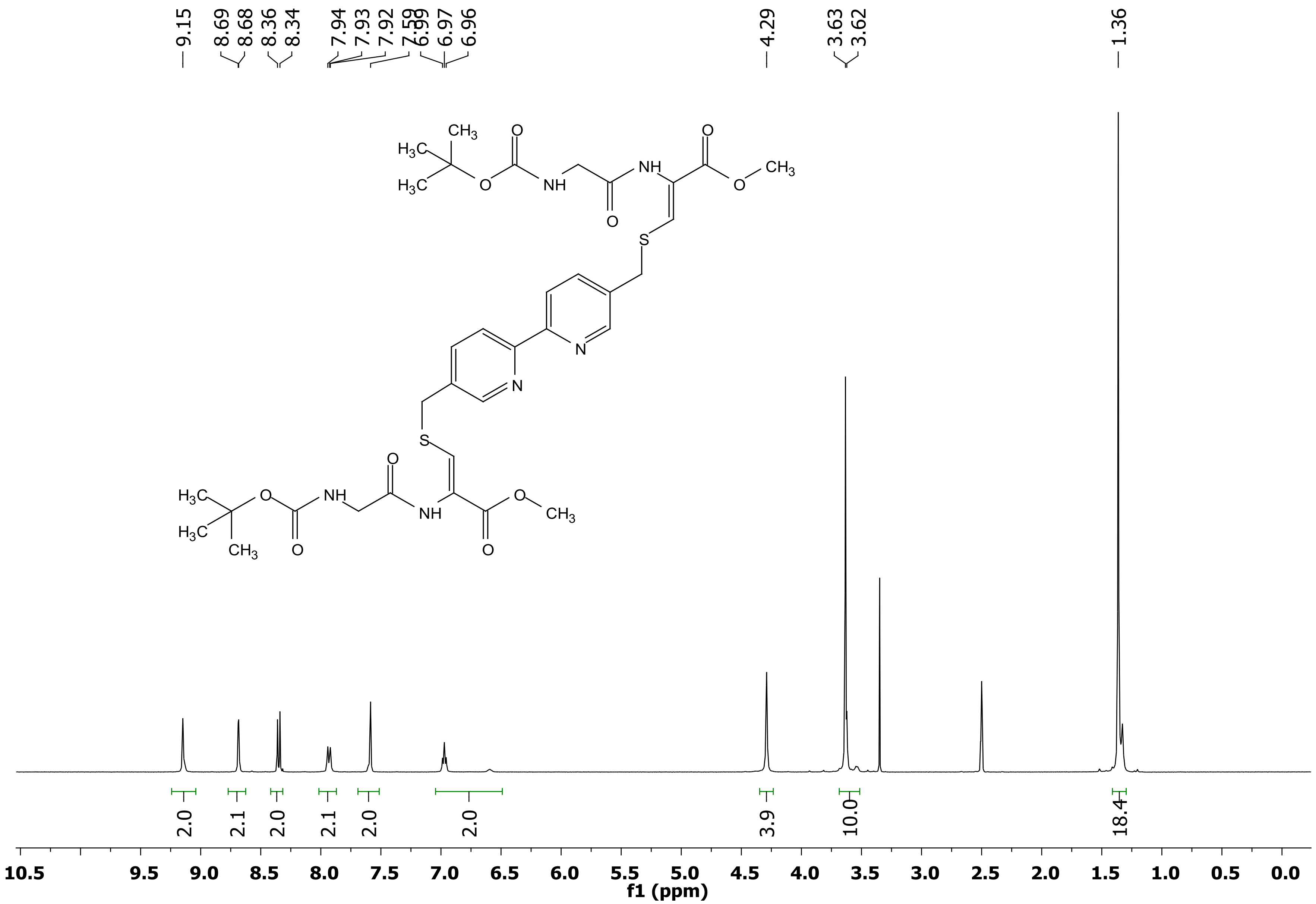
9.15
8.69
8.68
8.36
8.34
7.94
7.93
7.92
7.59
6.99
6.97
6.96
4.29
3.63
3.62
1.36
2.0
2.1
2.0
2.1
2.0
2.0
3.9
10.0
18.4
10.5
9.5
9.0
8.5
8.0
7.5
7.0
6.5
6.0
5.5
5.0
4.5
4.0
3.5
3.0
2.5
2.0
1.5
1.0
0.5
0.0
f1 (ppm)

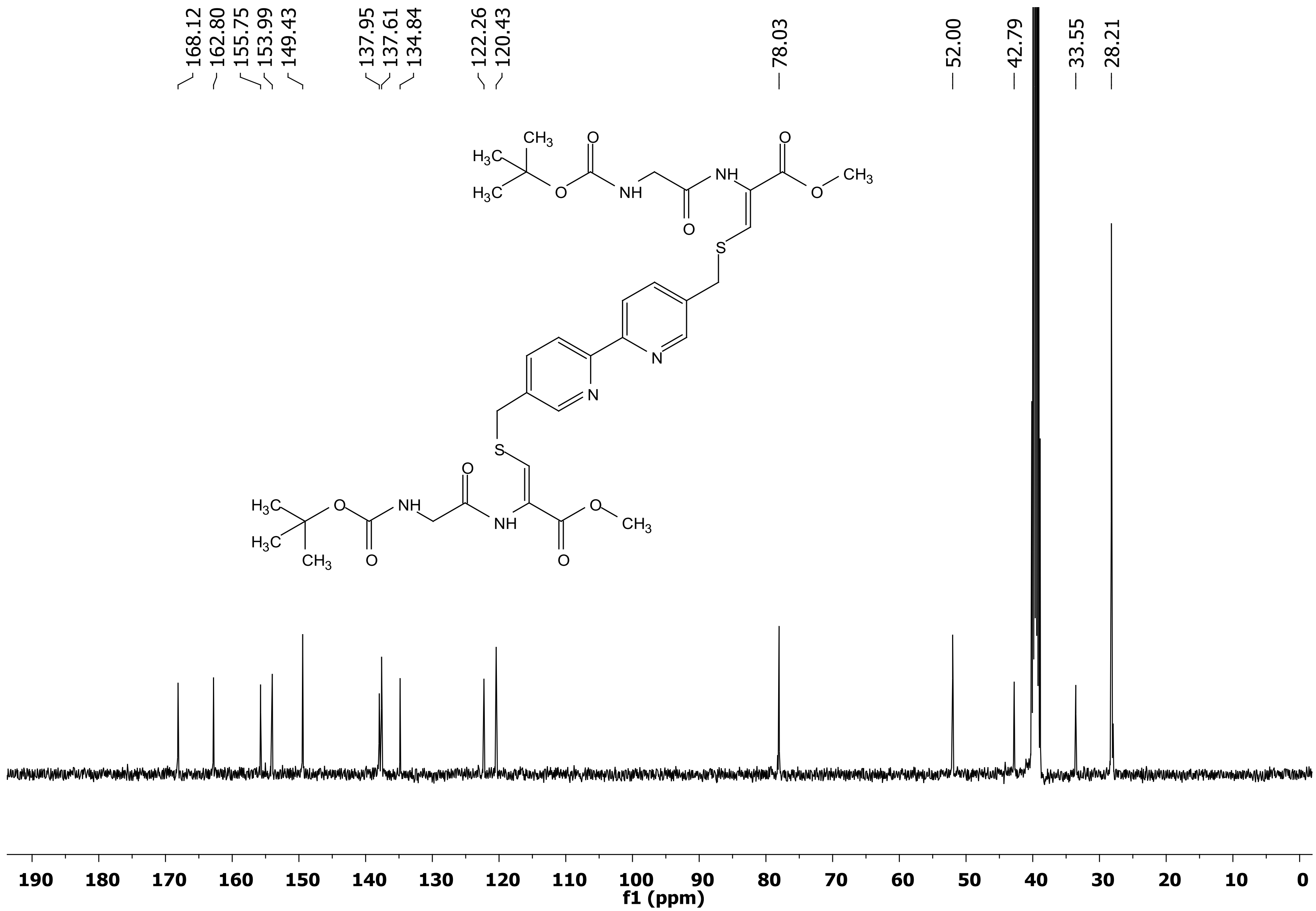

168.12
162.80
155.75
153.99
149.43
137.95
137.61
134.84
122.26
120.43
78.03
52.00
42.79
33.55
28.21
190 180 170 160 150 140 130 120 110 100 90 80 70 60 50 40 30 20 10 0
f1 (ppm)

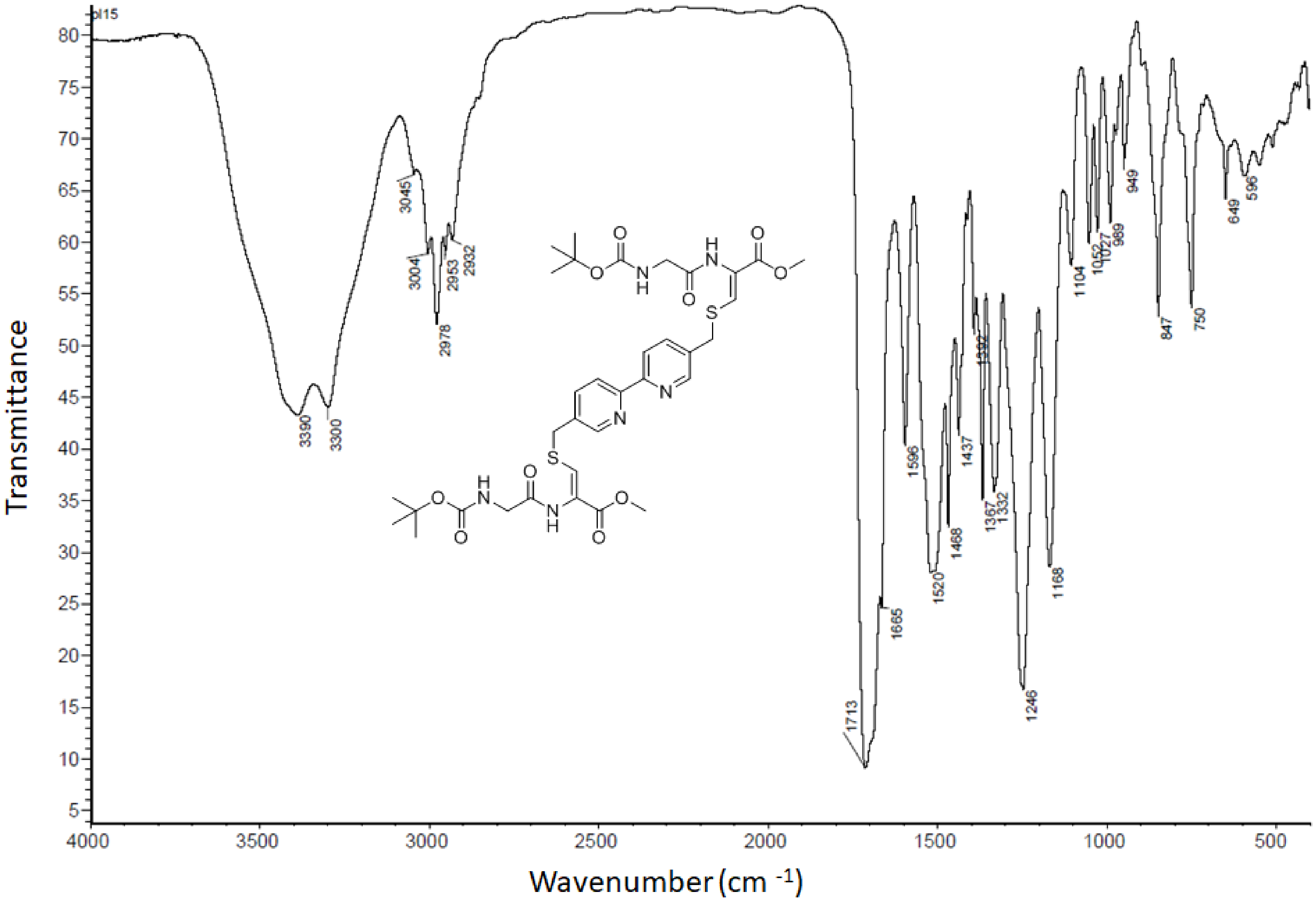

pl15
Transmittance
Wavenumber (cm -1)
3390
3300
3045
3004
2978
2953
2932
1713
1665
1596
1520
1468
1437
1392
1367
1332
1246
1168
1104
1052
1027
989
949
847
750
649
596

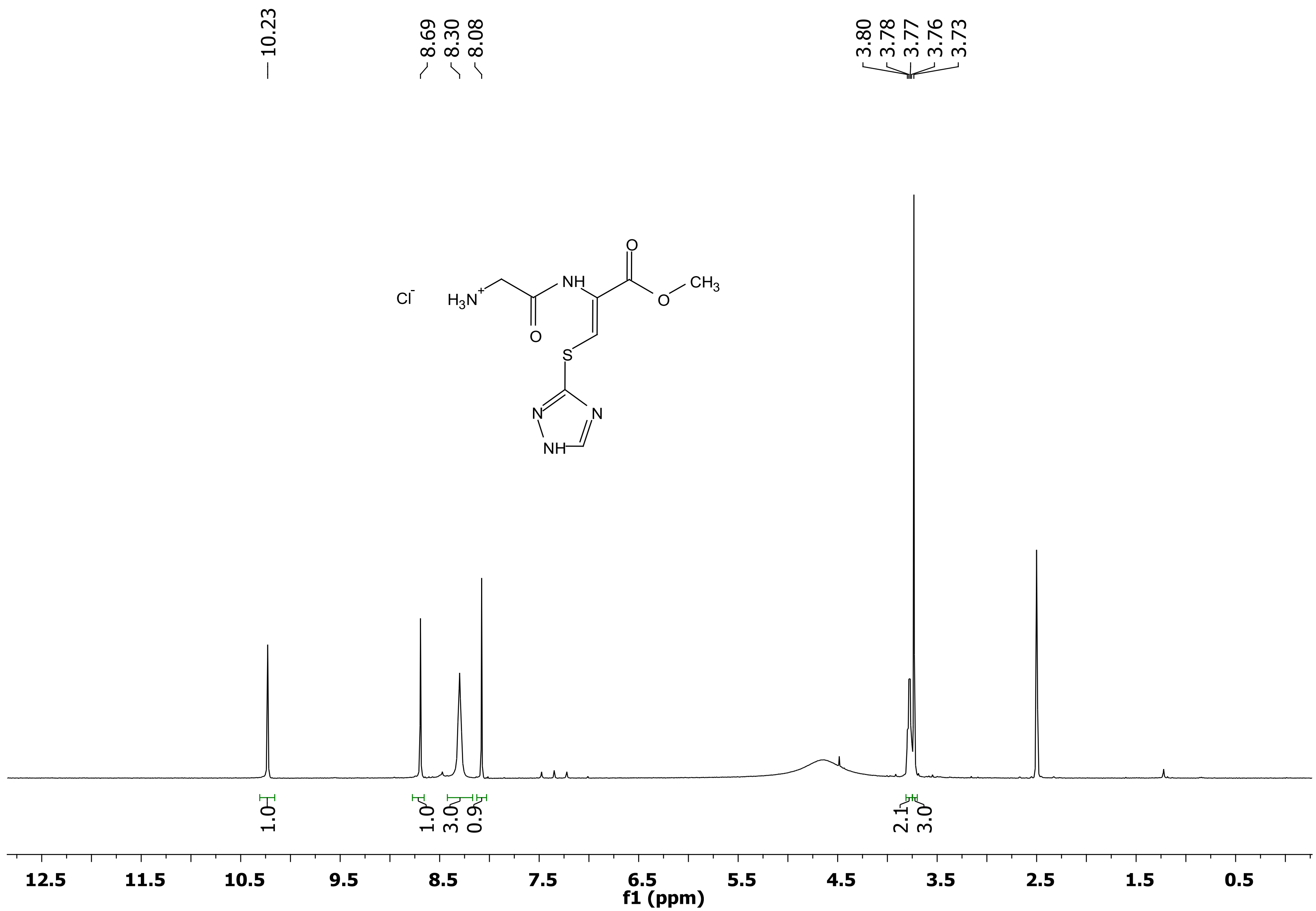
10.23
8.69
8.30
8.08
3.80
3.78
3.77
3.76
3.73
Cl-
H3N+
NH
O
O
O
CH3
S
N
N
NH
1.0
1.0
3.0
0.9
2.1
3.0
12.5
11.5
10.5
9.5
8.5
7.5
6.5
5.5
4.5
3.5
2.5
1.5
0.5
f1 (ppm)

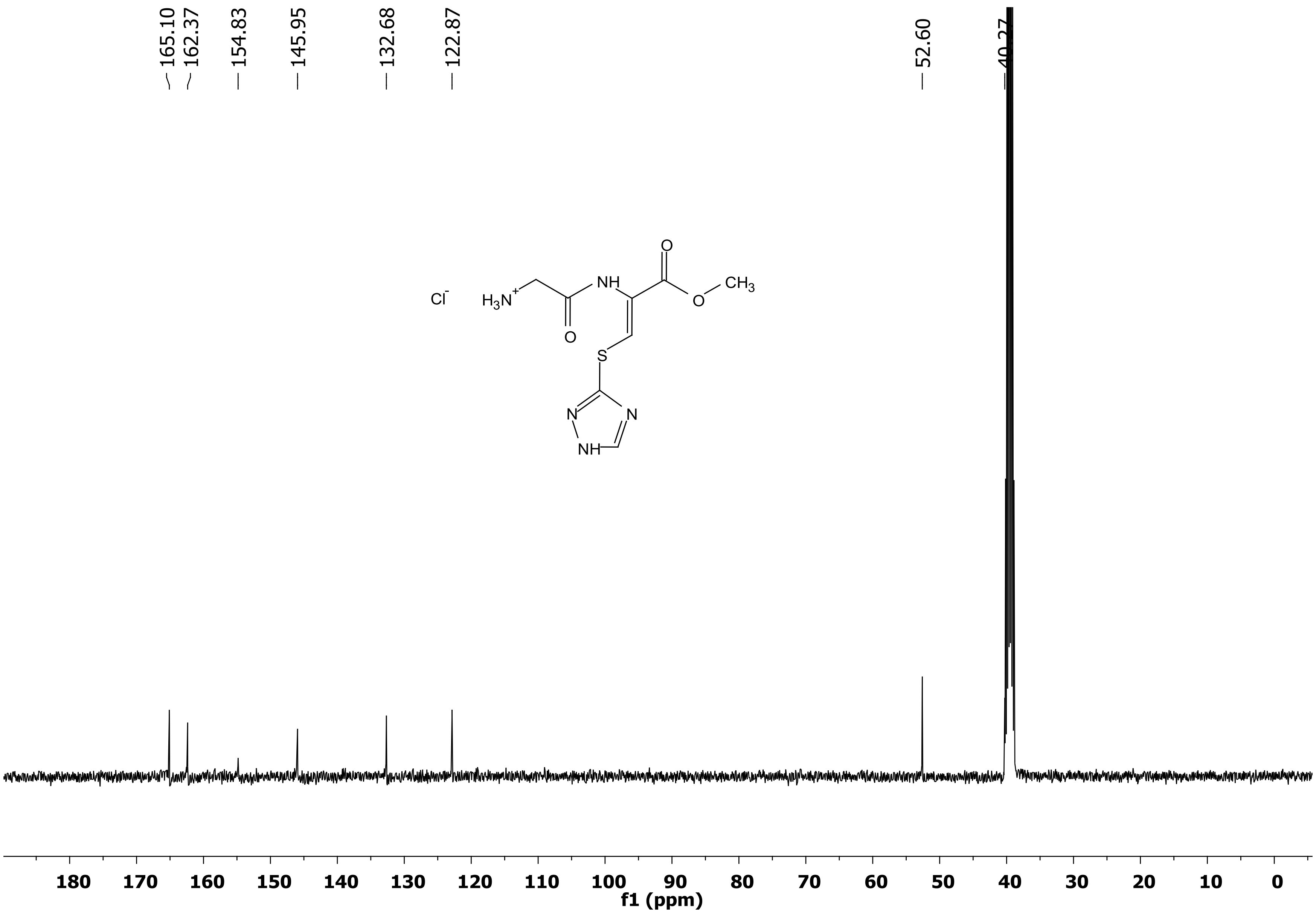

165.10
162.37
154.83
145.95
132.68
122.87
52.60
40.27
Cl⁻
H3N+
NH
O
O
O
CH3
S
N
N
NH
180 170 160 150 140 130 120 110 100 90 80 70 60 50 40 30 20 10 0
f1 (ppm)

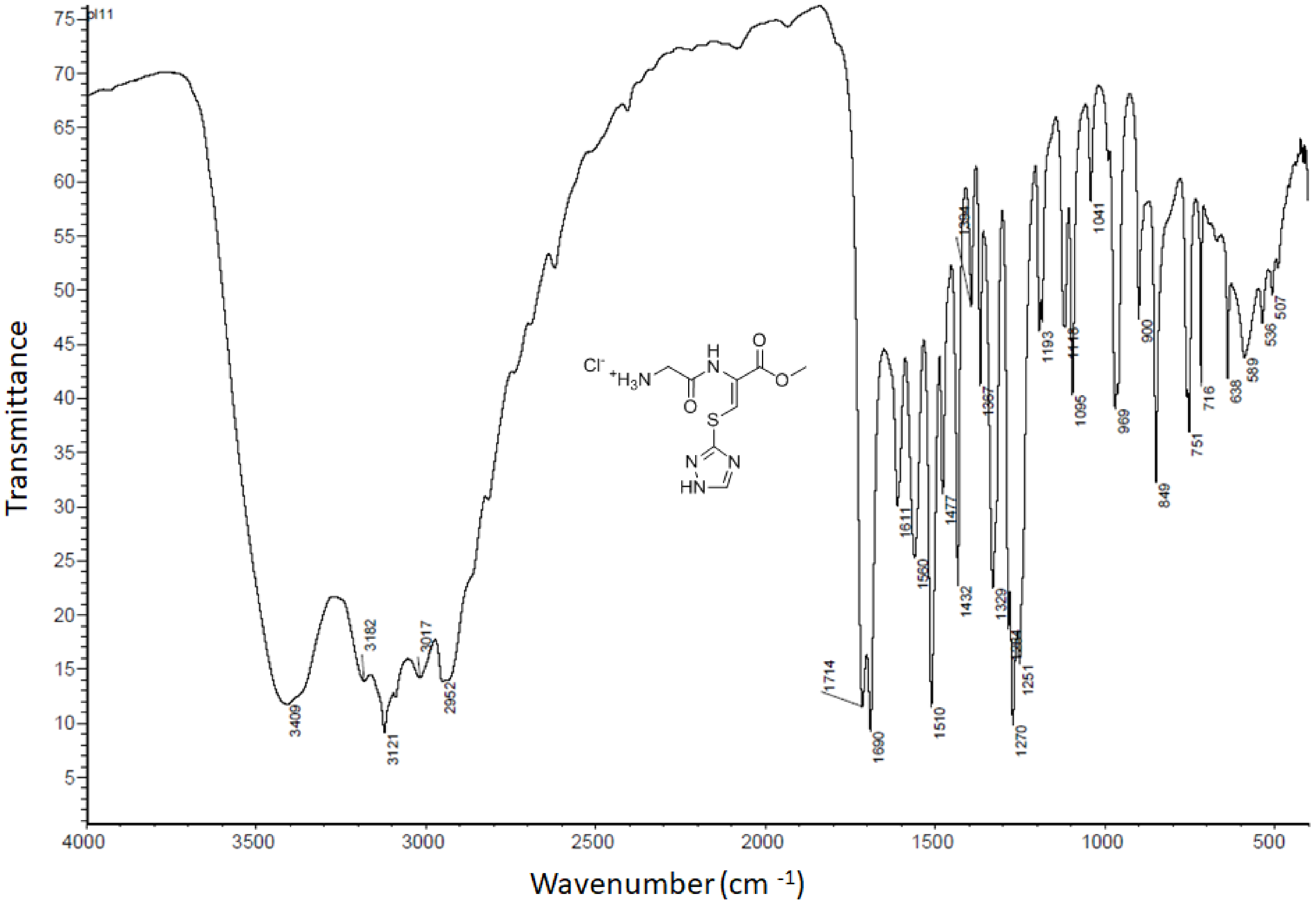

Transmittance
Wavenumber (cm -1)
pl11
3409
3182
3121
3017
2952
1714
1690
1611
1560
1510
1477
1432
1367
1329
1270
1251
1193
1116
1095
1041
969
900
849
751
716
638
589
536
507
Cl- +H3N
H
N
O
O
O
S
N
N
HN

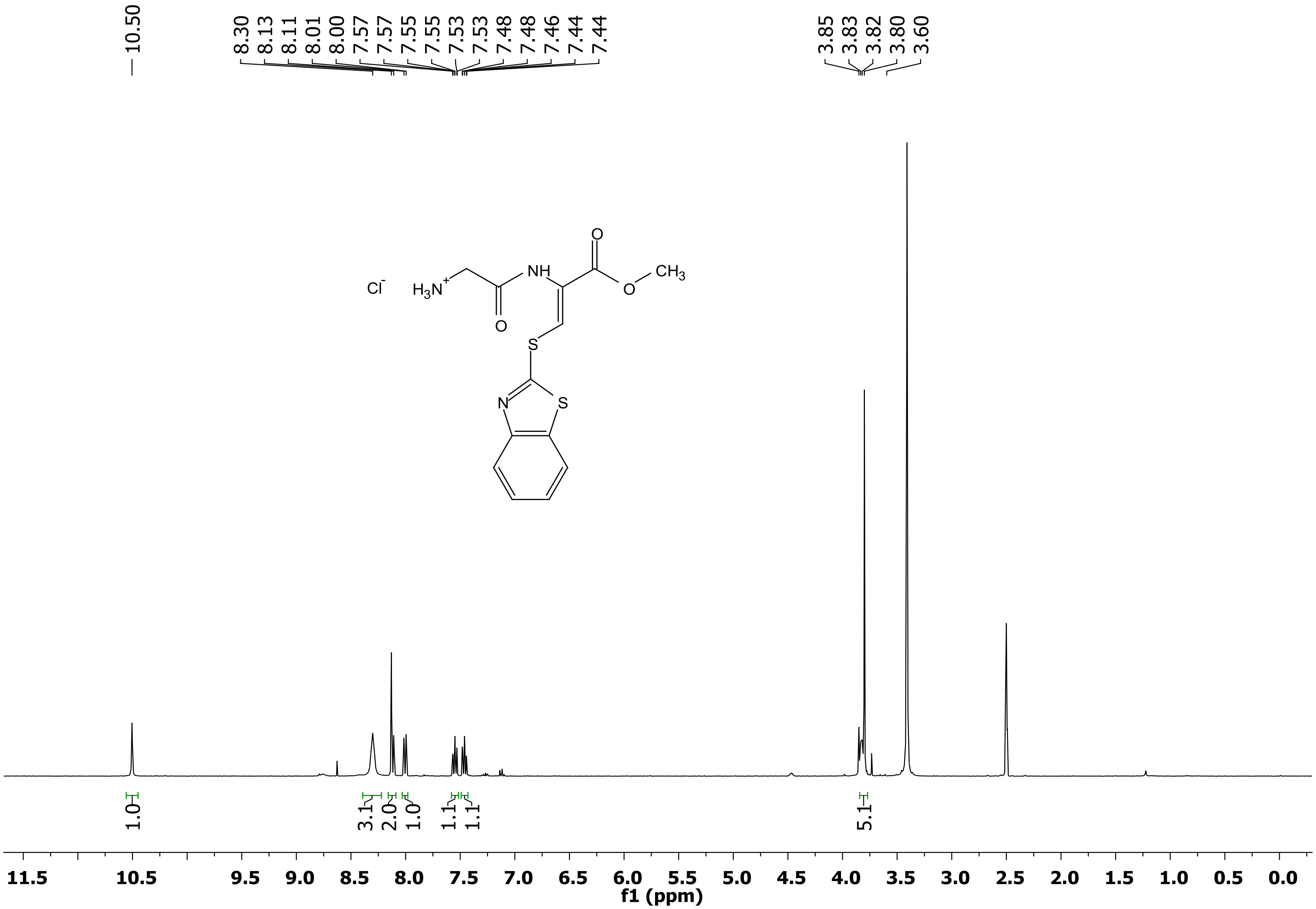

10.50
8.30
8.13
8.11
8.01
8.00
7.57
7.57
7.55
7.55
7.53
7.53
7.48
7.48
7.46
7.44
7.44
3.85
3.83
3.82
3.80
3.60
Cl-
H3N+
NH
O
O
CH3
O
S
N
S
1.0
3.1
2.0
1.0
1.1
1.1
5.1
11.5
10.5
9.5
9.0
8.5
8.0
7.5
7.0
6.5
6.0
5.5
5.0
4.5
4.0
3.5
3.0
2.5
2.0
1.5
1.0
0.5
0.0
f1 (ppm)

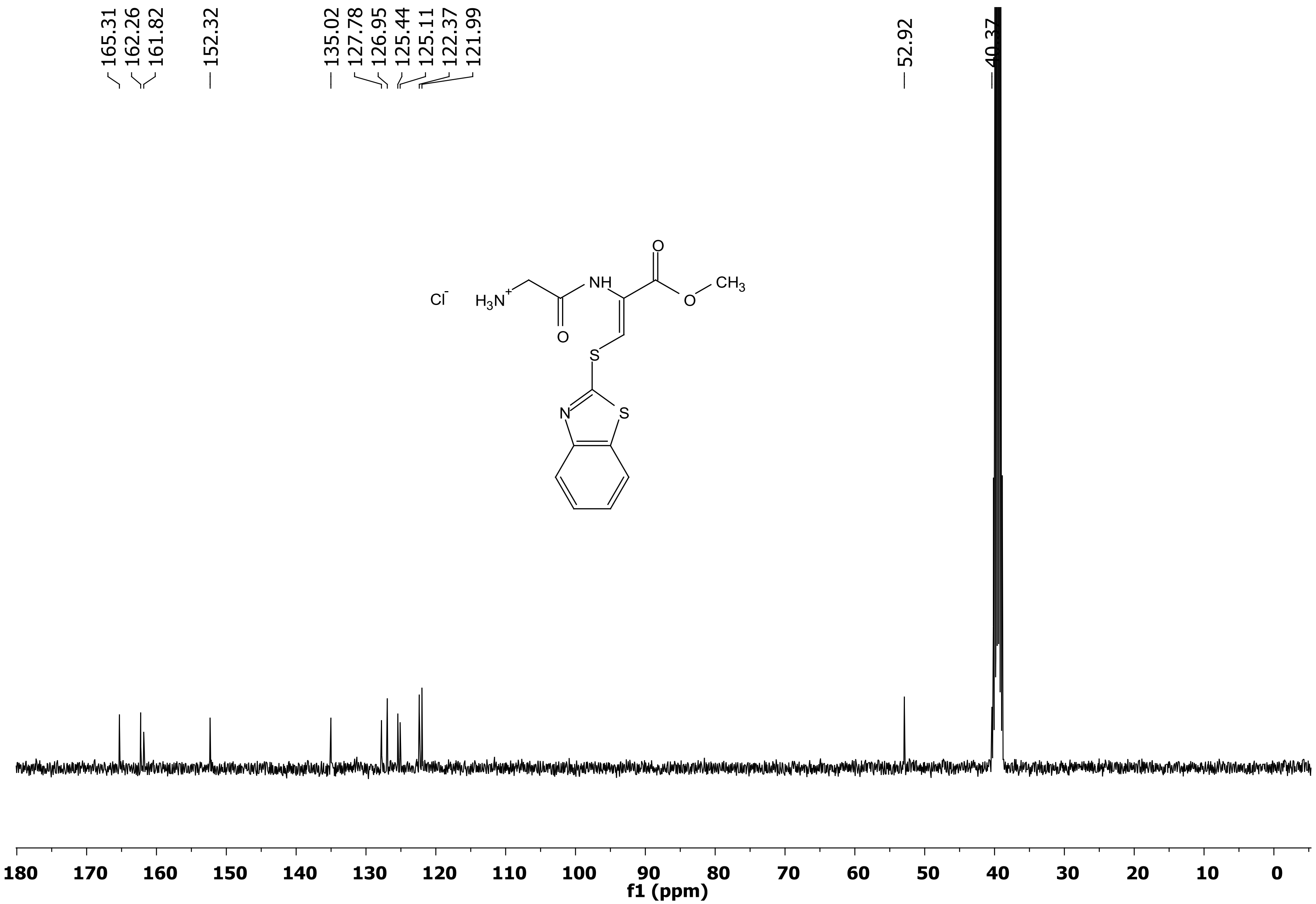
165.31
162.26
161.82
152.32
135.02
127.78
126.95
125.44
125.11
122.37
121.99
52.92
40.37
Cl⁻
H3N+
NH
O
O
O
CH3
S
N
S
180
170
160
150
140
130
120
110
100
90
80
70
60
50
40
30
20
10
0
f1 (ppm)

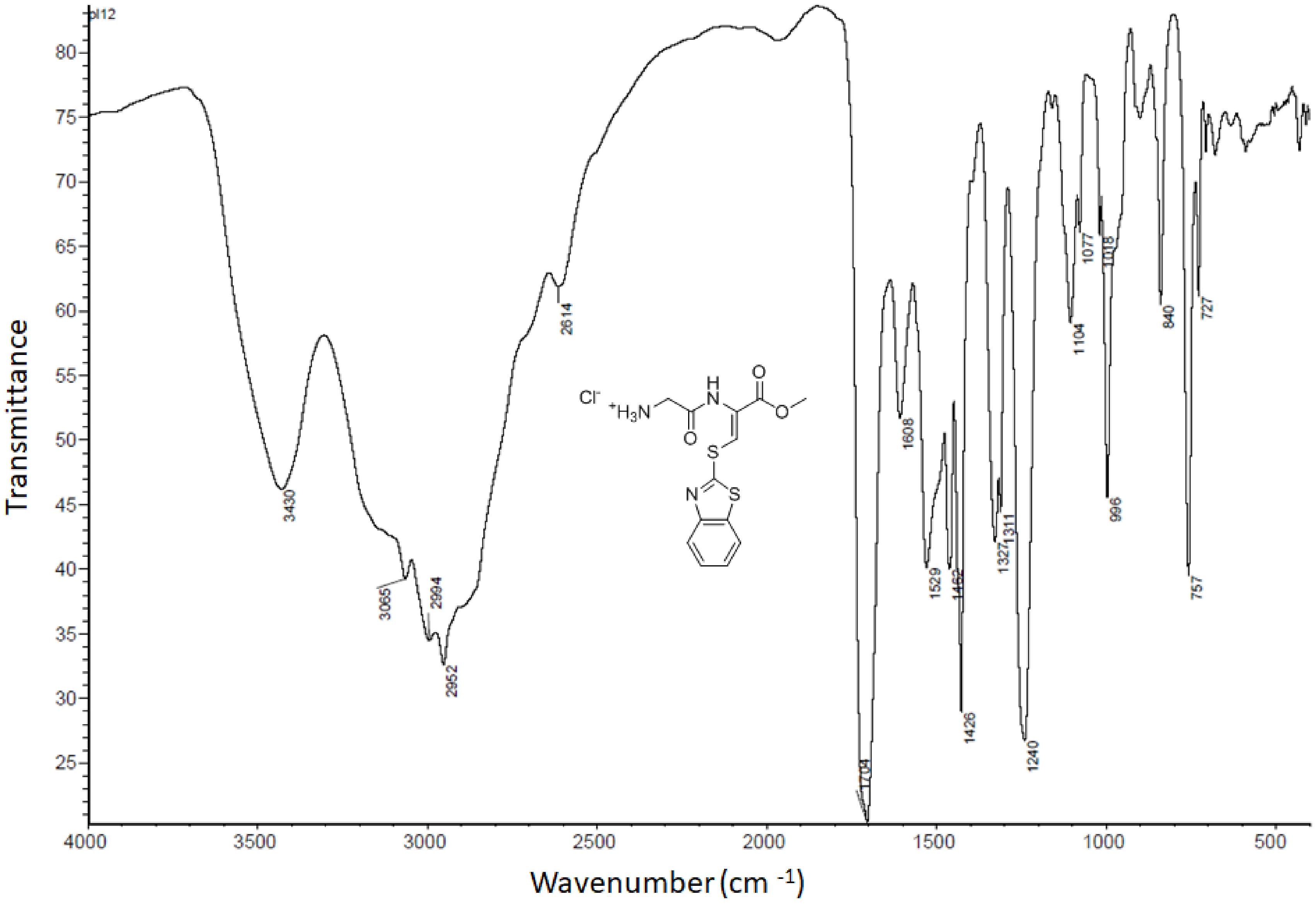

pl12
Transmittance
Wavenumber (cm -1)
3430
3065
2994
2952
2614
1704
1608
1529
1462
1426
1327
1311
1240
1104
1077
1018
996
840
757
727
Cl⁻ ⁺H3N

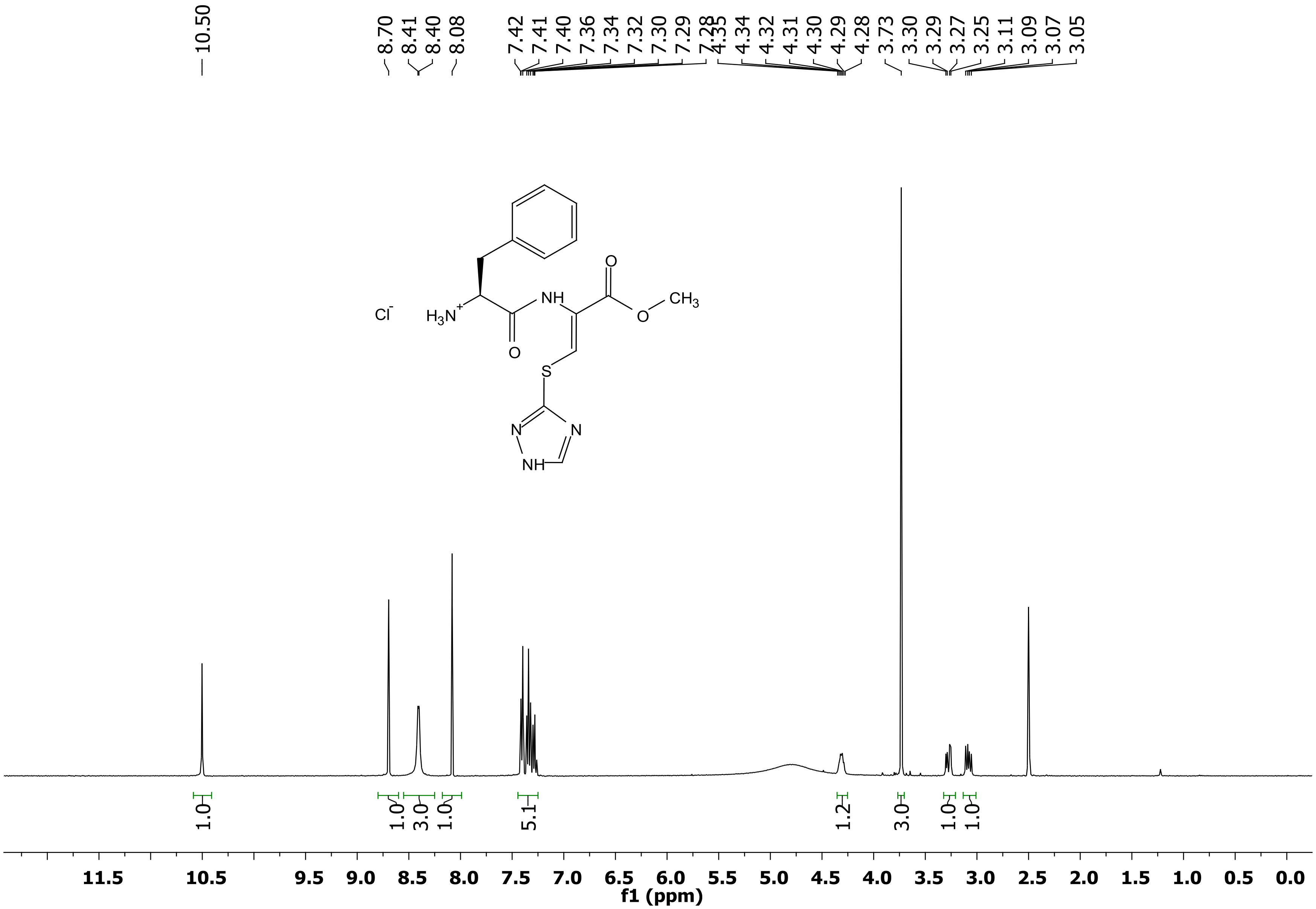
10.50
8.70
8.41
8.40
8.08
7.42
7.41
7.40
7.36
7.34
7.32
7.30
7.29
7.28
4.35
4.34
4.32
4.31
4.30
4.29
4.28
3.73
3.30
3.29
3.27
3.25
3.11
3.09
3.07
3.05
Cl⁻
H3N+
NH
O
O
O
CH3
S
N
N
NH
1.0
1.0
3.0
1.0
5.1
1.2
3.0
1.0
1.0
11.5
10.5
9.5
9.0
8.5
8.0
7.5
7.0
6.5
6.0
5.5
5.0
4.5
4.0
3.5
3.0
2.5
2.0
1.5
1.0
0.5
0.0
f1 (ppm)

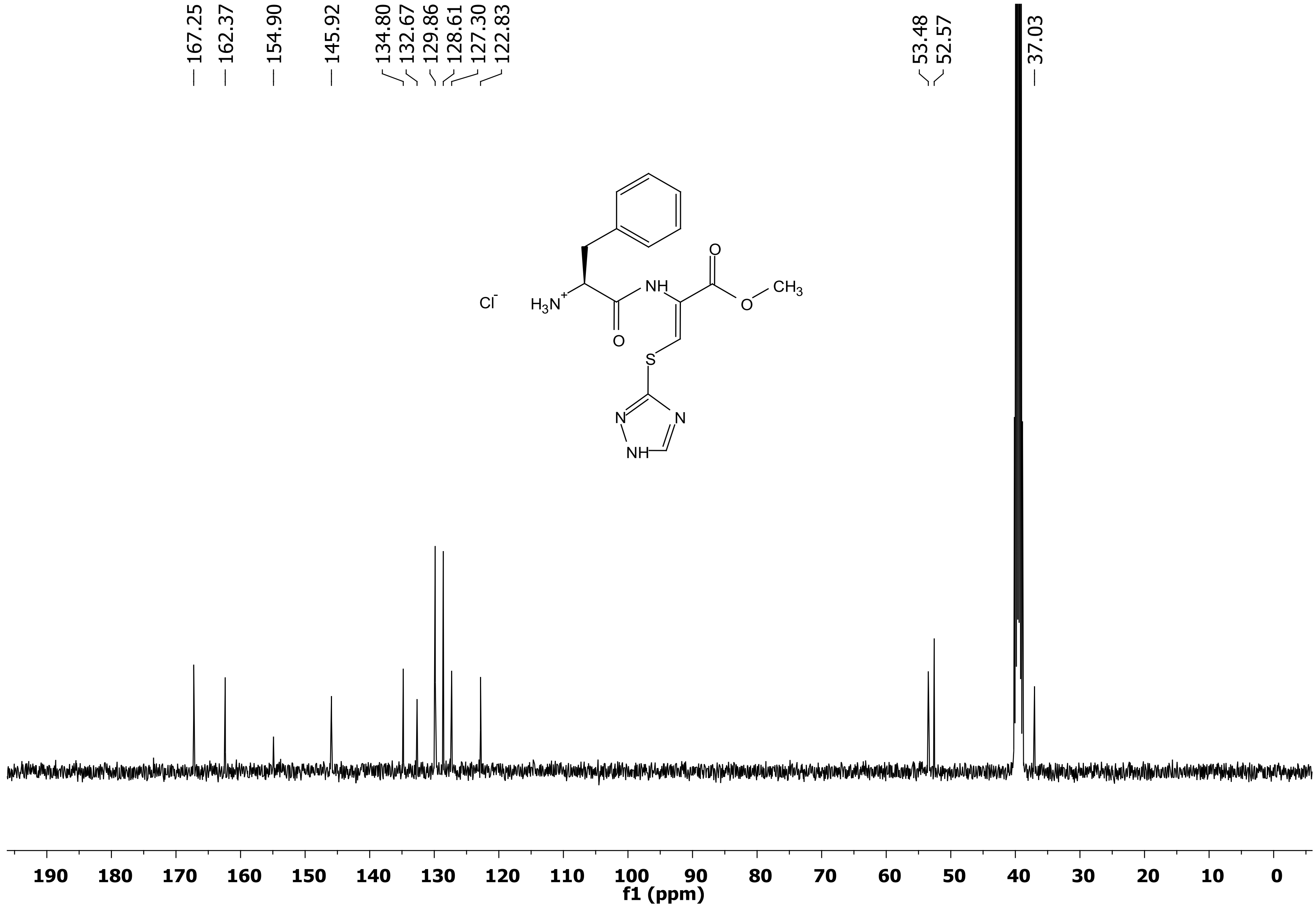

167.25
162.37
154.90
145.92
134.80
132.67
129.86
128.61
127.30
122.83
53.48
52.57
37.03
Cl
H3N
NH
O
O
O
CH3
S
N
N
NH
190
180
170
160
150
140
130
120
110
100
90
80
70
60
50
40
30
20
10
0
f1 (ppm)

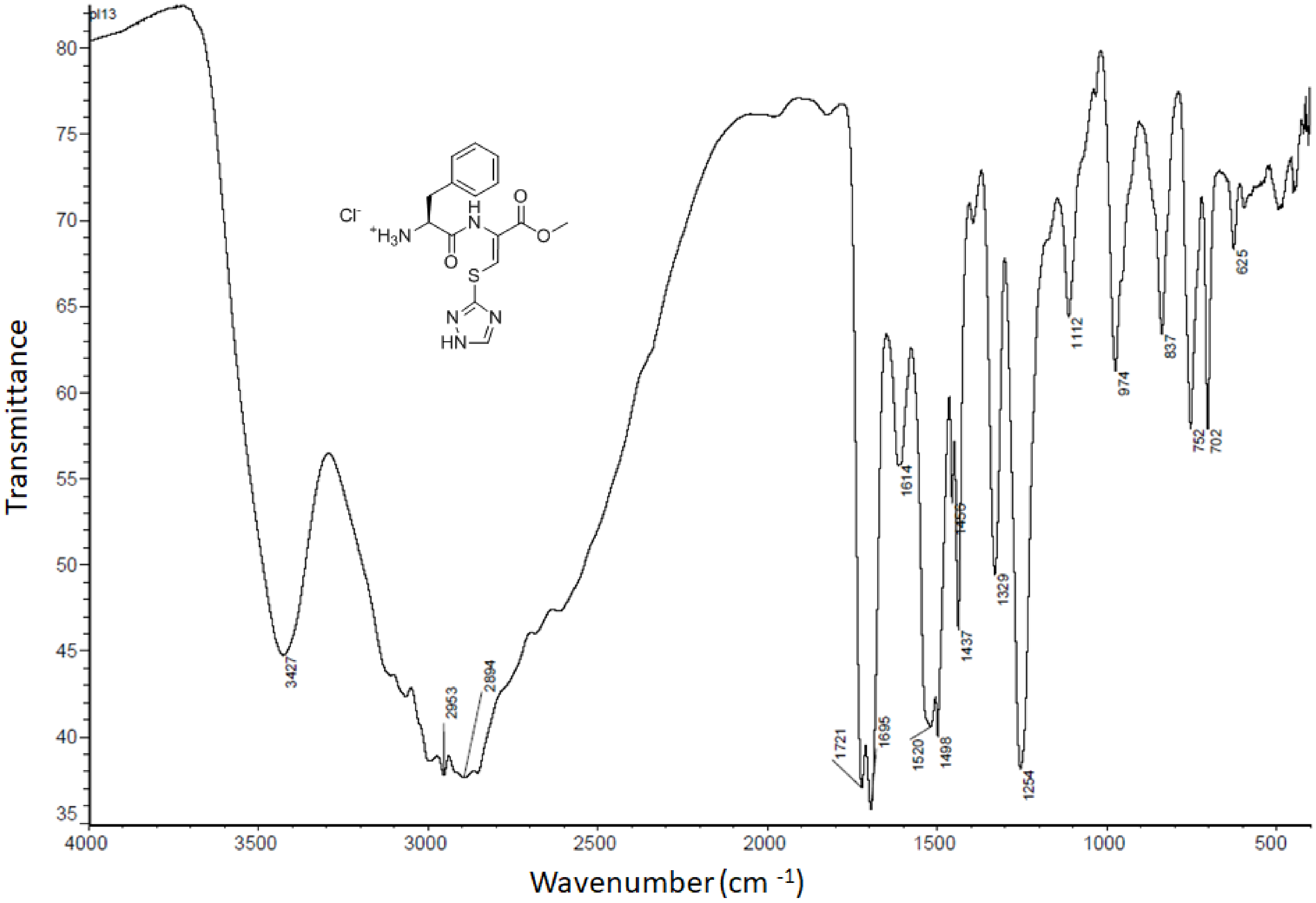
pl13
Transmittance
Wavenumber (cm -1)
3427
2953
2894
1721
1695
1614
1520
1498
1456
1437
1329
1254
1112
974
837
752
702
625
Cl-
+H3N
O
H
N
O
O
S
N
N
HN

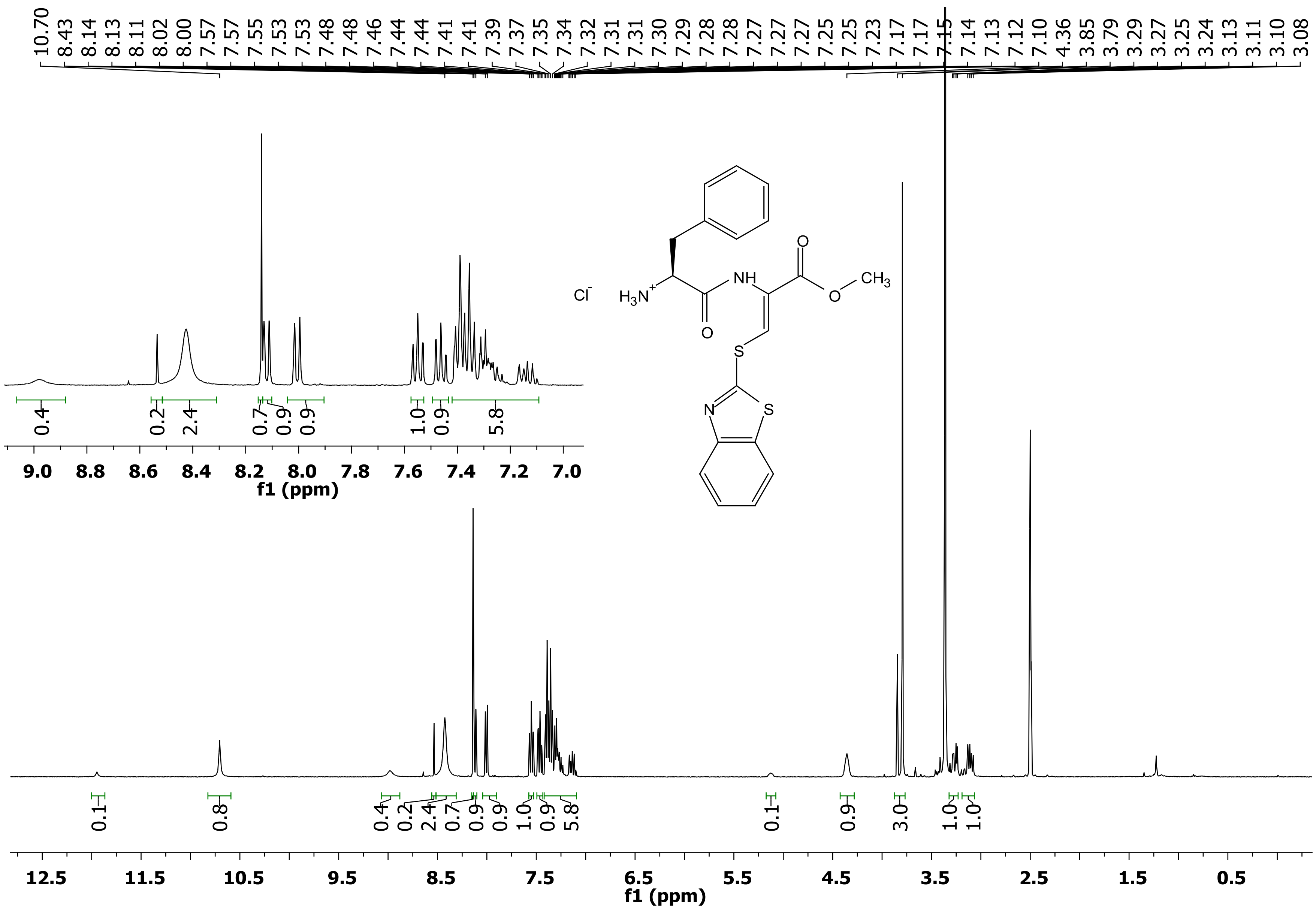

10.70 8.43 8.14 8.13 8.11 8.02 8.00 7.57 7.57 7.55 7.53 7.53 7.48 7.48 7.46 7.44 7.44 7.41 7.41 7.39 7.37 7.35 7.34 7.32 7.31 7.31 7.30 7.29 7.28 7.28 7.27 7.27 7.27 7.25 7.25 7.23 7.17 7.17 7.15 7.14 7.13 7.12 7.10 4.36 3.85 3.79 3.29 3.27 3.25 3.24 3.13 3.11 3.10 3.08
Cl-
H3N+
NH
O
O
O
CH3
S
N
S
0.4 0.2 2.4 0.7 0.9 0.9 1.0 0.9 5.8
9.0 8.8 8.6 8.4 8.2 8.0 7.8 7.6 7.4 7.2 7.0
f1 (ppm)
0.1 0.8 0.4 0.2 2.4 0.7 0.9 0.9 1.0 0.9 5.8 0.1 0.9 3.0 1.0 1.0
12.5 11.5 10.5 9.5 8.5 7.5 6.5 5.5 4.5 3.5 2.5 1.5 0.5
f1 (ppm)

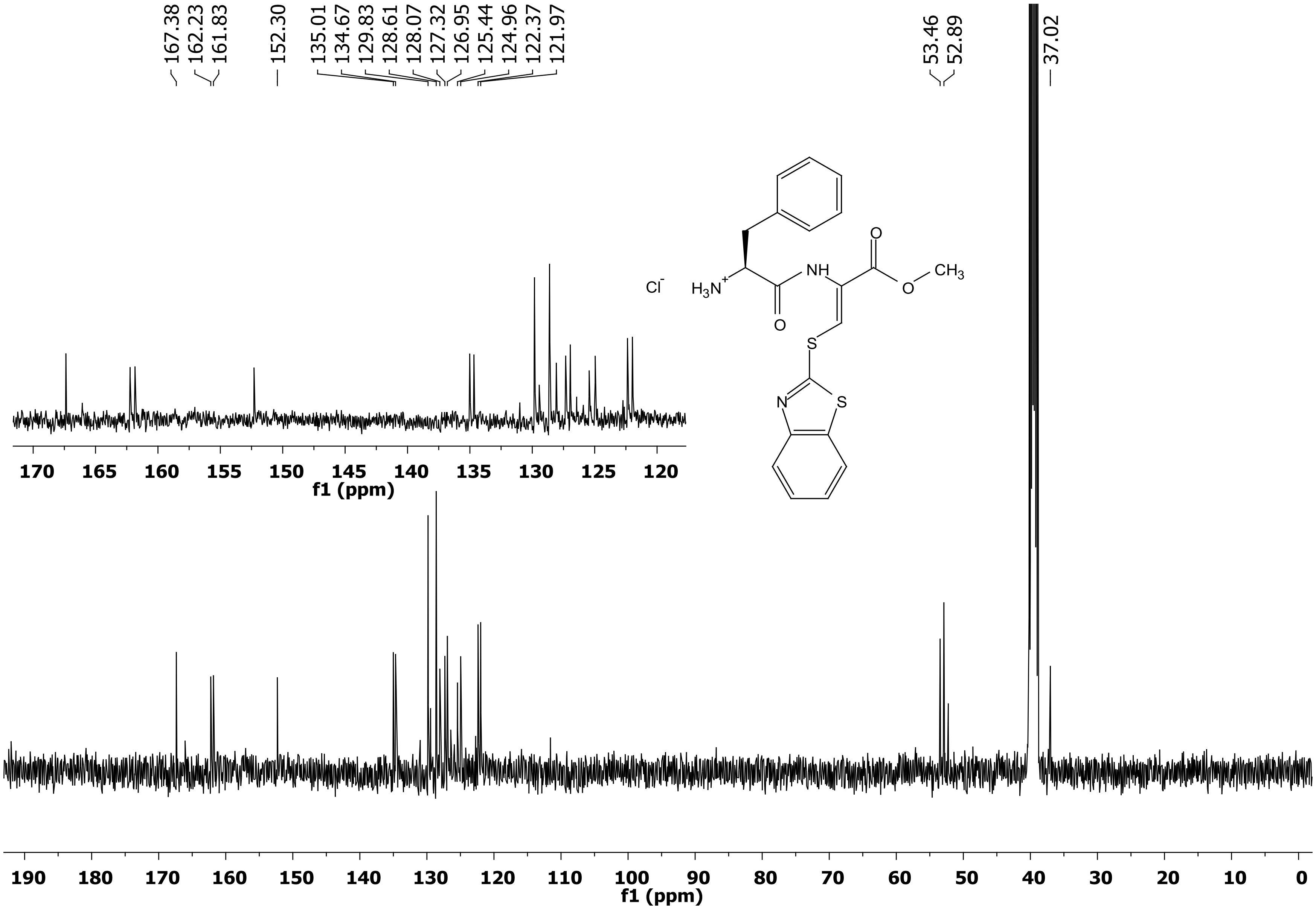
167.38
162.23
161.83
152.30
135.01
134.67
129.83
128.61
128.07
127.32
126.95
125.44
124.96
122.37
121.97
53.46
52.89
37.02
Cl-
H_3N^+
NH
O
O
O
CH_3
S
N
S
170 165 160 155 150 145 140 135 130 125 120
f1 (ppm)
190 180 170 160 150 140 130 120 110 100 90 80 70 60 50 40 30 20 10 0
f1 (ppm)

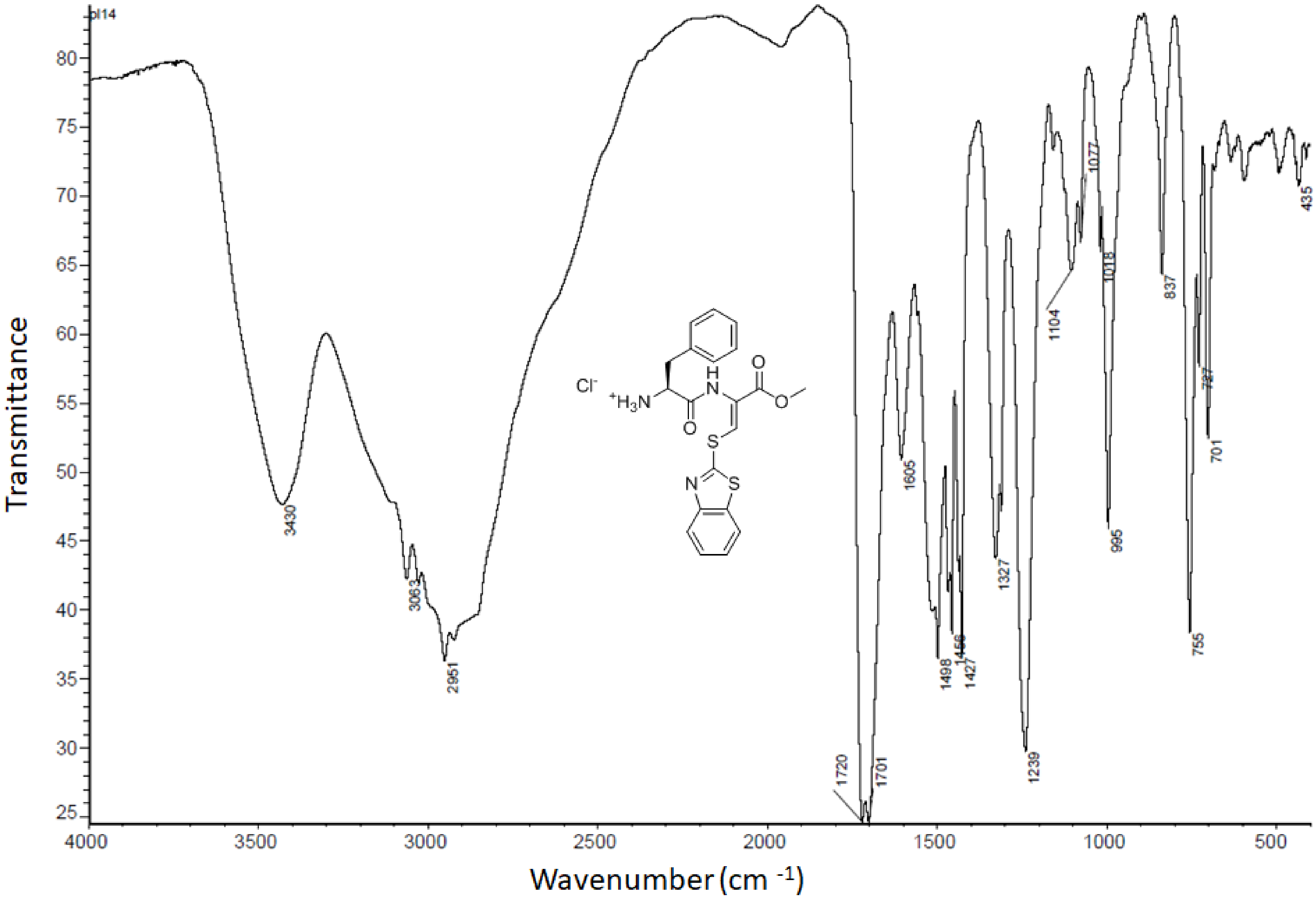
pl14
Transmittance
Wavenumber (cm -1)
4000
3500
3000
2500
2000
1500
1000
500
80
75
70
65
60
55
50
45
40
35
30
25
3430
3063
2951
1720
1701
1605
1498
1456
1427
1327
1239
1104
1077
1018
995
837
755
727
701
435
Cl-
+H3N
H
N
O
O
O
S
N
S
